# Dataset of 569 metagenome-assembled genomes from the caeca of multiple chicken breeds from commercial and backyard farming setups of Pakistan

**DOI:** 10.1016/j.dib.2024.110552

**Published:** 2024-05-23

**Authors:** Farrukh Saleem, Aqsa Ameer, Banaz Star-Shirko, Ciara Keating, Ozan Gundogdu, Umer Zeeshan Ijaz, Sundus Javed

**Affiliations:** a Department of Biosciences, COMSATS University Islamabad, Pakistan; b Water & Environment Research Group, University of Glasgow, Mazumdar-Shaw Advanced Research Centre, Glasgow, United Kingdom; c Department of Engineering, Durham University, Durham, DH1 3LE, United Kingdom; d School of Biodiversity, One Health, and Veterinary Medicine, College of Medical, Veterinary and Life Sciences, University of Glasgow, Glasgow, United Kingdom; e Department of Infection Biology, Faculty of Infectious and Tropical Diseases, London School of Hygiene and Tropical Medicine, London, United Kingdom; f Department of Molecular and Clinical Cancer Medicine, University of Liverpool, Liverpool, United Kingdom; g College of Science and Engineering, University of Galway, Ireland

## Abstract

This article focuses the recovery of prokaryotic organisms including bacteria and archaea from 9 different groups of chicken raised in different farm setups in Pakistan. The groups comprise of three different breeds (Broilers, White Layers, and Black Australorp) of chicken raised in different farming setups that include antibiotic-free control, commercial (open and controlled shed), and backyard farms. We have recovered 569 Metagenomics-Assembled Genomes (MAGs) with a completeness of ≥50 % and contamination of ≤10 %. For each MAG, functional annotations were obtained that include KEGG modules, carbohydrate active enzymes (CAZymes), peptidases, geochemical cycles, antibiotic resistance genes, stress genes, and virulence genes. Furthermore, two different sets of Single Copy Genes (SCGs) were used to construct the phylogenetic trees. Based on the reconstructed phylogeny, phylogenetic gain of each MAG is calculated to give an account of novelty.

Specifications Table

**Table:** 

Subject	Biological Sciences: Microbiology: Microbiome
Specific subject area	Caecal microbial communities of three chicken breeds (Broilers, White Layers, and Black Australorp).
Type of data	FASTA files/Tables
How the data was acquired	Illumina NovaSeq X Plus platform (10B FC) (375Gb per lane) ensuring ∼9.15 GB reads per sample (41 samples); Illumina TruSeq ensuring ∼20 M reads per samples for 4 Broiler Control samples using 2 × 100 bp reads.
Data format	Raw and Analysed
Description of data collection	The genomic DNA was extracted from the caecal samples collected from three chicken breeds, Broilers, White Layers, and Black Australorp. For Broiler and White Layer, three different farming setups were used on commercial scale: 1. controlled house / shed system; 2. open house / shed system; and 3. birds reared as antibiotic free control group. For Black Australorp breed, samples were collected from antibiotic free control group, free range rearing setup and from commercial open shed. This breed is a preferred choice by locals to keep as a major backyard chicken breed. Furthermore, it is acclimatized to the local environment and is known to be resistant to many diseases. Note that, on commercial scale in Pakistan, only open sheds are functioning and rearing in closed controlled houses is few and far between.
Data source location	City/Country: Islamabad/Pakistan; Latitude and Longitude: 33.6844° N, 73.0479° E
Data accessibility	Figshare: http://dx.doi.org/10.6084/m9.figshare.24901884

## 1 Value of the Data

• The data offers insights into the genomic content of bacterial and archaeal candidates found in the cecum of various commercial and backyard breeds of chickens raised in diverse farming environments.
• Evaluating the functional potential of genomes will be valuable in determining which chicken breed and farming setup are effective in managing routine outbreaks. The data is relevant for a comparative genomic study involving 569 distinct prokaryotic candidates.
• Data will also help in resistome (antibiotic resistance genes) analysis as there are genotypic and phenotypic variations, to highlight the farming setup which is at high risk of emergence of antibiotic resistance.
• Data will help in improving the management strategies for different poultry farming environments in Pakistan.

## 2 Background

The purpose of the study is to compare different poultry rearing setups and their impact on the caecal microbiome and resistome of the locally raised commercial breeds. The experiment was initiated by procuring day old chicks of Broiler, White Layer and Black Australorp from a local market in Islamabad, Pakistan. All breeds were reared as antibiotic free control group in separate semi-controlled rooms and fed with standard feed without prophylactic or remedial antibiotic administration till maturity (22 weeks for White Layer and Black Australorp; ∼8 weeks for Broilers when their weight reached 1.5 kg). Additionally, samples were also collected from commercial open and controlled sheds for Broiler and White Layer. For Black Australorp, the samples were collected from open shed and free range setup. Irrespective of farm setup, within a breed, the age of bird at sampling is same.

## 3 Data Description

**Fig. 1:**
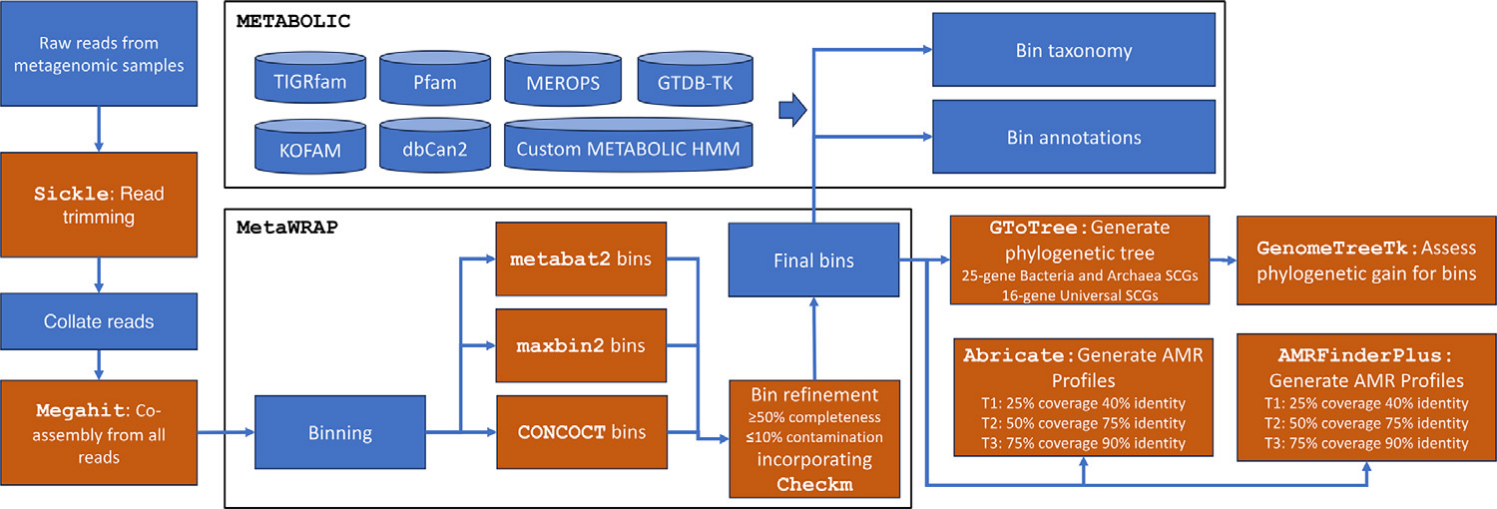
Diagram illustrating the workflow used for generation of MAGs.

**Fig. 2:**
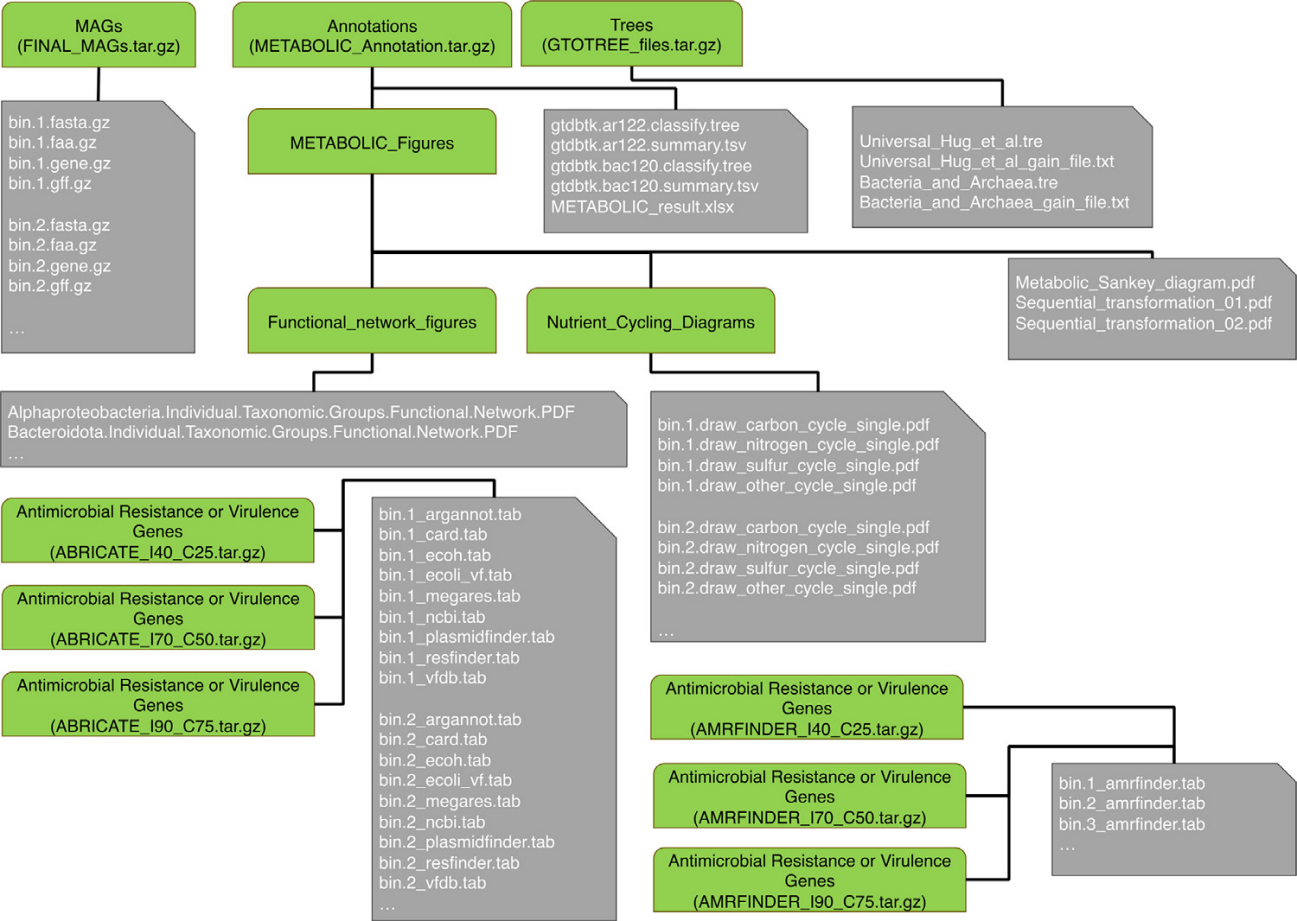
Diagram illustrating the structure of the repository. Nine archives contain sequencing data and annotation for the MAGs. The green rounded corner nodes denote directories or compressed directories, while the grey nodes represent individual files. Ellipses indicate the repetition of these files for each MAG.

The workflow is given in Fig. 1. The resulting dataset has a repository structure depicted in Fig. 2, encompassing a total of 569 metagenome-assembled genomes (MAGs). For each MAG number (x), the corresponding files are provided in the FINAL_MAGs main directory:

• bin.x.fasta.gz → Obtained genomic sequence of the contigs that make the MAG
• bin.x.gene.gz → Obtained genomic sequences for genes
• bin.x.faa.gz → Obtained protein sequences for genes
• bin.x.gff.gz → Comprehensive annotation of MAGs, detailing various types of features along with their respective locations on the length of contigs

The METABOLIC_result.xlsx in the METABOLIC_Annotations main directory comprises following 6 spreadsheets:

• HMMHitNum → The occurrence or absence of customized Hidden Markov Model (HMM) profiles, the frequency of HMM profile identification within a MAG, and the Open Reading Frame(s) (ORF) representing the identified protein.
• FunctionHit → The presence or absence of sets of proteins, individually identified in the sheet titled “HMMHitNum”. For each MAG, the functions are marked as either “Present” or “Absent”.
• KEGGModuleHit → Each MAG is annotated with modules from the KEGG database, categorized by metabolic functions. The status of each module in a MAG is indicated as either “Present” or “Absent”.
• KEGGModuleStepHit → The occurrence or absence of modules from the KEGG database within each (MAG), delineated into the individual steps comprising the module. For each MAG, the module steps are identified as “Present” or “Absent”.
• dbCAN2Hit →The annotation results from dbCAN2 for all MAGs, including CAZyme numbers and hits. Each MAG is presented with two columns indicating the frequency of CAZyme identification and the corresponding Open Reading Frame(s) representing the protein.
• MEROPSHit → The search results for MEROPS peptidases, including peptidase numbers and hits, are provided for each MAG. Two columns are allocated for each MAG, indicating the frequency of peptidase identification and the corresponding ORF(s) representing the protein.

The GTDB-Tk files in the METABOLIC_Annotations main directory are provided as:

• gtdtbtk.ar122.classify.tree → Phylogenetic tree in Newick format representing MAGs classified as archaea.
• gtdtbtk.ar122.summary.tsv → Taxonomic categorization of MAGs identified as archaea across various taxonomic ranks
• gtdtbtk.bac120.classify.tree → Phylogenetic tree in Newick format depicting MAGs classified as bacteria.
• gtdtbtk.bac120.summary.tsv → Taxonomic categorization of MAGs identified as bacteria across various taxonomic ranks.

The Nutrient_Cycling_Diagrams directory is the sub-directory of METABOLIC-Figures of the METABOLIC_Annotations main directory. It includes files for each MAG (x replaces the MAG number), where a red arrow signifies the presence, and a black arrow indicates the absence of a pathway step, respectively:

• bin.x.draw_other_cycle_single.PDF
• bin.x.draw_carbon_cycle_single.PDF
• bin.x.draw_nitrogen_cycle_single.PDF
• bin.x.draw_sulfur_cycle_single.PDF

Furthermore, the directory includes summary diagrams for pathways on a community scale

• draw_nitrogen_cycle_total.PDF
• draw_other_cycle_total.PDF
• draw_carbon_cycle_total.PDF
• draw_sulfur_cycle_total.PDF

Two sequential transformation diagrams, Sequential_transformation_01.pdf and Sequential_transformation_02.pdf, are available. These diagrams summarize and visualize MAG numbers and coverages, potentially involved in the sequential transformation of both inorganic and organic compounds. The Metabolic_Sankey_diagram.pdf illustrates the function fractions contributed by various microbial groups in a given community.

The Functional_network_figures directory is the sub-directory of METABOLIC-Figures of the METABOLIC_Annotations main directory. It includes diagrams that depict metabolic connections of biogeochemical cycle steps at both the phylum and community levels.

The GTOTREE_files main directory contains the following files:

• Bacteria_and_Archaea_gain_file.txt → Phylogenetic gain, both in absolute and percentage terms, was computed for each MAG relative to all other MAGs.
• Bacteria_and_Archaea.tre → Phylogenetic tree in Newick format derived from 25 gene SCGs for MAGs.
• Universal_Hug_et_al_gain_file.txt → Phylogenetic gain, measured in both absolute and percentage terms, was calculated for each MAG relative to all other MAGs. This calculation serves as a means to determine novelty.
• Universal_Hug_et_al.tre → Phylogenetic tree in Newick format derived from 16 gene SCGs for MAGs.

ABRICATE_I40_C25, ABRICATE_I70_C50, and ABRICATE_I90_C75 main directories all contain the following files repeated for each MAG (x replaces the MAG number):

• bin.x_argannot.tab → Antimicrobial Resistance (AMR) genes detected using ARG-ANNOT server [1].
• bin.x_card.tab → AMR genes detected using Comprehensive Antibiotic Resistance Database (CARD) [2].
• bin.x_ecoh.tab → Genes detected through the EcOH database of O- and H- surface antigens of *Escherichia coli* [3].
• bin.x_ecoli_vf.tab → Virulence factors for *E. coli* using the database available at https://github.com/phac-nml/ecoli_vf
• bin.x_megares.tab → Antimicrobial drug, biocide, and metal resistance genes detected using MEGARes 2.0 [4].
• bin.x_ncbi.tab → Genes detected using Bacterial antimicrobial resistance reference gene database maintained at https://www.ncbi.nlm.nih.gov/bioproject/PRJNA313047
• bin.x_plasmidfinder.tab → Detection of whole plasmid sequences from members of the family *Enterobacteriaceae*. [5]
• bin.x_resfinder.tab → AMR genes detected using RESFINDER [6].
• bin.x_vfdb.tab → Genes detected through the virulence factor database (VFDB) [7].

AMRFINDER_I40_C25, AMRFINDER_I70_C50, and AMRFINDER_I90_C75 main directories all contain the following files repeated for each MAG (x replaces the MAG number):

• bin.x_amrfinder.tab → Genes detected using AMRFinderPlus [8] that contains resistance, stress response, and virulence genes.

## 4 Experimental Design, Materials and Methods

### 4.1 Sample collection

The caecal samples from the antibiotic free control groups were collected after euthanizing the chicken and stored and −80 °C. Meanwhile, caecal sample from selected chicken breeds reared in different farming setups (controlled shed, open shed and free range) with variable antibiotic usage were also collected aseptically after euthanasia. Five samples from broiler and white layer chicken rearing in commercial controlled and open sheds were collected. For Black Australorp, five samples collected from each free range and commercial open shed. All the samples were stored immediately at −80 °C. The 45 samples (including a negative blank control) comprise of:

• Broiler antibiotic free control (*n* = 4)
• Broiler controlled shed (*n* = 5)
• Broiler open shed (*n* = 5)
• White Layer antibiotic free control (*n* = 5)
• White Layer controlled shed (*n* = 5)
• White Layer open shed (*n* = 5)
• Black Australorp antibiotic free control (*n* = 5)
• Black Australorp open shed = 5
• Black Australorp free range = 5

### 4.2 DNA extraction

DNA was extracted using the Invitrogen PureLink™ Microbiome DNA Purification Kit following manufacturer's instructions, followed by quality check through NanoDrop spectrophotometer. Quality genomic DNA indicates over 50 ng/µl per sample (sufficient for library preparation), with confirmation of purity checked via 260/280 ratio, and selection of samples as close to 1.8 as possible. All samples passed the threshold and generated libraries for sequencing.

### 4.3 Shotgun sequencing

Genomic DNA was normalised to 5 ng/µl with Elution Buffer (EB) (10 mM Tris–HCl). 0.5 µl of Tagmentation Buffer (TB1) was mixed with 0.5 µl Bead Linked Transposomes (BLT) (Illumina Catalogue No. 20,018,704) and 4 µl PCR grade water in a master mix. Aliquots of 5 µl were added to wells of a 96 well plate. 2 µl of normalised DNA (10 ng total) was pipette mixed with the 5 µl of the tagmentation mix and heated to 55⁰C for 15 mins in a PCR block. A PCR master mix was made up using 10 µl KAPA 2 G Fast Hot Start Ready Mix (Merck Catalogue No. KK5601) and 2 µl PCR grade water per sample. 12 µl of this mastermix was added to each well to be used in a 96-well plate. 1 µl of 10 µM 8 bp Unique Dual Indexes were added to each well. Finally, the 7 µl of Tagmentation mix was added and mixed. The PCR was run with 72⁰C for 3 min, 95⁰C for 1 min, 14 cycles of 95⁰C for 10 s, 55⁰C for 20 s and 72⁰C for 3 min. The libraries were quantified using the Promega QuantiFluor® dsDNA System (Catalogue No. E2670) and run on a GloMax® Discover Microplate Reader. Libraries were pooled following quantification in equal quantities. The final pool was double-SPRI size selected between 0.5 and 0.7X bead volumes using sample purification beads (Illumina® DNA Prep, (M) Tagmentation (96 Samples, IPB), 20,060,059). The final pool was quantified on a Qubit 3.0 instrument and run on a D5000 ScreenTape (Agilent Catalogue No. 5067–5579) using the Agilent Tapestation 4200 to calculate the final library pool molarity. Sequencing was performed using an Illumina NovaSeq X Plus platform (10B FC) (375Gb per lane) ensuring ∼9.15 GB reads per sample.

### 4.4 Recovery of metagenomic-assembled genomes

For a set of 45 metagenomic samples, the sequencing center provided adapter-trimmed reads. The workflows used on these samples is given in Fig. 1. The raw metagenomics reads underwent quality trimming using Sickle v1.200 [9]. This involved removing reads where the average Phred quality fell below 20 and retaining paired-end reads with a post-trimming length exceeding 50 bp. This gave us a total of 1230,968,126 reads from all samples with statistics given in Supplementary Table S1. We aggregated both the forward and reverse reads and conducted a co-assembly for all samples using Megahit. The assembly was performed with the parameters: **–k-list 27,47,67,87 –kmin-1pass -m 0.95 –min-contig-len 1000** [10]. This gave us a total of 1331,681 contigs, a total of 3274,466,202 base pairs (bp), maximum of 285,598 bp, average length of 2459 bp, and an N50 score of 2820 bp. We then used MetaWRAP pipeline [11] (using **metawrap binning -o INITIAL_BINNING -t 48 -a final.contigs.fa –metabat2 –maxbin2 –concoct** READS_DIRECTORY/*.fastq) and binned the contigs using three different binning algorithms i.e. metabat2 (1095 bins) [12], maxbin2 (907 bins) [13], and CONCOCT (398 bins) [14].

**Fig. 3:**
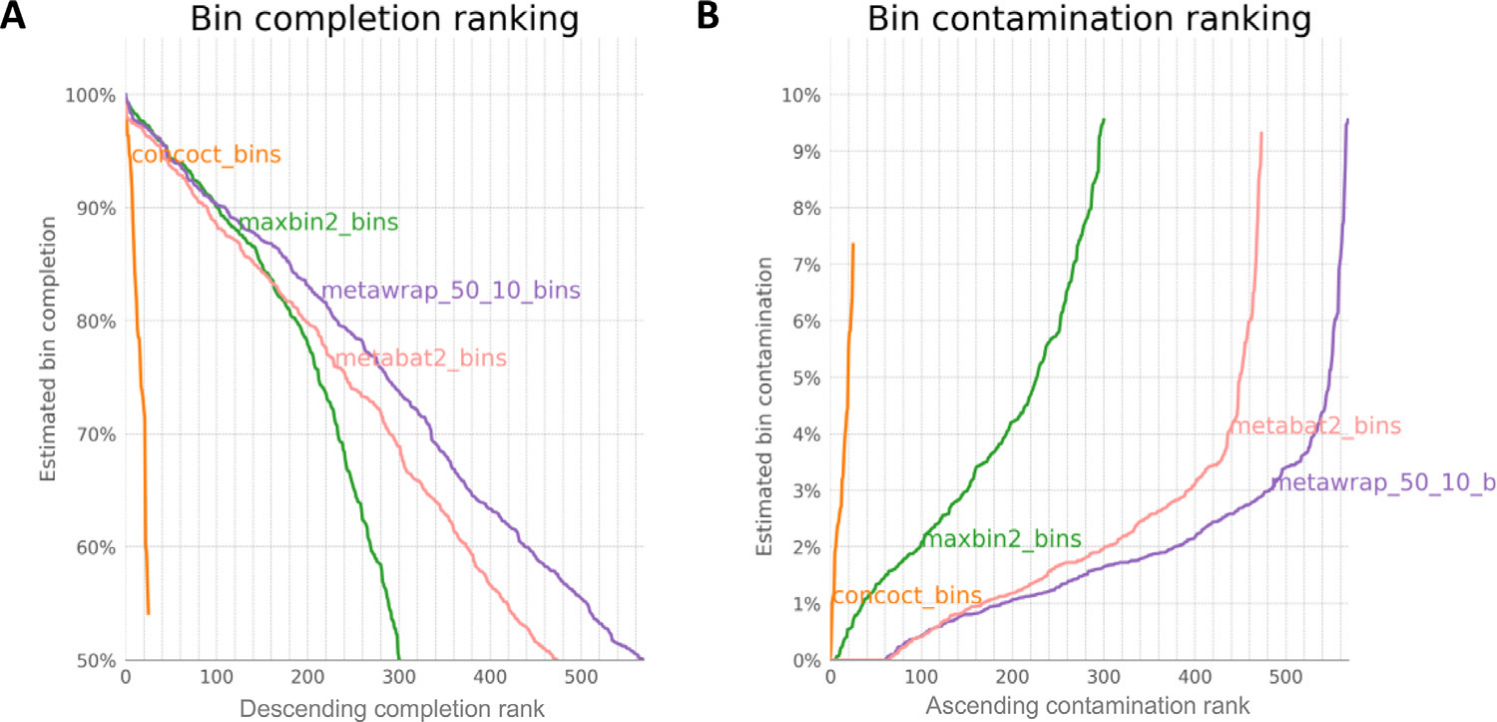
Figures A and B depict the completion and contamination, respectively, of bins initially recovered through original software (metaBAT2, MaxBin2, and CONCOCT). These bins were subsequently refined by MetaWRAP, using the criteria of ≥50 % completion and ≤10 % contamination, resulting in the final set of 569 Metagenome-Assembled Genomes (MAGs). The x-axis represents the sorted (descending) rank of MAGs in terms of a chosen metric whether completion or contamination.

**Fig. 4:**
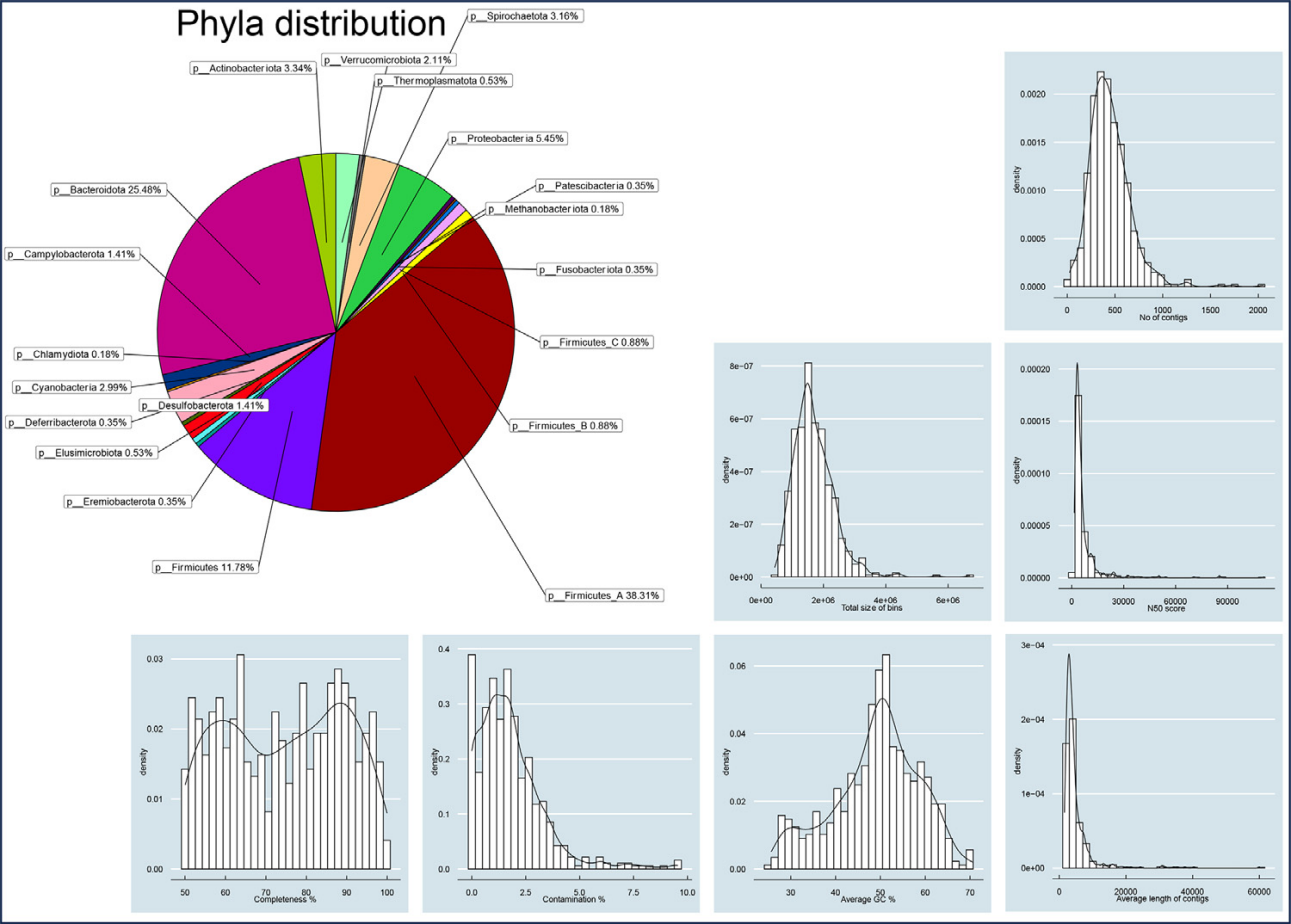
Statistics of 569 MAGs including the proportion of MAGs assigned to different phyla based on GTDB-TK taxonomy. Each panel shows density and histogram of the distribution of data over a continuous interval of a particular metric given in the Supplementary Table S2.

CheckM was applied within MetaWRAP framework on these bins [15] to assess their completion and contamination. Within MetaWRAP framework, the bins from the three binners were consolidated together (see Fig. 3) (using **metawrap bin_refinement -o BIN_REFINEMENT -A INITIAL_BINNING/metabat2_bins/ -B** **INITIAL_BINNING/maxbin2_bins/ -C INITIAL_BINNING/concoct_bins/ -c 50 -x 10**), retaining bins with ≥50 % completion and ≤10 % contamination to give a final set of 569 bins (MAGs). We obtained a mean genome completion of 74.53 % and a mean contamination of 1.77 % for bins. The summary statistics of these MAGs are given in Supplementary Table S2 with Fig. 4 showing the assignment at phyla level along with distribution of statistics.

### 4.5 Functional annotation

To derive metabolic functions, particularly nutrient cycling diagrams for carbon and sulfur, and to incorporate taxonomy using GTDB-TK [16], we employed the METABOLIC pipeline [17] (using **METABOLICC.pl -m-cutoff 0.75 -in-gn GENOMES -kofam-db small -r input_files.txt -o METABOLIC_out** where **input_files.txt** is the comma delimited path of paired-end sample reads each on a separate line and **GENOMES** is a directory containing separate fasta file of each bin). METABOLIC facilitated the recovery of protein annotations through databases such as KEGG [18], TIGRfam [19], Pfam [20], custom hidden Markov model (HMM) databases [21], dbCAN2 [22], and MEROPS [23].

### 4.6 Antibiotic resistance genes

We then employed AMRFinderPlus [8] to recover Antimicrobial Resistance (AMR) genes for the above detected bins. Since there is no real consensus on an optimal threshold for amino acids matching in the reference databases, we have employed three thresholds (from relaxed to stringent criteria) as used previously: coverage 25 %, identity 40 % [24]; coverage 50 %, identity 75 % [25]; and coverage 75 %, identity 90 % [26] (using the standard parameters along with **–ident_min X –coverage_min Y** in AMRFinderPlus, run separately for each bin). We used the same three criteria again with ABricate software (https://github.com/tseemann/abricate) to give additional annotations (using the standard parameters along with **–minid X –mincov Y and –db Z** in ABricate, run separately for each bin, and where **Z** specifies any of the databases available in ABricate).

### 4.7 Phylogenetic tree generation

To deduce the phylogeny of the MAGs, we employed GToTree [27]. The software offers various Single Copy Genes (SCGs) sets based on the resolution of domains and the taxonomic rank of interest. Specifically, we utilized two SCG sets: a 25-gene set for Bacteria and Archaea (resulting in the phylogeny recovery for 261 MAGs) and a 16-gene set (resulting in the phylogeny recovery for 232 MAGs) as proposed by [28], encompassing all major domains of life. To identify novel MAGs, we utilized the Genome Tree Toolkit available at https://github.com/donovan-h-parks/GenomeTreeTk using the command: **genometreetk pd NEWICK_TREE.nwk bin_ID.txt –per_taxa_pg_file bin_gain.txt** where **bin_ID.txt** contains the ID of a single bin, one of the leaf nodes of the **NEWICK_TREE.nwk,** and **bin_gain.txt** file is the output file containing phylogenetic gain for that particular bin. This involved assessing the phylogenetic gain for each MAG against the rest of the tree, with higher values potentially indicating novel species, and is used previously in [29]. We calculated these values for each MAG in the trees recovered using both the 25-gene Bacteria and Archaea SCGs (using **GToTree -f all_genomes.txt -H Bacteria_and_Archaea**) and the 16-gene SCGs from [28] (using **GToTree -f all_genomes.txt -H /PATHTO/Universal_Hug_et_al.hmm -o Universal_Hug_et_al**), respectively, where **all_genomes.txt** contains the path to fasta file of all bins each in a separate line. Some of the statistics were obtained from the MetaWRAP and METABOLIC software, whilst for others, custom bash scripts were written.

## Data Accessibility

The FASTA files, tables, annotations, and visualisations are provided at Figshare: http://dx.doi.org/10.6084/m9.figshare.24901884. The raw per sample sequencing data is available from the corresponding authors upon request.

## Ethics Statement

This study was approved by the Ethics Review Board (ERB) at COMSATS University Islamabad (ERB No. CUI/Bio/ERB-4-21/17/).

## CRediT authorship contribution statement

**Farrukh Saleem:** Conceptualization, Methodology, Validation, Formal analysis, Investigation, Data curation, Writing – original draft, Visualization. **Aqsa Ameer:** Conceptualization, Methodology, Validation, Formal analysis, Investigation, Data curation, Writing – original draft, Visualization. **Banaz Star-Shirko:** Methodology, Formal analysis, Writing – review & editing, Data curation. **Ciara Keating:** Funding acquisition, Resources, Writing – review & editing, Data curation. **Ozan Gundogdu:** Resources, Writing – review & editing, Data curation. **Umer Zeeshan Ijaz:** Software, Validation, Formal analysis, Resources, Writing – original draft, Supervision, Project administration, Funding acquisition. **Sundus Javed:** Conceptualization, Methodology, Resources, Writing – review & editing, Supervision, Funding acquisition.

## Data Availability

Dataset of 569 metagenome-assembled genomes from the caeca of multiple chicken breeds from commercial and backyard farming setups of Pakistan (Original data) (Figshare).

## Appendix Supplementary materials

Image, application 1
