# Supplementary material for: Dataset of 569 metagenome-assembled genomes from the caeca of multiple chicken breeds from commercial and backyard farming setups of Pakistan
Source: Data Brief. 2024 May 23;54:110552. doi: 10.1016/j.dib.2024.110552 (PMC11177053; doi:10.1016/j.dib.2024.110552)
Supplement: Supplementary file 1 [file mmc1.docx]

**Supplementary Table S1**: Read statistics after quality trimming. Note, the acronym in Sample ID column are as follows: B (Broiler); BA (Black Australorp); WL (White Layer); AFC (Antibiotic Free Control); CS (Control Shed); OS (Open Shed); FR (Free Range). The replicates are represented by suffix CX where X is in the range of 1 and 5.

| **Sample ID** | **Total Raw Reads** | **Total Paired-end Reads Retained after Quality Trimming** | **Total Reads Discarded (Forward + Reverse Reads)** |
| --- | --- | --- | --- |
| BA-AFC-C1 | 27382615 | 25844499 | 1144377 |
| BA-AFC-C2 | 26811016 | 25428419 | 1076569 |
| BA-AFC-C3 | 24082523 | 22702828 | 1067221 |
| BA-AFC-C4 | 27544753 | 26161815 | 1013166 |
| BA-AFC-C5 | 21669716 | 20313923 | 1087507 |
| BA-FR-C1 | 32402704 | 30291685 | 1686562 |
| BA-FR-C2 | 27341369 | 23863405 | 2719399 |
| BA-FR-C3 | 30134321 | 28418913 | 1319322 |
| BA-FR-C4 | 38012364 | 35999095 | 1475992 |
| BA-FR-C5 | 38011173 | 35918535 | 1525310 |
| BA-OS-C1 | 34720304 | 32650094 | 1618779 |
| BA-OS-C2 | 33805688 | 31375674 | 1970545 |
| BA-OS-C3 | 37649519 | 35523080 | 1649487 |
| BA-OS-C4 | 34440341 | 32215198 | 1743579 |
| BA-OS-C5 | 33649619 | 31544672 | 1679402 |
| B-CS-C1 | 33380502 | 30474229 | 2105552 |
| B-CS-C2 | 32549010 | 30217280 | 1857715 |
| B-CS-C3 | 32965909 | 30912410 | 1480091 |
| B-CS-C4 | 28004825 | 25830527 | 1719219 |
| B-CS-C5 | 30191901 | 28204388 | 1579705 |
| B-OS-C1 | 31470137 | 29686169 | 1275972 |
| B-OS-C2 | 29645708 | 27616034 | 1477319 |
| B-OS-C3 | 28812347 | 26774951 | 1620696 |
| B-OS-C4 | 28237310 | 26171528 | 1639262 |
| B-OS-C5 | 30009888 | 28852954 | 872251 |
| WL-CS-C1 | 30792154 | 28968434 | 1428785 |
| WL-CS-C2 | 30535069 | 28388426 | 1654057 |
| WL-CS-C3 | 27695367 | 26188316 | 1109011 |
| WL-CS-C4 | 31966211 | 30142938 | 1437909 |
| WL-CS-C5 | 30471032 | 28413054 | 1630745 |
| WL-OS-C1 | 33595423 | 31236897 | 1865993 |
| WL-OS-C2 | 31091565 | 29080694 | 1584197 |
| WL-OS-C3 | 31116349 | 29670171 | 1124425 |
| WL-OS-C4 | 36631098 | 34211738 | 1885511 |
| WL-OS-C5 | 31961068 | 30731502 | 873417 |
| B-AFC-C1 | 23461074 | 21981851 | 1325718 |
| B-AFC-C2 | 25727572 | 24341558 | 1135045 |
| B-AFC-C3 | 25793846 | 24059492 | 1547832 |
| B-AFC-C4 | 21907284 | 19741161 | 1863111 |
| WL-AFC-C1 | 29525024 | 27804867 | 1377119 |
| WL-AFC-C2 | 25152369 | 23252832 | 1465006 |
| WL-AFC-C3 | 21737743 | 20268431 | 1174159 |
| WL-AFC-C4 | 25897540 | 24430898 | 1069541 |
| WL-AFC-C5 | 26560052 | 25062411 | 1216626 |
| Negative-Control | 224 | 150 | 67 |

**Supplementary Table S2:** Overview of Metagenome-Assembled Genomes (MAGs) obtained through the MetaWRAP pipeline, meeting the criteria of ≥50% completion and ≤10% contamination as determined by the CheckM software. The GTDB-TK classification and the percentage gain (PG) scores for each MAG (whether included in the resulting tree) are presented for both the 25-gene Bacteria and Archaea SCGs and the 16-gene SCGs set from [1]. Highlighted rows in “Yellow” represent those bins where reasonable number of SCGs are detected, and are part of the phylogenetic trees.

| **Bins** | **No of contigs** | **Average length of contigs** | **Total length** | **N50 score** | **Average GC %** |  | **Completeness %** | **Contamination %** | **CheckM lineage** | **GTDB-TK classification** | **PG (Bacterial and Archaeal SCGs)** | **PG% (Bacterial and Archaeal SCGs)** | **PG (Universal Hug et al. 2016 SCGs)** [1] | **PG% (Universal Hug et al. 2016 SCGs)** [1] |
| --- | --- | --- | --- | --- | --- | --- | --- | --- | --- | --- | --- | --- | --- | --- |
| bin.1 | 462 | 8081.16 | 3733494 | 12173 | 46.0284 |  | 96.75 | 1.346 | Bacteroidales | d__Bacteria;p__Bacteroidota;c__Bacteroidia;o__Bacteroidales;f__Tannerellaceae;g__Parabacteroides;s__Parabacteroides johnsonii |  |  |  |  |
| bin.10 | 335 | 1744.24 | 584321 | 1825 | 35.6472 |  | 57.94 | 3.151 | Bacteria | d__Bacteria;p__Firmicutes;c__Bacilli;o__RF39;f__UBA660;g__CAG-988;s__ |  |  |  |  |
| bin.100 | 301 | 4168.5 | 1254720 | 4801 | 62.096 |  | 89.58 | 2.14 | Euryarchaeota | d__Archaea;p__Thermoplasmatota;c__Thermoplasmata;o__Methanomassiliicoccales;f__Methanomethylophilaceae;g__;s__ |  |  |  |  |
| bin.101 | 376 | 5275.26 | 1983497 | 6658 | 48.7583 |  | 76.15 | 2.144 | Lachnospiraceae | d__Bacteria;p__Firmicutes_A;c__Clostridia;o__Lachnospirales;f__Lachnospiraceae;g__Mediterraneibacter;s__ |  |  |  |  |
| bin.102 | 466 | 2769.59 | 1290631 | 2922 | 37.913 |  | 56.06 | 0 | Bacteria | d__Bacteria;p__Cyanobacteria;c__Vampirovibrionia;o__Gastranaerophilales;f__Gastranaerophilaceae;g__CAG-306;s__ |  |  |  |  |
| bin.103 | 216 | 7162.06 | 1547004 | 8915 | 50.0033 |  | 78.82 | 0.732 | Clostridiales | d__Bacteria;p__Firmicutes_A;c__Clostridia;o__Oscillospirales;f__Ruminococcaceae;g__UBA3818;s__ |  |  |  |  |
| bin.104 | 423 | 3673.22 | 1553771 | 3988 | 57.2455 |  | 63.51 | 2.754 | Bacteroidales | d__Bacteria;p__Bacteroidota;c__Bacteroidia;o__Bacteroidales;f__Bacteroidaceae;g__Paraprevotella;s__ |  |  |  |  |
| bin.105 | 639 | 3994.57 | 2552529 | 4674 | 55.3404 |  | 88.77 | 1.23 | Clostridiales | d__Bacteria;p__Firmicutes_A;c__Clostridia;o__Oscillospirales;f__Ruminococcaceae;g__Anaerotruncus;s__Anaerotruncus colihominis | 0.197233 | 0.380874 | 0.107576 | 0.288786 |
| bin.106 | 362 | 5453.72 | 1974246 | 7816 | 30.7487 |  | 97.64 | 0 | Gammaproteobacteria | d__Bacteria;p__Proteobacteria;c__Gammaproteobacteria;o__Enterobacterales;f__Succinivibrionaceae;g__Anaerobiospirillum_A;s__ | 0.079017 | 0.15259 | 0.047702 | 0.128056 |
| bin.107 | 840 | 1967.96 | 1653087 | 2047 | 47.6471 |  | 61.94 | 2.144 | Lachnospiraceae | d__Bacteria;p__Firmicutes_A;c__Clostridia;o__Lachnospirales;f__Lachnospiraceae;g__Merdimonas;s__Merdimonas faecis |  |  |  |  |
| bin.108 | 504 | 3046.1 | 1535233 | 3177 | 50.5649 |  | 63.27 | 2.9 | Clostridiales | d__Bacteria;p__Firmicutes_A;c__Clostridia;o__Lachnospirales;f__Lachnospiraceae;g__UBA7182;s__UBA7182 sp002160135 |  |  |  |  |
| bin.109 | 295 | 2896.19 | 854377 | 3084 | 29.6713 |  | 57.89 | 1.754 | Bacteria | d__Bacteria;p__Firmicutes_A;c__Clostridia;o__TANB77;f__CAG-508;g__CAG-273;s__CAG-273 sp900752335 |  |  |  |  |
| bin.11 | 430 | 3677.34 | 1581258 | 4352 | 47.9016 |  | 81.82 | 0 | Bacteria | d__Bacteria;p__Spirochaetota;c__Spirochaetia;o__Sphaerochaetales;f__Sphaerochaetaceae;g__;s__ |  |  |  |  |
| bin.110 | 862 | 2150.04 | 1853337 | 2257 | 39.9967 |  | 52.14 | 0.801 | Bacteroidales | d__Bacteria;p__Bacteroidota;c__Bacteroidia;o__Bacteroidales;f__Bacteroidaceae;g__Phocaeicola;s__ |  |  |  |  |
| bin.111 | 491 | 2590.1 | 1271737 | 3122 | 50.0576 |  | 69.14 | 1.867 | Bacteroidetes | d__Bacteria;p__Bacteroidota;c__Bacteroidia;o__Bacteroidales;f__UBA932;g__RC9;s__ | 0.055207 | 0.10661 |  |  |
| bin.112 | 402 | 3555.84 | 1429448 | 4327 | 53.6287 |  | 83.58 | 0.356 | Clostridiales | d__Bacteria;p__Firmicutes_A;c__Clostridia;o__Oscillospirales;f__CAG-382;g__UCG-010;s__UCG-010 sp900754535 | 0.149904 | 0.289478 | 0.110135 | 0.295656 |
| bin.113 | 766 | 3131.56 | 2398777 | 4117 | 58.2726 |  | 88.84 | 5.821 | Clostridiales | d__Bacteria;p__Firmicutes_A;c__Clostridia;o__Oscillospirales;f__Butyricicoccaceae;g__Butyricicoccus;s__ |  |  |  |  |
| bin.114 | 209 | 6659.92 | 1391923 | 10402 | 41.5257 |  | 96.37 | 0 | Euryarchaeota | d__Archaea;p__Thermoplasmatota;c__Thermoplasmata;o__Methanomassiliicoccales;f__Methanomassiliicoccaceae;g__Methanomassiliicoccus_A;s__ | 1.43908 | 2.778988 | 1.955136 | 5.248535 |
| bin.116 | 407 | 4591.39 | 1868694 | 5634 | 59.6204 |  | 77.21 | 0.68 | Bacteria | d__Bacteria;p__Verrucomicrobiota;c__Verrucomicrobiae;o__Verrucomicrobiales;f__Akkermansiaceae;g__Akkermansia;s__ | 0.171803 | 0.331766 | 0.129366 | 0.347282 |
| bin.117 | 969 | 2101.57 | 2036419 | 2263 | 60.5609 |  | 81.08 | 2.586 | Deltaproteobacteria | d__Bacteria;p__Desulfobacterota;c__Desulfovibrionia;o__Desulfovibrionales;f__Desulfovibrionaceae;g__Desulfovibrio;s__ |  |  |  |  |
| bin.118 | 668 | 1926.11 | 1286641 | 1993 | 52.5972 |  | 67.29 | 1.745 | Clostridiales | d__Bacteria;p__Firmicutes_A;c__Clostridia_A;o__Christensenellales;f__DTU072;g__;s__ | 0.057752 | 0.111525 | 0.04829 | 0.129633 |
| bin.12 | 726 | 1836.5 | 1333299 | 1857 | 51.6435 |  | 56.61 | 0.81 | Bacteria | d__Bacteria;p__Verrucomicrobiota;c__Verrucomicrobiae;o__Opitutales;f__CAG-312;g__CAG-312;s__ |  |  |  |  |
| bin.121 | 410 | 3555.94 | 1457936 | 3854 | 53.9011 |  | 51.04 | 1.759 | Bacteroidales | d__Bacteria;p__Bacteroidota;c__Bacteroidia;o__Bacteroidales;f__Bacteroidaceae;g__Prevotella;s__ |  |  |  |  |
| bin.122 | 647 | 3570.04 | 2309815 | 4152 | 61.5627 |  | 86.94 | 1.103 | Deltaproteobacteria | d__Bacteria;p__Desulfobacterota;c__Desulfovibrionia;o__Desulfovibrionales;f__Desulfovibrionaceae;g__Desulfovibrio;s__ | 0.049699 | 0.095973 | 0.033657 | 0.090352 |
| bin.123 | 400 | 4497.39 | 1798957 | 5435 | 51.4119 |  | 82.29 | 1.949 | Bacteroidetes | d__Bacteria;p__Bacteroidota;c__Bacteroidia;o__Bacteroidales;f__Rikenellaceae;g__Alistipes_A;s__Alistipes_A sp900546005 |  |  |  |  |
| bin.124 | 837 | 2606.9 | 2181976 | 3004 | 55.3593 |  | 61.72 | 1.754 | Bacteria | d__Bacteria;p__Actinobacteriota;c__Actinomycetia;o__Mycobacteriales;f__Mycobacteriaceae;g__Corynebacterium;s__Corynebacterium ammoniagenes |  |  |  |  |
| bin.125 | 571 | 1925.54 | 1099484 | 2046 | 54.0798 |  | 63.91 | 1.886 | Bacteria | d__Bacteria;p__Firmicutes;c__Bacilli;o__Erysipelotrichales;f__Erysipelotrichaceae;g__Merdibacter;s__ | 0.021405 | 0.041334 | 0.016381 | 0.043974 |
| bin.126 | 520 | 4507.42 | 2343860 | 5705 | 69.7645 |  | 85.62 | 0.595 | Actinomycetales | d__Bacteria;p__Actinobacteriota;c__Actinomycetia;o__Mycobacteriales;f__Mycobacteriaceae;g__Corynebacterium;s__Corynebacterium xerosis | 0.157494 | 0.304134 | 0.22974 | 0.616733 |
| bin.127 | 671 | 2048.56 | 1374582 | 2211 | 42.7189 |  | 51.17 | 2.371 | Bacteroidales | d__Bacteria;p__Bacteroidota;c__Bacteroidia;o__Bacteroidales;f__Tannerellaceae;g__Parabacteroides;s__Parabacteroides sp000436495 |  |  |  |  |
| bin.128 | 573 | 3687.75 | 2113083 | 4229 | 43.6003 |  | 73.01 | 1.57 | Bacteroidales | d__Bacteria;p__Bacteroidota;c__Bacteroidia;o__Bacteroidales;f__Tannerellaceae;g__Parabacteroides;s__ |  |  |  |  |
| bin.129 | 861 | 2895.61 | 2493120 | 3620 | 46.7466 |  | 86.83 | 2.558 | Bacteroidales | d__Bacteria;p__Bacteroidota;c__Bacteroidia;o__Bacteroidales;f__Muribaculaceae;g__CAG-279;s__CAG-279 sp900544305 | 0.022136 | 0.042746 | 0.025557 | 0.068608 |
| bin.13 | 289 | 2698.58 | 779891 | 2758 | 55.7414 |  | 50.47 | 0 | Bacteria | d__Bacteria;p__Firmicutes_A;c__Clostridia_A;o__Christensenellales;f__CAG-552;g__UMGS1880;s__UMGS1880 sp900555875 |  |  |  |  |
| bin.131 | 304 | 2584.18 | 785591 | 2608 | 45.2595 |  | 50.18 | 0.402 | Campylobacterales | d__Bacteria;p__Campylobacterota;c__Campylobacteria;o__Campylobacterales;f__Helicobacteraceae;g__Helicobacter_F;s__ |  |  |  |  |
| bin.132 | 356 | 6081.2 | 2164907 | 8817 | 41.1162 |  | 86.48 | 3.418 | Bacteria | d__Bacteria;p__Verrucomicrobiota;c__Verrucomicrobiae;o__Pedosphaerales;f__UBA1412;g__UBA1412;s__ | 0.429473 | 0.829349 | 0.406435 | 1.091069 |
| bin.134 | 386 | 4165.54 | 1607899 | 4870 | 49.027 |  | 73.94 | 0 | Bacteria | d__Bacteria;p__Spirochaetota;c__Spirochaetia;o__Sphaerochaetales;f__Sphaerochaetaceae;g__;s__ |  |  |  |  |
| bin.135 | 1139 | 2515.62 | 2865296 | 2947 | 56.5866 |  | 61.81 | 2.737 | Bacteroidales | d__Bacteria;p__Bacteroidota;c__Bacteroidia;o__Bacteroidales;f__Bacteroidaceae;g__CAG-617;s__ |  |  |  |  |
| bin.136 | 234 | 7302.12 | 1708697 | 10895 | 43.8731 |  | 97.16 | 0.943 | Bacteria | d__Bacteria;p__Firmicutes;c__Bacilli;o__Erysipelotrichales;f__Erysipelotrichaceae;g__Massilicoli;s__Massilicoli timonensis | 0.164225 | 0.317133 | 0.056281 | 0.151086 |
| bin.137 | 366 | 7679.46 | 2810682 | 14504 | 35.087 |  | 98.27 | 0.574 | Bacilli | d__Bacteria;p__Firmicutes;c__Bacilli;o__Bacillales;f__;g__;s__ | 0.192905 | 0.372516 | 0.129202 | 0.346842 |
| bin.139 | 356 | 4106.53 | 1461925 | 5020 | 68.3576 |  | 85.57 | 0.721 | Firmicutes | d__Bacteria;p__Firmicutes_A;c__Clostridia_A;o__Christensenellales;f__CAG-138;g__UMGS1241;s__UMGS1241 sp900550525 |  |  |  |  |
| bin.14 | 611 | 3342.27 | 2042124 | 3695 | 49.7743 |  | 72.86 | 3.375 | Clostridiales | d__Bacteria;p__Firmicutes_A;c__Clostridia;o__Lachnospirales;f__Lachnospiraceae;g__CHKCI001;s__ |  |  |  |  |
| bin.140 | 676 | 6529.38 | 4413859 | 9151 | 42.9182 |  | 66.17 | 1.724 | Bacteria | d__Bacteria;p__Bacteroidota;c__Bacteroidia;o__Bacteroidales;f__Bacteroidaceae;g__Bacteroides;s__Bacteroides fragilis |  |  |  |  |
| bin.141 | 740 | 2485.68 | 1839402 | 2950 | 51.0476 |  | 78.4 | 2.742 | Clostridiales | d__Bacteria;p__Firmicutes_A;c__Clostridia;o__Oscillospirales;f__Ruminococcaceae;g__Ruthenibacterium;s__Ruthenibacterium sp002315015 | 0.236645 | 0.456982 | 0.078284 | 0.210152 |
| bin.142 | 326 | 3137.1 | 1022694 | 3279 | 51.8982 |  | 58.28 | 0.033 | Firmicutes | d__Bacteria;p__Firmicutes_A;c__Clostridia_A;o__Christensenellales;f__Borkfalkiaceae;g__UBA11940;s__ | 0.022731 | 0.043895 |  |  |
| bin.143 | 472 | 3201.59 | 1511151 | 3507 | 63.9128 |  | 54.09 | 1.785 | Bacteria | d__Bacteria;p__Firmicutes_A;c__Clostridia;o__Oscillospirales;f__Ruminococcaceae;g__Gemmiger;s__ |  |  |  |  |
| bin.144 | 386 | 5955.35 | 2298767 | 7708 | 60.1096 |  | 91.1 | 2.419 | Clostridia | d__Bacteria;p__Firmicutes_A;c__Clostridia_A;o__Christensenellales;f__UMGS416;g__;s__ | 0.231608 | 0.447255 | 0.212155 | 0.569527 |
| bin.145 | 292 | 5597.01 | 1634327 | 6755 | 54.1188 |  | 90.2 | 3.691 | Clostridiales | d__Bacteria;p__Firmicutes_A;c__Clostridia;o__Oscillospirales;f__Acutalibacteraceae;g__UMGS1071;s__ | 0.109853 | 0.212136 | 0.064557 | 0.173303 |
| bin.146 | 652 | 3277.31 | 2136805 | 3556 | 58.1315 |  | 64.42 | 3.448 | Bacteria | d__Bacteria;p__Bacteroidota;c__Bacteroidia;o__Bacteroidales;f__Bacteroidaceae;g__Paraprevotella;s__ |  |  |  |  |
| bin.147 | 482 | 2152.84 | 1037668 | 2304 | 53.9689 |  | 65.16 | 1.102 | Clostridia | d__Bacteria;p__Firmicutes_A;c__Clostridia_A;o__Christensenellales;f__Borkfalkiaceae;g__UBA11940;s__ | 0.055621 | 0.10741 | 0.036243 | 0.097293 |
| bin.148 | 414 | 3671.57 | 1520031 | 4070 | 49.7821 |  | 53.21 | 0 | Bacteria | d__Bacteria;p__Firmicutes_A;c__Clostridia;o__Lachnospirales;f__Lachnospiraceae;g__Mediterraneibacter;s__ |  |  |  |  |
| bin.149 | 275 | 6222.97 | 1711317 | 8859 | 40.9153 |  | 91.66 | 0.766 | Bacilli | d__Bacteria;p__Firmicutes;c__Bacilli;o__Staphylococcales;f__Salinicoccaceae;g__Jeotgalicoccus;s__Jeotgalicoccus aerolatus | 0.15517 | 0.299648 | 0.124084 | 0.333101 |
| bin.15 | 183 | 3407.4 | 623554 | 3634 | 45.9641 |  | 64.37 | 0 | Bacteria | d__Bacteria;p__Proteobacteria;c__Alphaproteobacteria;o__Rs-D84;f__Rs-D84;g__Rs-D84;s__ |  |  |  |  |
| bin.151 | 458 | 3203.58 | 1467238 | 3421 | 27.3937 |  | 80.63 | 0.915 | Clostridiales | d__Bacteria;p__Firmicutes_A;c__Clostridia;o__Peptostreptococcales;f__Peptostreptococcaceae;g__Romboutsia;s__ | 0.261219 | 0.504437 | 0.16106 | 0.432364 |
| bin.152 | 539 | 3760.37 | 2026839 | 4349 | 46.5107 |  | 60.51 | 0 | Bacteria | d__Bacteria;p__Bacteroidota;c__Bacteroidia;o__Bacteroidales;f__Bacteroidaceae;g__Phocaeicola;s__Phocaeicola coprophilus |  |  |  |  |
| bin.153 | 47 | 40392.8 | 1898461 | 85373 | 42.7641 |  | 97.84 | 0.537 | Bacteria | d__Bacteria;p__Bacteroidota;c__Bacteroidia;o__Bacteroidales;f__P3;g__;s__ | 0.179894 | 0.34739 | 0.133107 | 0.357324 |
| bin.154 | 385 | 4213.02 | 1622013 | 5083 | 33.8236 |  | 81.96 | 5.128 | Bacteria | d__Bacteria;p__Cyanobacteria;c__Vampirovibrionia;o__Gastranaerophilales;f__Gastranaerophilaceae;g__;s__ | 0.156987 | 0.303155 | 0.039505 | 0.10605 |
| bin.155 | 588 | 1920.43 | 1129210 | 2019 | 36.1217 |  | 50.1 | 3.448 | Bacteria | d__Bacteria;p__Cyanobacteria;c__Vampirovibrionia;o__Gastranaerophilales;f__RUG14156;g__;s__ |  |  |  |  |
| bin.156 | 345 | 3394.3 | 1171035 | 3902 | 51.1196 |  | 70.82 | 1.241 | Clostridiales | d__Bacteria;p__Firmicutes_A;c__Clostridia_A;o__Christensenellales;f__UBA3700;g__CABKMX01;s__ |  |  |  |  |
| bin.157 | 299 | 3884.95 | 1161599 | 4579 | 48.92 |  | 68.22 | 1.614 | Proteobacteria | d__Bacteria;p__Proteobacteria;c__Gammaproteobacteria;o__Burkholderiales;f__Burkholderiaceae;g__CAG-521;s__CAG-521 sp002329575 |  |  |  |  |
| bin.158 | 304 | 3908.68 | 1188240 | 4549 | 46.2484 |  | 73.28 | 0 | Bacteria | d__Bacteria;p__Elusimicrobiota;c__Elusimicrobia;o__Elusimicrobiales;f__Elusimicrobiaceae;g__UBA1174;s__ |  |  |  |  |
| bin.159 | 101 | 7889.84 | 796874 | 12288 | 41.4037 |  | 89.01 | 0 | Bacteria | d__Bacteria;p__Proteobacteria;c__Alphaproteobacteria;o__Rs-D84;f__Rs-D84;g__Rs-D84;s__ | 0.029027 | 0.056054 | 0.021146 | 0.056767 |
| bin.16 | 384 | 3193.12 | 1226157 | 4066 | 39.9804 |  | 93.33 | 3.428 | Bacteria | d__Bacteria;p__Firmicutes;c__Bacilli;o__Erysipelotrichales;f__Erysipelotrichaceae;g__;s__ | 0.067223 | 0.129814 | 0.101005 | 0.271145 |
| bin.160 | 281 | 5106.5 | 1434927 | 6456 | 58.4441 |  | 79.08 | 1.761 | Bacteroidetes | d__Bacteria;p__Bacteroidota;c__Bacteroidia;o__Bacteroidales;f__UBA932;g__RC9;s__RC9 sp001915575 | 0.184392 | 0.356077 | 0.13773 | 0.369735 |
| bin.161 | 368 | 5485.08 | 2018511 | 7043 | 57.5532 |  | 93.43 | 2.676 | Bacteroidetes | d__Bacteria;p__Bacteroidota;c__Bacteroidia;o__Bacteroidales;f__Rikenellaceae;g__Alistipes;s__ | 0.074531 | 0.143926 | 0.101095 | 0.271388 |
| bin.162 | 1720 | 1612.76 | 2773942 | 1567 | 65.1417 |  | 50.16 | 7.684 | Actinomycetales | d__Bacteria;p__Actinobacteriota;c__Actinomycetia;o__Actinomycetales;f__Brevibacteriaceae;g__Brevibacterium;s__ |  |  |  |  |
| bin.163 | 582 | 2258.09 | 1314208 | 2566 | 52.177 |  | 75.94 | 5.437 | Clostridiales | d__Bacteria;p__Firmicutes_A;c__Clostridia;o__Peptostreptococcales;f__Anaerovoracaceae;g__UBA1191;s__ |  |  |  |  |
| bin.164 | 981 | 2299.09 | 2255409 | 2580 | 60.8621 |  | 51.22 | 7.017 | Bacteria | d__Bacteria;p__Firmicutes_A;c__Clostridia_A;o__Christensenellales;f__CAG-74;g__UMGS1600;s__ |  |  |  |  |
| bin.165 | 671 | 2345.87 | 1574077 | 2593 | 42.6764 |  | 73.48 | 2.315 | Clostridiales | d__Bacteria;p__Firmicutes_A;c__Clostridia;o__Monoglobales_A;f__UBA1381;g__12844;s__ |  |  |  |  |
| bin.166 | 607 | 2529.74 | 1535552 | 2984 | 45.6465 |  | 60.01 | 0.749 | Bacteroidales | d__Bacteria;p__Bacteroidota;c__Bacteroidia;o__Bacteroidales;f__Bacteroidaceae;g__Phocaeicola;s__Phocaeicola sp002161565 |  |  |  |  |
| bin.167 | 451 | 3988.35 | 1798747 | 4513 | 59.6408 |  | 77.62 | 0.109 | Bacteria | d__Bacteria;p__Verrucomicrobiota;c__Verrucomicrobiae;o__Verrucomicrobiales;f__Akkermansiaceae;g__Akkermansia;s__ |  |  |  |  |
| bin.169 | 588 | 2240.72 | 1317541 | 2481 | 40.0554 |  | 63.37 | 1.975 | Bacteria | d__Bacteria;p__Cyanobacteria;c__Vampirovibrionia;o__Gastranaerophilales;f__Gastranaerophilaceae;g__Gastranaerophilus;s__ | 0.074103 | 0.143099 | 0.056468 | 0.151587 |
| bin.17 | 400 | 2755.12 | 1102047 | 3409 | 29.4901 |  | 75.68 | 2.359 | Bacteria | d__Bacteria;p__Firmicutes;c__Bacilli;o__RF39;f__UBA660;g__CAG-877;s__ |  |  |  |  |
| bin.170 | 698 | 3415.76 | 2384202 | 3716 | 57.1157 |  | 63.89 | 2.469 | Bacteroidales | d__Bacteria;p__Bacteroidota;c__Bacteroidia;o__Bacteroidales;f__Bacteroidaceae;g__Prevotella;s__Prevotella sp900540415 |  |  |  |  |
| bin.171 | 255 | 3841.85 | 979673 | 4774 | 28.8636 |  | 59.96 | 1.754 | Bacteria | d__Bacteria;p__Firmicutes;c__Bacilli;o__RF39;f__UBA660;g__CAG-460;s__ |  |  |  |  |
| bin.172 | 775 | 1964.43 | 1522430 | 2054 | 59.3049 |  | 63.97 | 1.883 | Bacteroidales | d__Bacteria;p__Bacteroidota;c__Bacteroidia;o__Bacteroidales;f__Bacteroidaceae;g__CAG-617;s__ | 0.067368 | 0.130094 |  |  |
| bin.173 | 710 | 2096.09 | 1488225 | 2269 | 38.4615 |  | 51.28 | 7.758 | Bacteria | d__Bacteria;p__Firmicutes;c__Bacilli;o__Lactobacillales;f__Lactobacillaceae;g__Limosilactobacillus;s__ |  |  |  |  |
| bin.174 | 547 | 5915.12 | 3235568 | 8483 | 58.2638 |  | 91.27 | 0.816 | Gammaproteobacteria | d__Bacteria;p__Proteobacteria;c__Gammaproteobacteria;o__Enterobacterales;f__Succinivibrionaceae;g__Anaerobiospirillum;s__ |  |  |  |  |
| bin.175 | 660 | 2741.31 | 1809265 | 3306 | 30.7674 |  | 71.04 | 1.082 | Bacteria | d__Bacteria;p__Deferribacterota;c__Deferribacteres;o__Deferribacterales;f__Mucispirillaceae;g__Mucispirillum;s__ |  |  |  |  |
| bin.176 | 22 | 60173 | 1323807 | 111229 | 61.0416 |  | 97.75 | 0 | Bacteria | d__Bacteria;p__Firmicutes;c__Bacilli;o__RFN20;f__CAG-826;g__UBA4951;s__ | 0.290426 | 0.560838 | 0.368107 | 0.988177 |
| bin.177 | 650 | 2871.97 | 1866781 | 3668 | 52.581 |  | 65.01 | 0.69 | Clostridiales | d__Bacteria;p__Firmicutes_A;c__Clostridia;o__Lachnospirales;f__Lachnospiraceae;g__;s__ |  |  |  |  |
| bin.178 | 356 | 5043.31 | 1795418 | 6254 | 53.2866 |  | 51.39 | 3.508 | Bacteria | d__Bacteria;p__Firmicutes_A;c__Clostridia;o__Lachnospirales;f__Lachnospiraceae;g__GCA-900066135;s__ |  |  |  |  |
| bin.179 | 487 | 7608.86 | 3705517 | 23362 | 35.4988 |  | 99.3 | 0.335 | Clostridiales | d__Bacteria;p__Firmicutes_A;c__Clostridia;o__Oscillospirales;f__Butyricicoccaceae;g__Butyricicoccus;s__ | 0.103551 | 0.199966 | 0.083778 | 0.224902 |
| bin.18 | 572 | 2192.76 | 1254261 | 2461 | 46.6368 |  | 59.22 | 1.871 | Lachnospiraceae | d__Bacteria;p__Firmicutes_A;c__Clostridia;o__Lachnospirales;f__Lachnospiraceae;g__Mediterraneibacter;s__ |  |  |  |  |
| bin.180 | 457 | 3353.51 | 1532555 | 3661 | 53.9819 |  | 71.88 | 0.806 | Clostridia | d__Bacteria;p__Firmicutes_A;c__Clostridia_A;o__Christensenellales;f__CAG-138;g__UBA1685;s__ | 0.176083 | 0.340032 | 0.130412 | 0.35009 |
| bin.181 | 712 | 2295.13 | 1634133 | 2555 | 29.1272 |  | 78.83 | 2.579 | Bacteria | d__Bacteria;p__Firmicutes;c__Bacilli;o__Erysipelotrichales;f__Erysipelatoclostridiaceae;g__Erysipelatoclostridium;s__Erysipelatoclostridium sp002160495 | 0.041608 | 0.080349 | 0.024919 | 0.066895 |
| bin.182 | 511 | 3335.94 | 1704666 | 3980 | 54.9918 |  | 68.66 | 2.486 | Bacteroidales | d__Bacteria;p__Bacteroidota;c__Bacteroidia;o__Bacteroidales;f__Bacteroidaceae;g__Prevotella;s__ |  |  |  |  |
| bin.183 | 463 | 2339.03 | 1082973 | 2597 | 27.4247 |  | 53.38 | 1.818 | Bacteria | d__Bacteria;p__Firmicutes_A;c__Clostridia;o__TANB77;f__CAG-508;g__CAG-269;s__ |  |  |  |  |
| bin.184 | 392 | 4135.43 | 1621087 | 4816 | 53.7106 |  | 56.23 | 0 | Bacteria | d__Bacteria;p__Firmicutes_A;c__Clostridia;o__Lachnospirales;f__Lachnospiraceae;g__Enterocloster;s__ |  |  |  |  |
| bin.185 | 583 | 4047.25 | 2359546 | 6698 | 50.843 |  | 87.56 | 1.507 | Bacteroidetes | d__Bacteria;p__Bacteroidota;c__Bacteroidia;o__Bacteroidales;f__UBA932;g__RC9;s__ | 0.041097 | 0.079362 | 0.02391 | 0.064186 |
| bin.186 | 285 | 3945.4 | 1124440 | 4778 | 50.2456 |  | 58.15 | 0.671 | Clostridiales | d__Bacteria;p__Firmicutes_A;c__Clostridia;o__Lachnospirales;f__Lachnospiraceae;g__Anaerobutyricum;s__ |  |  |  |  |
| bin.187 | 625 | 2408.33 | 1505206 | 2712 | 52.4152 |  | 51.72 | 2.586 | Bacteria | d__Bacteria;p__Firmicutes_A;c__Clostridia;o__Oscillospirales;f__Acutalibacteraceae;g__;s__ |  |  |  |  |
| bin.188 | 1244 | 1893.77 | 2355855 | 1990 | 50.4496 |  | 53.53 | 1.976 | Enterobacteriaceae | d__Bacteria;p__Proteobacteria;c__Gammaproteobacteria;o__Enterobacterales;f__Enterobacteriaceae;g__Escherichia;s__Escherichia flexneri | 0.166251 | 0.321045 | 0.100321 | 0.26931 |
| bin.189 | 332 | 3229.64 | 1072241 | 3547 | 40.1103 |  | 63.12 | 2.747 | Lactobacillales | d__Bacteria;p__Firmicutes;c__Bacilli;o__Lactobacillales;f__Aerococcaceae;g__Aerococcus;s__Aerococcus urinaeequi | 0.199609 | 0.385462 | 0.134767 | 0.361779 |
| bin.19 | 279 | 8185.63 | 2283791 | 16077 | 44.3227 |  | 95.47 | 0.821 | Bacteroidales | d__Bacteria;p__Bacteroidota;c__Bacteroidia;o__Bacteroidales;f__Muribaculaceae;g__;s__ | 0.036973 | 0.071398 | 0.021406 | 0.057463 |
| bin.190 | 333 | 2948.99 | 982013 | 3053 | 61.2648 |  | 60.3 | 0 | Actinobacteria | d__Bacteria;p__Actinobacteriota;c__Coriobacteriia;o__Coriobacteriales;f__Eggerthellaceae;g__Slackia_A;s__ |  |  | 0.074086 | 0.198883 |
| bin.191 | 467 | 3750.77 | 1751610 | 4157 | 61.6121 |  | 79.27 | 2.237 | Clostridiales | d__Bacteria;p__Firmicutes_A;c__Clostridia;o__Oscillospirales;f__Oscillospiraceae;g__UBA5446;s__UBA5446 sp004553625 |  |  |  |  |
| bin.192 | 400 | 4074.9 | 1629960 | 4810 | 45.5556 |  | 76.79 | 0.828 | Lachnospiraceae | d__Bacteria;p__Firmicutes_A;c__Clostridia;o__Lachnospirales;f__Lachnospiraceae;g__Sellimonas;s__Sellimonas sp002159995 | 0.108322 | 0.209179 | 0.070572 | 0.18945 |
| bin.193 | 349 | 3706.37 | 1293523 | 4172 | 51.9087 |  | 75.47 | 2.127 | Clostridiales | d__Bacteria;p__Firmicutes_A;c__Clostridia_A;o__Christensenellales;f__Borkfalkiaceae;g__UBA11940;s__ | 0.073106 | 0.141174 |  |  |
| bin.194 | 275 | 7994.07 | 2198368 | 11116 | 57.9399 |  | 90.58 | 1.073 | Bacteroidales | d__Bacteria;p__Bacteroidota;c__Bacteroidia;o__Bacteroidales;f__Bacteroidaceae;g__Bacteroides;s__ |  |  |  |  |
| bin.195 | 604 | 3830.36 | 2313538 | 4265 | 44.9719 |  | 78.28 | 4.161 | Lactobacillales | d__Bacteria;p__Firmicutes;c__Bacilli;o__Lactobacillales;f__Lactobacillaceae;g__Lactiplantibacillus;s__Lactiplantibacillus plantarum | 0.117994 | 0.227857 | 0.086131 | 0.231218 |
| bin.196 | 990 | 2291.65 | 2268730 | 2548 | 48.4484 |  | 68.53 | 1.995 | Bacteroidales | d__Bacteria;p__Bacteroidota;c__Bacteroidia;o__Bacteroidales;f__Bacteroidaceae;g__Bacteroides;s__Bacteroides ndongoniae | 0.057697 | 0.111417 | 0.027344 | 0.073404 |
| bin.197 | 557 | 4165.29 | 2320066 | 5121 | 50.6853 |  | 86.74 | 0.791 | Clostridiales | d__Bacteria;p__Firmicutes_A;c__Clostridia;o__Lachnospirales;f__Lachnospiraceae;g__UMGS1370;s__ |  |  |  |  |
| bin.198 | 125 | 12817.6 | 1602198 | 21363 | 47.9147 |  | 87.96 | 0 | Bacteria | d__Bacteria;p__Proteobacteria;c__Alphaproteobacteria;o__RF32;f__CAG-239;g__CAG-267;s__ | 0.159971 | 0.308918 |  |  |
| bin.199 | 603 | 4124.19 | 2486887 | 5419 | 49.8592 |  | 90.11 | 2.134 | Bacteroidales | d__Bacteria;p__Bacteroidota;c__Bacteroidia;o__Bacteroidales;f__Bacteroidaceae;g__Phocaeicola;s__ |  |  |  |  |
| bin.2 | 1048 | 2548.83 | 2671179 | 3030 | 39.9017 |  | 58.42 | 0.542 | Bacteroidales | d__Bacteria;p__Bacteroidota;c__Bacteroidia;o__Bacteroidales;f__Bacteroidaceae;g__Phocaeicola;s__ |  |  |  |  |
| bin.20 | 563 | 2914.89 | 1641084 | 3048 | 58.5762 |  | 52.94 | 0.944 | Clostridiales | d__Bacteria;p__Firmicutes_A;c__Clostridia_A;o__Christensenellales;f__CAG-74;g__SFFS01;s__ |  |  |  |  |
| bin.200 | 393 | 4375.91 | 1719732 | 6561 | 53.3526 |  | 82.13 | 0.235 | Bacteria | d__Bacteria;p__Firmicutes;c__Bacilli;o__Erysipelotrichales;f__Erysipelotrichaceae;g__Merdibacter;s__Merdibacter sp900754715 |  |  |  |  |
| bin.201 | 395 | 3728.57 | 1472787 | 4245 | 40.8967 |  | 56.31 | 0.806 | Bacteria | d__Bacteria;p__Bacteroidota;c__Bacteroidia;o__Bacteroidales;f__Paludibacteraceae;g__;s__ |  |  |  |  |
| bin.202 | 384 | 3965.3 | 1522677 | 5525 | 27.1181 |  | 90.1 | 2.247 | Bacteria | d__Bacteria;p__Firmicutes;c__Bacilli;o__RF39;f__UBA660;g__UBA11963;s__ |  |  |  |  |
| bin.203 | 454 | 5187.14 | 2354960 | 6953 | 30.9574 |  | 93.71 | 2.692 | Clostridiales | d__Bacteria;p__Firmicutes_A;c__Clostridia;o__Lachnospirales;f__Anaerotignaceae;g__An114;s__An114 sp002161055 | 0.068782 | 0.132824 | 0.058898 | 0.158112 |
| bin.204 | 561 | 3461.07 | 1941660 | 3731 | 54.3157 |  | 63.79 | 0.308 | Actinomycetales | d__Bacteria;p__Actinobacteriota;c__Actinomycetia;o__Mycobacteriales;f__Mycobacteriaceae;g__Corynebacterium;s__Corynebacterium glutamicum |  |  |  |  |
| bin.205 | 346 | 4222.9 | 1461123 | 5005 | 56.0344 |  | 77.03 | 1.16 | Clostridiales | d__Bacteria;p__Firmicutes_A;c__Clostridia;o__Lachnospirales;f__Lachnospiraceae;g__;s__ |  |  |  |  |
| bin.206 | 673 | 2933.82 | 1974458 | 3495 | 47.671 |  | 81.61 | 1.139 | Clostridiales | d__Bacteria;p__Firmicutes_A;c__Clostridia;o__Oscillospirales;f__Acutalibacteraceae;g__UBA737;s__ | 0.176171 | 0.340202 |  |  |
| bin.207 | 681 | 3127.36 | 2129735 | 3414 | 45.1683 |  | 66.07 | 1.285 | Clostridiales | d__Bacteria;p__Firmicutes_A;c__Clostridia;o__Lachnospirales;f__Lachnospiraceae;g__Blautia_A;s__Blautia_A sp002159835 |  |  |  |  |
| bin.208 | 355 | 5922.01 | 2102315 | 8164 | 45.4958 |  | 53.6 | 0 | Bacteria | d__Bacteria;p__Bacteroidota;c__Bacteroidia;o__Bacteroidales;f__Bacteroidaceae;g__Paraprevotella;s__ |  |  |  |  |
| bin.209 | 526 | 3856.23 | 2028376 | 4636 | 30.285 |  | 85.66 | 1.886 | Bacteria | d__Bacteria;p__Firmicutes;c__Bacilli;o__Erysipelotrichales;f__Erysipelatoclostridiaceae;g__UBA3379;s__UBA3379 sp002359025 |  |  |  |  |
| bin.21 | 315 | 7122.1 | 2243462 | 10060 | 51.4606 |  | 86.9 | 2.046 | Lachnospiraceae | d__Bacteria;p__Firmicutes_A;c__Clostridia;o__Lachnospirales;f__Lachnospiraceae;g__Massilistercora;s__ |  |  |  |  |
| bin.210 | 487 | 4345.79 | 2116402 | 5158 | 45.6405 |  | 79.44 | 2.964 | Clostridiales | d__Bacteria;p__Firmicutes_A;c__Clostridia;o__Oscillospirales;f__Ruminococcaceae;g__Negativibacillus;s__Negativibacillus massiliensis |  |  |  |  |
| bin.211 | 184 | 9029.42 | 1661413 | 12250 | 50.871 |  | 92.61 | 1.363 | Clostridiales | d__Bacteria;p__Firmicutes_A;c__Clostridia;o__Oscillospirales;f__CAG-382;g__UMGS1307;s__UMGS1307 sp900550505 | 0.140792 | 0.271882 | 0.124099 | 0.333142 |
| bin.212 | 419 | 4479.1 | 1876741 | 5302 | 54.5935 |  | 80.26 | 0.503 | Clostridiales | d__Bacteria;p__Firmicutes_A;c__Clostridia;o__Oscillospirales;f__Acutalibacteraceae;g__Anaeromassilibacillus;s__Anaeromassilibacillus sp002159845 | 0.079032 | 0.152618 | 0.051955 | 0.139473 |
| bin.213 | 585 | 2073.68 | 1213100 | 2190 | 48.1082 |  | 58.21 | 1.136 | Bacteria | d__Bacteria;p__Spirochaetota;c__Spirochaetia;o__Sphaerochaetales;f__Sphaerochaetaceae;g__Spiro-01;s__ | 0.038129 | 0.07363 | 0.027694 | 0.074343 |
| bin.214 | 252 | 3967.43 | 999792 | 4729 | 42.5686 |  | 69.22 | 2.822 | Clostridia | d__Bacteria;p__Firmicutes_A;c__Clostridia_A;o__Christensenellales;f__CAG-917;g__CAG-917;s__CAG-917 sp000437555 |  |  |  |  |
| bin.215 | 397 | 4245.6 | 1685502 | 5743 | 49.2588 |  | 92.37 | 1.075 | Bacteria | d__Bacteria;p__Proteobacteria;c__Alphaproteobacteria;o__RF32;f__CAG-239;g__CAG-495;s__CAG-495 sp000436375 | 0.16371 | 0.316137 | 0.196936 | 0.528672 |
| bin.216 | 121 | 18257.1 | 2209107 | 32306 | 61.6294 |  | 97.76 | 0 | Clostridiales | d__Bacteria;p__Firmicutes_A;c__Clostridia;o__Oscillospirales;f__Butyricicoccaceae;g__Butyricicoccus_A;s__ | 0.105077 | 0.202912 | 0.085536 | 0.229621 |
| bin.217 | 436 | 2066.45 | 900971 | 2178 | 39.3227 |  | 51.42 | 1.196 | Bacteria | d__Bacteria;p__Cyanobacteria;c__Vampirovibrionia;o__Gastranaerophilales;f__Gastranaerophilaceae;g__;s__ |  |  |  |  |
| bin.218 | 232 | 5161.74 | 1197524 | 6401 | 25.6289 |  | 88.6 | 2.787 | Bacteria | d__Bacteria;p__Firmicutes;c__Bacilli;o__RF39;f__UBA660;g__RUG591;s__ | 0.222583 | 0.429826 | 0.151205 | 0.405907 |
| bin.219 | 265 | 6951.33 | 1842103 | 13127 | 49.9463 |  | 93.88 | 1.19 | Bacteroidetes | d__Bacteria;p__Bacteroidota;c__Bacteroidia;o__Bacteroidales;f__UBA932;g__CAG-831;s__ | 0.111775 | 0.215847 | 0.065796 | 0.176627 |
| bin.22 | 241 | 5797.79 | 1397267 | 7168 | 46.0008 |  | 81.6 | 1.285 | Bacteroidetes | d__Bacteria;p__Bacteroidota;c__Bacteroidia;o__Bacteroidales;f__UBA932;g__UBA1232;s__ | 0.102229 | 0.197414 | 0.064272 | 0.172537 |
| bin.220 | 429 | 2915.17 | 1250610 | 3076 | 33.0082 |  | 68.44 | 2.564 | Bacteria | d__Bacteria;p__Cyanobacteria;c__Vampirovibrionia;o__Gastranaerophilales;f__Gastranaerophilaceae;g__;s__ |  |  | 0.031815 | 0.085407 |
| bin.221 | 151 | 10475.6 | 1581822 | 14964 | 40.4796 |  | 96.27 | 0.543 | Lactobacillales | d__Bacteria;p__Firmicutes;c__Bacilli;o__Lactobacillales;f__Lactobacillaceae;g__Limosilactobacillus;s__Limosilactobacillus vaginalis | 0.042309 | 0.081702 |  |  |
| bin.222 | 1580 | 2663.83 | 4208855 | 3132 | 43.6117 |  | 73.01 | 3.292 | Bacteria | d__Bacteria;p__Bacteroidota;c__Bacteroidia;o__Bacteroidales;f__Marinifilaceae;g__Butyricimonas;s__Butyricimonas paravirosa | 0.0459 | 0.088638 | 0.026352 | 0.070742 |
| bin.223 | 720 | 3031.11 | 2182400 | 3752 | 61.4511 |  | 87.01 | 2.918 | Clostridiales | d__Bacteria;p__Firmicutes_A;c__Clostridia;o__Oscillospirales;f__Ruminococcaceae;g__Gemmiger;s__ |  |  |  |  |
| bin.224 | 446 | 2663.89 | 1188097 | 3184 | 38.7817 |  | 84.61 | 1.426 | Lactobacillales | d__Bacteria;p__Firmicutes;c__Bacilli;o__Lactobacillales;f__Lactobacillaceae;g__Limosilactobacillus;s__Limosilactobacillus reuteri_E | 0.020837 | 0.040237 | 0.044273 | 0.118851 |
| bin.225 | 742 | 2534.34 | 1880482 | 2920 | 63.4211 |  | 84.17 | 2.908 | Clostridiales | d__Bacteria;p__Firmicutes_A;c__Clostridia;o__Oscillospirales;f__Oscillospiraceae;g__UBA5446;s__UBA5446 sp900544295 |  |  |  |  |
| bin.226 | 283 | 4002.1 | 1132594 | 4827 | 61.2808 |  | 57.89 | 2.631 | Bacteria | d__Bacteria;p__Actinobacteriota;c__Coriobacteriia;o__Coriobacteriales;f__Eggerthellaceae;g__UMGS1293;s__ |  |  |  |  |
| bin.227 | 378 | 3780.76 | 1429126 | 4289 | 47.7755 |  | 83.98 | 2.647 | Campylobacterales | d__Bacteria;p__Campylobacterota;c__Campylobacteria;o__Campylobacterales;f__Helicobacteraceae;g__Helicobacter_F;s__ | 0.148621 | 0.286999 | 0.092794 | 0.249105 |
| bin.228 | 201 | 15996.7 | 3215346 | 24186 | 46.0145 |  | 89.11 | 0.418 | Bacteroidales | d__Bacteria;p__Bacteroidota;c__Bacteroidia;o__Bacteroidales;f__Bacteroidaceae;g__Bacteroides;s__Bacteroides clarus |  |  |  |  |
| bin.229 | 346 | 4445.03 | 1537979 | 5458 | 65.2305 |  | 57.24 | 1.724 | Bacteria | d__Bacteria;p__Actinobacteriota;c__Coriobacteriia;o__Coriobacteriales;f__Coriobacteriaceae;g__Collinsella;s__ |  |  |  |  |
| bin.23 | 301 | 3593.22 | 1081558 | 4445 | 50.334 |  | 63.39 | 1.724 | Bacteria | d__Bacteria;p__Firmicutes_A;c__Clostridia;o__Lachnospirales;f__Lachnospiraceae;g__;s__ |  |  |  |  |
| bin.230 | 582 | 3268.15 | 1902062 | 3540 | 44.6389 |  | 72.46 | 2.237 | Clostridiales | d__Bacteria;p__Firmicutes_A;c__Clostridia;o__Lachnospirales;f__Anaerotignaceae;g__Anaerotignum;s__ |  |  |  |  |
| bin.231 | 478 | 6121.85 | 2926246 | 8359 | 51.2759 |  | 88.13 | 2.8 | Bacteroidales | d__Bacteria;p__Bacteroidota;c__Bacteroidia;o__Bacteroidales;f__Bacteroidaceae;g__Bacteroides;s__ |  |  |  |  |
| bin.232 | 502 | 5858.24 | 2940836 | 8980 | 52.075 |  | 90.19 | 4.43 | Clostridiales | d__Bacteria;p__Firmicutes_A;c__Clostridia;o__Lachnospirales;f__Lachnospiraceae;g__Enterocloster;s__Enterocloster sp900547035 |  |  |  |  |
| bin.233 | 325 | 4295.62 | 1396075 | 4989 | 67.0062 |  | 58.93 | 0 | Bacteria | d__Bacteria;p__Firmicutes_A;c__Clostridia_A;o__Christensenellales;f__CAG-74;g__;s__ |  |  |  |  |
| bin.234 | 205 | 6198.18 | 1270626 | 8440 | 41.9237 |  | 81.26 | 3.38 | Bacteria | d__Bacteria;p__Firmicutes;c__Bacilli;o__Erysipelotrichales;f__Erysipelotrichaceae;g__Faecalicoccus;s__ |  |  |  |  |
| bin.235 | 356 | 2231.42 | 794386 | 2553 | 32.5316 |  | 68.42 | 2.62 | Lactobacillales | d__Bacteria;p__Firmicutes;c__Bacilli;o__Lactobacillales;f__Lactobacillaceae;g__Ligilactobacillus;s__Ligilactobacillus salivarius | 0.049826 | 0.096219 | 0.028554 | 0.076654 |
| bin.236 | 311 | 4258.57 | 1324416 | 5016 | 59.4135 |  | 71.69 | 1.612 | Clostridia | d__Bacteria;p__Firmicutes_A;c__Clostridia_A;o__Christensenellales;f__Borkfalkiaceae;g__UMGS1004;s__ |  |  |  |  |
| bin.237 | 332 | 2544.6 | 844806 | 2570 | 31.3161 |  | 53.21 | 3.448 | Bacteria | d__Bacteria;p__Fusobacteriota;c__Fusobacteriia;o__Fusobacteriales;f__Fusobacteriaceae;g__Fusobacterium_A;s__Fusobacterium_A sp900549465 |  |  |  |  |
| bin.238 | 455 | 2044.15 | 930088 | 2176 | 41.593 |  | 63.06 | 1.129 | Lactobacillales | d__Bacteria;p__Firmicutes;c__Bacilli;o__Lactobacillales;f__Lactobacillaceae;g__Ligilactobacillus;s__Ligilactobacillus agilis | 0.046419 | 0.08964 | 0.035499 | 0.095296 |
| bin.24 | 157 | 15770.9 | 2476024 | 23984 | 46.9926 |  | 96.39 | 0 | Bacteroidales | d__Bacteria;p__Bacteroidota;c__Bacteroidia;o__Bacteroidales;f__Muribaculaceae;g__CAG-279;s__ | 0.022022 | 0.042527 | 0.028178 | 0.075643 |
| bin.240 | 273 | 9962.22 | 2719687 | 18099 | 61.3488 |  | 93.88 | 3.659 | Bacteroidetes | d__Bacteria;p__Bacteroidota;c__Bacteroidia;o__Bacteroidales;f__Rikenellaceae;g__Alistipes;s__Alistipes dispar |  |  |  |  |
| bin.241 | 340 | 4184.18 | 1422621 | 5334 | 49.9451 |  | 77.12 | 1.685 | Bacteria | d__Bacteria;p__Elusimicrobiota;c__Elusimicrobia;o__Elusimicrobiales;f__Elusimicrobiaceae;g__CADBRU01;s__ |  |  |  |  |
| bin.242 | 312 | 2657.94 | 829278 | 2797 | 28.3389 |  | 55.35 | 0.398 | Clostridiales | d__Bacteria;p__Firmicutes_A;c__Clostridia;o__TANB77;f__CAG-508;g__UMGS1781;s__ | 0.106327 | 0.205326 | 0.085997 | 0.230859 |
| bin.243 | 646 | 5038.56 | 3254911 | 6540 | 33.5676 |  | 94.27 | 1.067 | Clostridiales | d__Bacteria;p__Firmicutes_A;c__Clostridia;o__Lachnospirales;f__Anaerotignaceae;g__ASF356;s__ | 0.07883 | 0.152228 | 0.051283 | 0.137669 |
| bin.244 | 758 | 2383.58 | 1806754 | 2661 | 46.6976 |  | 78.43 | 2.387 | Lachnospiraceae | d__Bacteria;p__Firmicutes_A;c__Clostridia;o__Lachnospirales;f__Lachnospiraceae;g__Sellimonas;s__Sellimonas sp002161525 |  |  |  |  |
| bin.245 | 281 | 3567.34 | 1002422 | 4055 | 35.2211 |  | 83.14 | 4.206 | Bacteria | d__Bacteria;p__Firmicutes;c__Bacilli;o__RF39;f__UBA660;g__CAG-988;s__CAG-988 sp003149915 | 0.170873 | 0.329971 | 0.150891 | 0.405064 |
| bin.246 | 314 | 4755.71 | 1493294 | 6050 | 55.7079 |  | 62.29 | 0.442 | Clostridia | d__Bacteria;p__Firmicutes_B;c__Dehalobacteriia;o__UBA4068;f__UBA4068;g__;s__ | 0.100165 | 0.193427 | 0.067943 | 0.182393 |
| bin.247 | 145 | 10373.5 | 1504152 | 23438 | 35.3035 |  | 98.92 | 1.604 | Lactobacillales | d__Bacteria;p__Firmicutes;c__Bacilli;o__Lactobacillales;f__Lactobacillaceae;g__Ligilactobacillus;s__ | 0.077706 | 0.150057 | 0.047608 | 0.127804 |
| bin.248 | 424 | 4177.19 | 1771129 | 5230 | 50.5482 |  | 87.12 | 2.442 | Bacteria | d__Bacteria;p__Spirochaetota;c__Spirochaetia;o__Sphaerochaetales;f__Sphaerochaetaceae;g__;s__ | 0.03917 | 0.075641 | 0.028964 | 0.077753 |
| bin.249 | 504 | 2330.37 | 1174507 | 2570 | 49.0284 |  | 78.97 | 3.615 | Bacteria | d__Bacteria;p__Spirochaetota;c__Spirochaetia;o__Sphaerochaetales;f__Sphaerochaetaceae;g__;s__ | 0.039373 | 0.076032 | 0.021145 | 0.056762 |
| bin.25 | 467 | 2115.55 | 987963 | 2313 | 51.2859 |  | 72.1 | 3.761 | Bacteria | d__Bacteria;p__Firmicutes_A;c__Clostridia;o__Peptostreptococcales;f__Anaerovoracaceae;g__UBA1191;s__ | 0.052534 | 0.101448 | 0.033499 | 0.089928 |
| bin.250 | 309 | 4127.79 | 1275487 | 4946 | 29.3516 |  | 59.63 | 0.934 | Archaea | d__Archaea;p__Methanobacteriota;c__Methanobacteria;o__Methanobacteriales;f__Methanobacteriaceae;g__Methanobrevibacter_A;s__ |  |  |  |  |
| bin.251 | 428 | 2767.55 | 1184510 | 3153 | 56.1697 |  | 60.15 | 1.342 | Clostridiales | d__Bacteria;p__Firmicutes_A;c__Clostridia;o__Oscillospirales;f__Oscillospiraceae;g__CAG-110;s__ |  |  |  |  |
| bin.252 | 297 | 8764.96 | 2603194 | 12859 | 46.7802 |  | 96.26 | 2.988 | Lachnospiraceae | d__Bacteria;p__Firmicutes_A;c__Clostridia;o__Lachnospirales;f__Lachnospiraceae;g__Acetatifactor;s__ | 0.107345 | 0.207292 | 0.082193 | 0.220646 |
| bin.253 | 538 | 2676.99 | 1440219 | 3104 | 47.1981 |  | 82.88 | 1.898 | Clostridiales | d__Bacteria;p__Firmicutes_A;c__Clostridia;o__Lachnospirales;f__Lachnospiraceae;g__;s__ | 0.162419 | 0.313645 | 0.062509 | 0.167805 |
| bin.254 | 308 | 7043.15 | 2169290 | 9425 | 50.1969 |  | 87.36 | 1.169 | Lachnospiraceae | d__Bacteria;p__Firmicutes_A;c__Clostridia;o__Lachnospirales;f__Lachnospiraceae;g__Mediterraneibacter;s__ |  |  |  |  |
| bin.255 | 651 | 1962.3 | 1277456 | 1998 | 60.0829 |  | 56.73 | 1.578 | Clostridia | d__Bacteria;p__Firmicutes_A;c__Clostridia_A;o__Christensenellales;f__Borkfalkiaceae;g__Borkfalkia;s__ |  |  |  |  |
| bin.256 | 328 | 4271.58 | 1401077 | 5377 | 54.5675 |  | 66.91 | 6.112 | Bacteria | d__Bacteria;p__Spirochaetota;c__Spirochaetia;o__Sphaerochaetales;f__Sphaerochaetaceae;g__;s__ |  |  |  |  |
| bin.257 | 483 | 5195.96 | 2509651 | 6293 | 51.9072 |  | 59.82 | 1.724 | Bacteria | d__Bacteria;p__Firmicutes_A;c__Clostridia;o__Lachnospirales;f__Lachnospiraceae;g__Mediterraneibacter;s__ |  |  |  |  |
| bin.258 | 68 | 31689.2 | 2154863 | 46551 | 56.7104 |  | 93.91 | 0.32 | Bacteroidetes | d__Bacteria;p__Bacteroidota;c__Bacteroidia;o__Bacteroidales;f__Rikenellaceae;g__Tidjanibacter;s__Tidjanibacter inops_A | 0.023562 | 0.045501 | 0.01349 | 0.036215 |
| bin.26 | 421 | 3866.53 | 1627809 | 4435 | 55.1713 |  | 63.04 | 0 | Bacteria | d__Bacteria;p__Firmicutes_A;c__Clostridia;o__Lachnospirales;f__Lachnospiraceae;g__Eisenbergiella;s__ |  |  |  |  |
| bin.260 | 830 | 2436.1 | 2021966 | 2871 | 57.0445 |  | 74.48 | 2.52 | Deltaproteobacteria | d__Bacteria;p__Desulfobacterota;c__Desulfovibrionia;o__Desulfovibrionales;f__Desulfovibrionaceae;g__Desulfovibrio;s__Desulfovibrio sp002159665 | 0.019263 | 0.037198 | 0.121866 | 0.327149 |
| bin.262 | 530 | 2915.48 | 1545202 | 3436 | 37.877 |  | 78.4 | 3.513 | Bacteria | d__Bacteria;p__Cyanobacteria;c__Vampirovibrionia;o__Gastranaerophilales;f__Gastranaerophilaceae;g__;s__ | 0.016895 | 0.032625 |  |  |
| bin.263 | 260 | 7332.62 | 1906480 | 15355 | 38.327 |  | 96.88 | 0.234 | Campylobacterales | d__Bacteria;p__Campylobacterota;c__Campylobacteria;o__Campylobacterales;f__Helicobacteraceae;g__Helicobacter_B;s__ | 0.162102 | 0.313034 | 0.098898 | 0.265491 |
| bin.264 | 482 | 3215.93 | 1550080 | 3560 | 64.3767 |  | 73.1 | 1.209 | Actinobacteria | d__Bacteria;p__Actinobacteriota;c__Coriobacteriia;o__Coriobacteriales;f__Coriobacteriaceae;g__An2-A;s__ |  |  |  |  |
| bin.265 | 244 | 7959.52 | 1942122 | 10889 | 48.8296 |  | 90.51 | 4.391 | Bacteria | d__Bacteria;p__Verrucomicrobiota;c__Lentisphaeria;o__Victivallales;f__Victivallaceae;g__UMGS1518;s__ | 0.158187 | 0.305473 | 0.347831 | 0.933747 |
| bin.266 | 512 | 4663.31 | 2387615 | 5608 | 50.5577 |  | 78.14 | 2.531 | Clostridiales | d__Bacteria;p__Firmicutes_A;c__Clostridia;o__Lachnospirales;f__Lachnospiraceae;g__GCA-900066135;s__ |  |  |  |  |
| bin.267 | 468 | 3013.51 | 1410324 | 3246 | 36.0427 |  | 55.58 | 2.201 | Bacteria | d__Bacteria;p__Firmicutes;c__Bacilli;o__Erysipelotrichales;f__Erysipelotrichaceae;g__Faecalicoccus;s__ |  |  |  |  |
| bin.268 | 589 | 2212.7 | 1303281 | 2432 | 50.0441 |  | 61.61 | 1.428 | Bacteroidetes | d__Bacteria;p__Bacteroidota;c__Bacteroidia;o__Bacteroidales;f__UBA932;g__RC9;s__ |  |  |  |  |
| bin.269 | 530 | 2764.83 | 1465361 | 3277 | 50.7327 |  | 82.48 | 2.987 | Lactobacillus | d__Bacteria;p__Firmicutes;c__Bacilli;o__Lactobacillales;f__Lactobacillaceae;g__Lactobacillus;s__ |  |  |  |  |
| bin.27 | 357 | 3208.14 | 1145306 | 3374 | 44.1645 |  | 62.7 | 1.398 | Clostridiales | d__Bacteria;p__Firmicutes_A;c__Clostridia_A;o__Christensenellales;f__CAG-314;g__UMGS1707;s__ |  |  |  |  |
| bin.270 | 450 | 3564.95 | 1604226 | 4088 | 53.6064 |  | 74.93 | 0.754 | Bacteroidales | d__Bacteria;p__Bacteroidota;c__Bacteroidia;o__Bacteroidales;f__UBA11471;g__UBA11471;s__UBA11471 sp900542765 | 0.07818 | 0.150971 | 0.05719 | 0.153526 |
| bin.271 | 602 | 4599.35 | 2768807 | 5798 | 69.1792 |  | 78.42 | 0.588 | Actinomycetales | d__Bacteria;p__Actinobacteriota;c__Actinomycetia;o__Mycobacteriales;f__Mycobacteriaceae;g__Dietzia;s__Dietzia aerolata | 0.127913 | 0.247012 |  |  |
| bin.272 | 339 | 4529.66 | 1535554 | 5334 | 45.1089 |  | 79.04 | 1.047 | Selenomonadales | d__Bacteria;p__Firmicutes_C;c__Negativicutes;o__Veillonellales;f__Veillonellaceae;g__Veillonella_A;s__Veillonella_A magna |  |  |  |  |
| bin.273 | 346 | 5757.73 | 1992174 | 7799 | 41.1464 |  | 90.19 | 0.358 | Bacteroidetes | d__Bacteria;p__Bacteroidota;c__Bacteroidia;o__Bacteroidales;f__Rikenellaceae;g__;s__ |  |  |  |  |
| bin.274 | 423 | 2693.06 | 1139165 | 3228 | 57.8105 |  | 73.45 | 0.886 | Clostridiales | d__Bacteria;p__Firmicutes_A;c__Clostridia_A;o__Christensenellales;f__CAG-917;g__UMGS1688;s__ |  |  |  |  |
| bin.275 | 202 | 11658 | 2354906 | 24630 | 37.8363 |  | 96.23 | 0.537 | Bacteria | d__Bacteria;p__Bacteroidota;c__Bacteroidia;o__Bacteroidales;f__F082;g__F082;s__F082 sp002633315 | 0.21567 | 0.416477 | 0.161143 | 0.432586 |
| bin.276 | 323 | 2714.94 | 876925 | 2773 | 50.4469 |  | 50.52 | 2.013 | Clostridiales | d__Bacteria;p__Firmicutes_A;c__Clostridia;o__Oscillospirales;f__CAG-272;g__QALR01;s__QALR01 sp003150035 |  |  |  |  |
| bin.277 | 439 | 3564.94 | 1565007 | 3992 | 59.3 |  | 70.51 | 2.88 | Clostridiales | d__Bacteria;p__Firmicutes_A;c__Clostridia_A;o__Christensenellales;f__Borkfalkiaceae;g__Borkfalkia;s__ |  |  |  |  |
| bin.278 | 441 | 5049.14 | 2226671 | 8785 | 53.1078 |  | 83.63 | 1.124 | Bacteria | d__Bacteria;p__Bacteroidota;c__Bacteroidia;o__Bacteroidales;f__P3;g__;s__ | 0.18099 | 0.349507 | 0.143831 | 0.386113 |
| bin.279 | 476 | 3337.06 | 1588441 | 3785 | 60.7941 |  | 66.51 | 1.88 | Bacteria | d__Bacteria;p__Firmicutes_A;c__Clostridia;o__Oscillospirales;f__Oscillospiraceae;g__UBA5446;s__UBA5446 sp900544765 |  |  |  |  |
| bin.28 | 567 | 3927.25 | 2226748 | 5587 | 44.9161 |  | 92.96 | 0.061 | Clostridiales | d__Bacteria;p__Firmicutes_A;c__Clostridia;o__Lachnospirales;f__Lachnospiraceae;g__Frisingicoccus;s__Frisingicoccus sp900753685 | 0.086648 | 0.167325 | 0.080836 | 0.217002 |
| bin.280 | 444 | 4358.31 | 1935091 | 5094 | 53.6996 |  | 53.08 | 1.724 | Bacteria | d__Bacteria;p__Bacteroidota;c__Bacteroidia;o__Bacteroidales;f__Bacteroidaceae;g__Bacteroides;s__ |  |  |  |  |
| bin.281 | 769 | 2146.97 | 1651022 | 2378 | 64.2116 |  | 74.18 | 1.103 | Deltaproteobacteria | d__Bacteria;p__Desulfobacterota;c__Desulfovibrionia;o__Desulfovibrionales;f__Desulfovibrionaceae;g__Desulfovibrio;s__Desulfovibrio sp900556755 | 0.043232 | 0.083484 | 0.035231 | 0.094578 |
| bin.282 | 649 | 2375.03 | 1541392 | 2780 | 44.9799 |  | 64.92 | 0.318 | Clostridiales | d__Bacteria;p__Firmicutes_A;c__Clostridia;o__Lachnospirales;f__Lachnospiraceae;g__Blautia;s__ |  |  |  |  |
| bin.283 | 248 | 6085.52 | 1509209 | 7739 | 49.9472 |  | 81.7 | 0.95 | Clostridiales | d__Bacteria;p__Firmicutes_A;c__Clostridia_A;o__Christensenellales;f__CAG-917;g__UMGS1688;s__UMGS1688 sp900545885 | 0.02383 | 0.046018 | 0.132471 | 0.355617 |
| bin.284 | 406 | 2552.5 | 1036313 | 3054 | 59.6548 |  | 63.4 | 8.307 | Clostridiales | d__Bacteria;p__Firmicutes_A;c__Clostridia_A;o__Christensenellales;f__Borkfalkiaceae;g__UMGS1004;s__ |  |  |  |  |
| bin.285 | 530 | 3237.83 | 1716052 | 3538 | 50.2546 |  | 55.98 | 0 | Bacteria | d__Bacteria;p__Firmicutes_A;c__Clostridia;o__Oscillospirales;f__Acutalibacteraceae;g__;s__ |  |  |  |  |
| bin.286 | 522 | 1953.2 | 1019573 | 1977 | 50.8678 |  | 64.47 | 5.273 | Lactobacillales | d__Bacteria;p__Firmicutes;c__Bacilli;o__Lactobacillales;f__Lactobacillaceae;g__Limosilactobacillus;s__Limosilactobacillus ingluviei | 0.110674 | 0.21372 | 0.06234 | 0.16735 |
| bin.287 | 282 | 6145.05 | 1732903 | 8572 | 46.1289 |  | 75.13 | 1.836 | Clostridia | d__Bacteria;p__Firmicutes_B;c__Dehalobacteriia;o__UBA4068;f__UBA5755;g__;s__ | 0.226809 | 0.437989 | 0.15728 | 0.422215 |
| bin.288 | 237 | 9726.03 | 2305069 | 14436 | 63.7047 |  | 97.4 | 0.403 | Clostridia | d__Bacteria;p__Firmicutes_A;c__Clostridia_A;o__Christensenellales;f__CAG-74;g__WRGP01;s__ | 0.124641 | 0.240692 | 0.105123 | 0.282201 |
| bin.289 | 368 | 3377.46 | 1242904 | 4317 | 59.0439 |  | 85.75 | 3.391 | Clostridiales | d__Bacteria;p__Firmicutes_A;c__Clostridia_A;o__Christensenellales;f__CAG-917;g__UMGS1688;s__UMGS1688 sp900553825 |  |  |  |  |
| bin.29 | 649 | 2952.48 | 1916161 | 3818 | 47.5699 |  | 74.28 | 1.482 | Bacteroidales | d__Bacteria;p__Bacteroidota;c__Bacteroidia;o__Bacteroidales;f__Bacteroidaceae;g__Phocaeicola;s__Phocaeicola barnesiae | 0.033185 | 0.064083 | 0.017027 | 0.045709 |
| bin.290 | 227 | 4370.24 | 992045 | 5104 | 26.858 |  | 62.13 | 1.006 | Clostridiales | d__Bacteria;p__Firmicutes_A;c__Clostridia;o__TANB77;f__CAG-508;g__;s__ | 0.106734 | 0.206112 | 0.095818 | 0.257222 |
| bin.291 | 323 | 2765.11 | 893130 | 2872 | 29.1676 |  | 52.76 | 1.685 | Bacteria | d__Bacteria;p__Firmicutes;c__Bacilli;o__RF39;f__UBA660;g__CAG-877;s__ |  |  |  |  |
| bin.292 | 392 | 4227.68 | 1657250 | 5182 | 53.4516 |  | 71.33 | 1.476 | Clostridiales | d__Bacteria;p__Firmicutes_A;c__Clostridia;o__Lachnospirales;f__Lachnospiraceae;g__;s__ |  |  |  |  |
| bin.293 | 411 | 5108.19 | 2099467 | 6798 | 60.5729 |  | 83.05 | 1.765 | Bacteroidales | d__Bacteria;p__Bacteroidota;c__Bacteroidia;o__Bacteroidales;f__Bacteroidaceae;g__CAG-617;s__ |  |  |  |  |
| bin.294 | 839 | 2709.85 | 2273562 | 3231 | 61.2 |  | 85.93 | 2.742 | Clostridiales | d__Bacteria;p__Firmicutes_A;c__Clostridia;o__Oscillospirales;f__Ruminococcaceae;g__Anaerofilum;s__ |  |  |  |  |
| bin.295 | 364 | 5801.89 | 2111888 | 7652 | 50.5719 |  | 86.95 | 1.02 | Clostridiales | d__Bacteria;p__Firmicutes_A;c__Clostridia;o__Oscillospirales;f__Ruminococcaceae;g__Ruthenibacterium;s__ |  |  |  |  |
| bin.296 | 105 | 19110.5 | 2006603 | 31767 | 36.85 |  | 88.03 | 1.709 | Bacteria | d__Bacteria;p__Cyanobacteria;c__Vampirovibrionia;o__Gastranaerophilales;f__Gastranaerophilaceae;g__QAMI01;s__QAMI01 sp900551915 | 0.025771 | 0.049766 | 0.021061 | 0.056539 |
| bin.297 | 233 | 6948.39 | 1618974 | 9217 | 51.564 |  | 95.67 | 0.223 | Clostridiales | d__Bacteria;p__Firmicutes_A;c__Clostridia;o__Oscillospirales;f__Acutalibacteraceae;g__Eubacterium_R;s__ |  |  |  |  |
| bin.298 | 435 | 2710.84 | 1179217 | 2755 | 55.6256 |  | 51.25 | 0.955 | Clostridiales | d__Bacteria;p__Firmicutes_A;c__Clostridia;o__Oscillospirales;f__Butyricicoccaceae;g__Butyricicoccus;s__Butyricicoccus pullicaecorum |  |  |  |  |
| bin.299 | 328 | 4324.04 | 1418286 | 5087 | 53.7272 |  | 78.68 | 1.121 | Bacteroidetes | d__Bacteria;p__Bacteroidota;c__Bacteroidia;o__Bacteroidales;f__Rikenellaceae;g__Alistipes;s__ |  |  |  |  |
| bin.3 | 322 | 6762.07 | 2177385 | 10296 | 48.7765 |  | 91.52 | 2.034 | Clostridiales | d__Bacteria;p__Firmicutes_A;c__Clostridia;o__Monoglobales_A;f__UBA1381;g__;s__ |  |  |  |  |
| bin.30 | 459 | 4100.67 | 1882206 | 4893 | 48.166 |  | 82.04 | 1.265 | Clostridiales | d__Bacteria;p__Firmicutes_A;c__Clostridia;o__Lachnospirales;f__Lachnospiraceae;g__Blautia_A;s__ |  |  |  |  |
| bin.300 | 175 | 12791.9 | 2238580 | 19003 | 48.1754 |  | 79.51 | 0.373 | Bacteroidales | d__Bacteria;p__Bacteroidota;c__Bacteroidia;o__Bacteroidales;f__Bacteroidaceae;g__Phocaeicola;s__Phocaeicola sp900541515 |  |  |  |  |
| bin.301 | 506 | 4875.04 | 2466768 | 6403 | 49.3395 |  | 87.42 | 1.898 | Clostridiales | d__Bacteria;p__Firmicutes_A;c__Clostridia;o__Lachnospirales;f__Lachnospiraceae;g__UBA7160;s__ | 0.037051 | 0.071548 |  |  |
| bin.302 | 210 | 8602.83 | 1806595 | 17221 | 49.9662 |  | 100 | 0.067 | Bacteria | d__Bacteria;p__Spirochaetota;c__Spirochaetia;o__JC444;f__UBA9216;g__;s__ | 0.286414 | 0.553091 | 0.257861 | 0.692225 |
| bin.303 | 230 | 2748.2 | 632086 | 2848 | 27.4764 |  | 71.51 | 4.666 | Bacteria | d__Bacteria;p__Firmicutes;c__Bacilli;o__RF39;f__UBA660;g__RUG11198;s__ |  |  |  |  |
| bin.304 | 659 | 3104.14 | 2045627 | 3856 | 45.3653 |  | 79.5 | 1.602 | Bacteroidales | d__Bacteria;p__Bacteroidota;c__Bacteroidia;o__Bacteroidales;f__Tannerellaceae;g__Parabacteroides;s__ |  |  |  |  |
| bin.305 | 360 | 4037.32 | 1453437 | 5584 | 51.2883 |  | 88.3 | 0.713 | Clostridiales | d__Bacteria;p__Firmicutes_A;c__Clostridia_A;o__Christensenellales;f__UBA3700;g__;s__ | 0.16512 | 0.318862 | 0.136163 | 0.365529 |
| bin.306 | 282 | 5953.4 | 1678860 | 7662 | 50.1624 |  | 92.04 | 2.301 | Bacteroidetes | d__Bacteria;p__Bacteroidota;c__Bacteroidia;o__Bacteroidales;f__UBA932;g__UBA3382;s__UBA3382 sp002159555 | 0.033848 | 0.065363 | 0.030964 | 0.083122 |
| bin.307 | 367 | 2637.29 | 967885 | 3090 | 34.0162 |  | 72.33 | 1.172 | Bacteria | d__Bacteria;p__Firmicutes;c__Bacilli;o__RF39;f__UBA660;g__UMGS1648;s__UMGS1648 sp900553765 | 0.182935 | 0.353263 | 0.084213 | 0.226067 |
| bin.308 | 524 | 2732.31 | 1431728 | 3585 | 58.0969 |  | 55.52 | 4.746 | Clostridiales | d__Bacteria;p__Firmicutes_A;c__Clostridia;o__Lachnospirales;f__Lachnospiraceae;g__NSJ-38;s__ |  |  |  |  |
| bin.309 | 372 | 3842.76 | 1429508 | 4403 | 43.9139 |  | 65.1 | 1.704 | Bacteria | d__Bacteria;p__Spirochaetota;c__Spirochaetia;o__Sphaerochaetales;f__Sphaerochaetaceae;g__;s__ |  |  |  |  |
| bin.31 | 535 | 5051.87 | 2702750 | 6781 | 53.0958 |  | 88.96 | 0.806 | Bacteria | d__Bacteria;p__Bacteroidota;c__Bacteroidia;o__Bacteroidales;f__Marinifilaceae;g__Butyricimonas;s__ | 0.041976 | 0.081059 | 0.021506 | 0.057733 |
| bin.310 | 375 | 2764.9 | 1036838 | 2912 | 65.6227 |  | 52.82 | 1.724 | Bacteria | d__Bacteria;p__Actinobacteriota;c__Actinomycetia;o__Actinomycetales;f__Bifidobacteriaceae;g__Bifidobacterium;s__Bifidobacterium gallinarum |  |  |  |  |
| bin.311 | 223 | 6718.59 | 1498245 | 8975 | 64.6475 |  | 91.84 | 0.805 | Clostridiales | d__Bacteria;p__Firmicutes_A;c__Clostridia;o__Oscillospirales;f__Oscillospiraceae;g__UBA9475;s__ | 0.286666 | 0.553577 | 0.118955 | 0.319332 |
| bin.312 | 720 | 2076.12 | 1494808 | 2228 | 59.026 |  | 67.63 | 1.923 | Bacteroidetes | d__Bacteria;p__Bacteroidota;c__Bacteroidia;o__Bacteroidales;f__Rikenellaceae;g__Alistipes;s__ |  |  |  |  |
| bin.313 | 197 | 8242.98 | 1623868 | 18243 | 31.3354 |  | 95.78 | 1.445 | Campylobacter | d__Bacteria;p__Campylobacterota;c__Campylobacteria;o__Campylobacterales;f__Campylobacteraceae;g__Campylobacter_D;s__Campylobacter_D coli | 0.015545 | 0.030019 | 0.04581 | 0.122975 |
| bin.314 | 467 | 2186.08 | 1020899 | 2457 | 34.2762 |  | 65.45 | 3.666 | Epsilonproteobacteria | d__Bacteria;p__Campylobacterota;c__Campylobacteria;o__Campylobacterales;f__Campylobacteraceae;g__Campylobacter_D;s__Campylobacter_D avium | 0.043511 | 0.084024 | 0.022566 | 0.060577 |
| bin.315 | 521 | 2172.35 | 1131796 | 2451 | 51.8506 |  | 69.27 | 3.009 | Clostridia | d__Bacteria;p__Firmicutes_A;c__Clostridia_A;o__Christensenellales;f__DTU072;g__;s__ |  |  |  |  |
| bin.316 | 532 | 3797.86 | 2020464 | 4979 | 27.9854 |  | 97.16 | 1.435 | Bacteria | d__Bacteria;p__Firmicutes;c__Bacilli;o__Erysipelotrichales;f__Erysipelatoclostridiaceae;g__Erysipelatoclostridium;s__Erysipelatoclostridium spiroforme | 0.026619 | 0.051403 | 0.020936 | 0.056203 |
| bin.317 | 121 | 16336.2 | 1976686 | 27385 | 50.8826 |  | 96.78 | 0.809 | Bacteroidetes | d__Bacteria;p__Bacteroidota;c__Bacteroidia;o__Bacteroidales;f__UBA932;g__RC9;s__ | 0.038441 | 0.074233 | 0.020097 | 0.053949 |
| bin.318 | 210 | 7597.7 | 1595516 | 10839 | 51.5014 |  | 93.95 | 0.692 | Clostridiales | d__Bacteria;p__Firmicutes_A;c__Clostridia;o__Oscillospirales;f__Ruminococcaceae;g__UBA1409;s__UBA1409 sp002305045 | 0.069906 | 0.134995 | 0.049096 | 0.131799 |
| bin.319 | 258 | 5480.85 | 1414059 | 7666 | 47.7725 |  | 58.01 | 0.436 | Bacteroidales | d__Bacteria;p__Bacteroidota;c__Bacteroidia;o__Bacteroidales;f__Bacteroidaceae;g__Phocaeicola;s__ |  |  |  |  |
| bin.32 | 299 | 3479.4 | 1040341 | 3990 | 42.5273 |  | 79.55 | 1.428 | Bacteria | d__Bacteria;p__Firmicutes;c__Bacilli;o__Erysipelotrichales;f__Erysipelotrichaceae;g__;s__ | 0.051995 | 0.100407 |  |  |
| bin.320 | 891 | 1992.54 | 1775354 | 2073 | 58.3804 |  | 66.84 | 1.602 | Clostridiales | d__Bacteria;p__Firmicutes_A;c__Clostridia;o__Lachnospirales;f__Lachnospiraceae;g__Lachnoclostridium_A;s__ |  |  |  |  |
| bin.321 | 384 | 2919.39 | 1121044 | 3086 | 40.4199 |  | 55.69 | 6.14 | Bacteria | d__Bacteria;p__Proteobacteria;c__Alphaproteobacteria;o__RF32;f__CAG-239;g__CAG-495;s__ |  |  |  |  |
| bin.322 | 634 | 2270 | 1439180 | 2496 | 53.2765 |  | 61.71 | 0.817 | Clostridiales | d__Bacteria;p__Firmicutes_A;c__Clostridia;o__Lachnospirales;f__Lachnospiraceae;g__RUG12045;s__ | 0.05603 | 0.1082 |  |  |
| bin.323 | 650 | 3129.55 | 2034206 | 3918 | 51.6656 |  | 82.3 | 2.476 | Bacteroidetes | d__Bacteria;p__Bacteroidota;c__Bacteroidia;o__Bacteroidales;f__UBA932;g__RC9;s__ |  |  |  |  |
| bin.324 | 396 | 3500.75 | 1386298 | 3972 | 53.1758 |  | 60.92 | 0.576 | Bacteroidales | d__Bacteria;p__Bacteroidota;c__Bacteroidia;o__Bacteroidales;f__Tannerellaceae;g__Parabacteroides;s__ |  |  |  |  |
| bin.325 | 357 | 2534.85 | 904940 | 2860 | 49.0373 |  | 50.46 | 0.354 | Clostridiales | d__Bacteria;p__Firmicutes_A;c__Clostridia;o__Peptostreptococcales;f__Anaerovoracaceae;g__UBA1191;s__ |  |  |  |  |
| bin.326 | 503 | 3026.17 | 1522165 | 3125 | 61.2637 |  | 55.11 | 0 | Bacteria | d__Bacteria;p__Firmicutes_A;c__Clostridia;o__Oscillospirales;f__Ruminococcaceae;g__Gemmiger;s__ |  |  |  |  |
| bin.328 | 403 | 5916.49 | 2384347 | 7783 | 51.0818 |  | 84.15 | 1.053 | Bacteroidales | d__Bacteria;p__Bacteroidota;c__Bacteroidia;o__Bacteroidales;f__Bacteroidaceae;g__Bacteroides;s__Bacteroides sp002160055 |  |  |  |  |
| bin.329 | 293 | 6440.9 | 1887184 | 9129 | 51.6063 |  | 86.56 | 0.158 | Bacteroidetes | d__Bacteria;p__Bacteroidota;c__Bacteroidia;o__Bacteroidales;f__UBA932;g__RC9;s__ |  |  |  |  |
| bin.33 | 263 | 2842.48 | 747573 | 3625 | 45.1716 |  | 71.49 | 3.946 | Bacteria | d__Bacteria;p__Proteobacteria;c__Alphaproteobacteria;o__Rs-D84;f__Rs-D84;g__Rs-D84;s__ | 0.024219 | 0.046769 | 0.018451 | 0.049531 |
| bin.331 | 328 | 5299.62 | 1738274 | 6476 | 49.1386 |  | 89.84 | 1.285 | Bacteroidetes | d__Bacteria;p__Bacteroidota;c__Bacteroidia;o__Bacteroidales;f__UBA932;g__UBA3382;s__ | 0.036057 | 0.06963 | 0.037714 | 0.101242 |
| bin.332 | 463 | 3332.97 | 1543164 | 3862 | 31.3824 |  | 68.05 | 1.804 | Bacteria | d__Bacteria;p__Cyanobacteria;c__Vampirovibrionia;o__Gastranaerophilales;f__Gastranaerophilaceae;g__;s__ | 0.02069 | 0.039953 | 0.047647 | 0.127908 |
| bin.333 | 303 | 2775.5 | 840978 | 3060 | 32.1268 |  | 52.72 | 0 | Bacteria | d__Bacteria;p__Firmicutes;c__Bacilli;o__RF39;f__UBA660;g__UMGS2016;s__ |  |  |  |  |
| bin.334 | 283 | 6904.67 | 1954023 | 11192 | 49.9225 |  | 92.4 | 0 | Bacteroidetes | d__Bacteria;p__Bacteroidota;c__Bacteroidia;o__Bacteroidales;f__UBA932;g__RC9;s__ | 0.043289 | 0.083595 |  |  |
| bin.335 | 357 | 2866.39 | 1023301 | 3106 | 54.8037 |  | 51.62 | 0.961 | Firmicutes | d__Bacteria;p__Firmicutes_A;c__Clostridia_A;o__Christensenellales;f__DTU072;g__;s__ |  |  |  |  |
| bin.336 | 582 | 3441.73 | 2003084 | 4533 | 61.9453 |  | 84.2 | 2.564 | Bacteroidetes | d__Bacteria;p__Bacteroidota;c__Bacteroidia;o__Bacteroidales;f__Rikenellaceae;g__Alistipes;s__ |  |  |  |  |
| bin.337 | 269 | 5663.84 | 1523574 | 8277 | 49.8938 |  | 90.82 | 1.677 | Clostridiales | d__Bacteria;p__Firmicutes_A;c__Clostridia;o__Oscillospirales;f__Ruminococcaceae;g__UBA1409;s__ | 0.073796 | 0.142507 | 0.044808 | 0.120287 |
| bin.338 | 290 | 7780.32 | 2256294 | 10838 | 50.2458 |  | 90.05 | 1.901 | Clostridiales | d__Bacteria;p__Firmicutes_A;c__Clostridia;o__Lachnospirales;f__Lachnospiraceae;g__Anaerobutyricum;s__ |  |  |  |  |
| bin.339 | 307 | 4153.76 | 1275203 | 5946 | 34.3708 |  | 91.88 | 2.637 | Lactobacillus | d__Bacteria;p__Firmicutes;c__Bacilli;o__Lactobacillales;f__Lactobacillaceae;g__Lactobacillus;s__Lactobacillus johnsonii | 0.225485 | 0.435432 | 0.174985 | 0.469744 |
| bin.34 | 438 | 3794.85 | 1662145 | 4352 | 54.2307 |  | 57.01 | 0 | Bacteria | d__Bacteria;p__Firmicutes_A;c__Clostridia;o__Lachnospirales;f__Lachnospiraceae;g__AM51-8;s__ |  |  |  |  |
| bin.341 | 275 | 7069.68 | 1944162 | 11961 | 59.1601 |  | 93.17 | 2.684 | Clostridiales | d__Bacteria;p__Firmicutes_A;c__Clostridia;o__Oscillospirales;f__Oscillospiraceae;g__CAG-110;s__ |  |  |  |  |
| bin.342 | 208 | 9062.61 | 1885023 | 14765 | 50.377 |  | 87.02 | 0.476 | Bacteroidetes | d__Bacteria;p__Bacteroidota;c__Bacteroidia;o__Bacteroidales;f__UBA932;g__RC9;s__ |  |  |  |  |
| bin.343 | 639 | 1886.06 | 1205190 | 1952 | 48.7699 |  | 70.35 | 1.396 | Clostridia | d__Bacteria;p__Firmicutes_A;c__Clostridia_A;o__Christensenellales;f__UBA3700;g__CABKMX01;s__ |  |  | 0.067759 | 0.181899 |
| bin.344 | 561 | 4836.1 | 2713051 | 6672 | 49.3421 |  | 86.84 | 1.003 | Bacteroidales | d__Bacteria;p__Bacteroidota;c__Bacteroidia;o__Bacteroidales;f__Bacteroidaceae;g__Phocaeicola;s__Phocaeicola sp900546095 |  |  |  |  |
| bin.345 | 559 | 3049.38 | 1704604 | 3326 | 51.6363 |  | 62.95 | 0 | Bacteria | d__Bacteria;p__Firmicutes_A;c__Clostridia;o__Lachnospirales;f__Lachnospiraceae;g__;s__ |  |  |  |  |
| bin.346 | 291 | 4664.18 | 1357277 | 5788 | 47.9872 |  | 74.07 | 1.587 | Bacteroidetes | d__Bacteria;p__Bacteroidota;c__Bacteroidia;o__Bacteroidales;f__UBA932;g__UBA1232;s__ |  |  |  |  |
| bin.347 | 303 | 2870.81 | 869855 | 2920 | 58.0062 |  | 64.2 | 0.806 | Clostridia | d__Bacteria;p__Firmicutes_A;c__Clostridia_A;o__Christensenellales;f__CAG-917;g__UMGS1688;s__ |  |  |  |  |
| bin.348 | 663 | 2038.58 | 1351580 | 2231 | 53.3587 |  | 58.85 | 1.898 | Clostridiales | d__Bacteria;p__Firmicutes_A;c__Clostridia;o__Lachnospirales;f__Lachnospiraceae;g__;s__ |  |  |  |  |
| bin.349 | 372 | 4043.48 | 1504174 | 4807 | 47.999 |  | 66.55 | 0.503 | Bacteroidales | d__Bacteria;p__Bacteroidota;c__Bacteroidia;o__Bacteroidales;f__Muribaculaceae;g__CAG-279;s__CAG-279 sp900550025 | 0.008357 | 0.016138 |  |  |
| bin.35 | 591 | 3144.95 | 1858668 | 4007 | 63.8852 |  | 86.01 | 3.407 | Bacteria | d__Bacteria;p__Verrucomicrobiota;c__Kiritimatiellae;o__RFP12;f__UBA1067;g__W1P29-020;s__ | 0.31591 | 0.61005 | 0.336244 | 0.902643 |
| bin.350 | 175 | 6927.93 | 1212388 | 9797 | 27.416 |  | 95.5 | 0.561 | Bacteria | d__Bacteria;p__Firmicutes;c__Bacilli;o__RF39;f__UBA660;g__UBA5578;s__ | 0.218741 | 0.422408 | 0.122385 | 0.328541 |
| bin.351 | 385 | 3576.01 | 1376765 | 4029 | 56.2493 |  | 62.66 | 0.641 | Bacteroidetes | d__Bacteria;p__Bacteroidota;c__Bacteroidia;o__Bacteroidales;f__Rikenellaceae;g__Alistipes;s__ | 0.072354 | 0.139722 |  |  |
| bin.352 | 87 | 20318.8 | 1767732 | 37242 | 51.5354 |  | 97.47 | 0.476 | Bacteroidetes | d__Bacteria;p__Bacteroidota;c__Bacteroidia;o__Bacteroidales;f__UBA932;g__UBA3382;s__UBA3382 sp002358965 | 0.03897 | 0.075254 | 0.019898 | 0.053417 |
| bin.353 | 594 | 2769.28 | 1644955 | 3374 | 48.6554 |  | 83.61 | 1.855 | Clostridiales | d__Bacteria;p__Firmicutes_A;c__Clostridia;o__Oscillospirales;f__Butyricicoccaceae;g__;s__ | 0.17858 | 0.344853 | 0.152623 | 0.409714 |
| bin.355 | 779 | 2767.97 | 2156245 | 3453 | 42.9501 |  | 94.33 | 3.194 | Bacteria | d__Bacteria;p__Firmicutes;c__Bacilli;o__Erysipelotrichales;f__Erysipelatoclostridiaceae;g__CHKCI006;s__CHKCI006 sp900018345 | 0.113467 | 0.219115 | 0.088252 | 0.23691 |
| bin.356 | 967 | 2421.82 | 2341901 | 2714 | 65.0076 |  | 76.86 | 2.432 | Deltaproteobacteria | d__Bacteria;p__Desulfobacterota;c__Desulfovibrionia;o__Desulfovibrionales;f__Desulfovibrionaceae;g__Desulfovibrio;s__ | 0.057316 | 0.110683 |  |  |
| bin.357 | 601 | 1582.94 | 951348 | 1534 | 54.9604 |  | 59.53 | 9.473 | Bacteria | d__Bacteria;p__Firmicutes;c__Bacilli;o__Erysipelotrichales;f__Erysipelotrichaceae;g__Merdibacter;s__Merdibacter sp900543035 |  |  |  |  |
| bin.358 | 516 | 3559.67 | 1836789 | 3966 | 39.3598 |  | 87.97 | 1.067 | Clostridiales | d__Bacteria;p__Firmicutes_A;c__Clostridia;o__Lachnospirales;f__Anaerotignaceae;g__Anaerotignum;s__ |  |  |  |  |
| bin.359 | 503 | 5028.77 | 2529469 | 7178 | 50.2905 |  | 92.4 | 1.797 | Gammaproteobacteria | d__Bacteria;p__Proteobacteria;c__Gammaproteobacteria;o__Enterobacterales;f__Succinivibrionaceae;g__Anaerobiospirillum;s__ |  |  |  |  |
| bin.360 | 429 | 6306.68 | 2705564 | 10842 | 55.2358 |  | 90.6 | 1.143 | Bacteroidales | d__Bacteria;p__Bacteroidota;c__Bacteroidia;o__Bacteroidales;f__Bacteroidaceae;g__Paraprevotella;s__ |  |  |  |  |
| bin.361 | 394 | 3656.46 | 1440644 | 4119 | 57.2401 |  | 76.92 | 0.367 | Bacteria | d__Bacteria;p__Bacteroidota;c__Bacteroidia;o__Flavobacteriales;f__UBA1820;g__UBA1820;s__UBA1820 sp002314265 |  |  |  |  |
| bin.362 | 409 | 5543.82 | 2267422 | 7086 | 48.3986 |  | 91.96 | 0.685 | Proteobacteria | d__Bacteria;p__Proteobacteria;c__Gammaproteobacteria;o__Burkholderiales;f__Burkholderiaceae;g__Parasutterella;s__Parasutterella sp000980495 | 0.126621 | 0.244516 |  |  |
| bin.363 | 921 | 2141.01 | 1971868 | 2285 | 46.1433 |  | 71.48 | 2.002 | Bacteroidales | d__Bacteria;p__Bacteroidota;c__Bacteroidia;o__Bacteroidales;f__Bacteroidaceae;g__Phocaeicola;s__Phocaeicola sp900540105 | 0.024748 | 0.04779 | 0.010744 | 0.028841 |
| bin.364 | 324 | 4473.26 | 1449335 | 6181 | 53.2048 |  | 84.29 | 0 | Clostridiales | d__Bacteria;p__Firmicutes_A;c__Clostridia;o__Peptostreptococcales;f__Anaerovoracaceae;g__UBA1191;s__ | 0.026428 | 0.051036 |  |  |
| bin.365 | 286 | 5551.7 | 1587786 | 7171 | 48.3325 |  | 90.49 | 1.085 | Clostridiales | d__Bacteria;p__Firmicutes_A;c__Clostridia;o__Peptostreptococcales;f__Anaerovoracaceae;g__CAG-145;s__CAG-145 sp900754795 | 0.148941 | 0.287618 | 0.07312 | 0.196291 |
| bin.366 | 658 | 2739.86 | 1802825 | 3211 | 48.3159 |  | 62.33 | 3.605 | Bacteria | d__Bacteria;p__Bacteroidota;c__Bacteroidia;o__Bacteroidales;f__Muribaculaceae;g__;s__ |  |  |  |  |
| bin.367 | 354 | 7715.26 | 2731203 | 12950 | 50.2308 |  | 97.61 | 1.352 | Bacteroidales | d__Bacteria;p__Bacteroidota;c__Bacteroidia;o__Bacteroidales;f__Muribaculaceae;g__CAG-279;s__ | 0.030096 | 0.058118 | 0.01339 | 0.035946 |
| bin.368 | 565 | 4013.41 | 2267574 | 5718 | 51.8184 |  | 88.53 | 1.72 | Bacteroidales | d__Bacteria;p__Bacteroidota;c__Bacteroidia;o__Bacteroidales;f__Barnesiellaceae;g__Barnesiella;s__ |  |  |  |  |
| bin.369 | 598 | 2175.72 | 1301079 | 2398 | 54.9898 |  | 72.82 | 3.49 | Clostridiales | d__Bacteria;p__Firmicutes_A;c__Clostridia;o__Lachnospirales;f__Lachnospiraceae;g__;s__ | 0.045258 | 0.087397 | 0.046616 | 0.12514 |
| bin.37 | 672 | 1995.67 | 1341092 | 2111 | 47.52 |  | 54.17 | 2.798 | Bacteroidales | d__Bacteria;p__Bacteroidota;c__Bacteroidia;o__Bacteroidales;f__Bacteroidaceae;g__Prevotella;s__Prevotella sp000433175 | 0.045005 | 0.086908 | 0.03747 | 0.100587 |
| bin.370 | 238 | 5620.59 | 1337701 | 6981 | 56.3824 |  | 84.37 | 2.445 | Clostridia | d__Bacteria;p__Firmicutes_A;c__Clostridia_A;o__Christensenellales;f__Borkfalkiaceae;g__UBA11940;s__ |  |  |  |  |
| bin.371 | 429 | 5226.97 | 2242369 | 6604 | 50.5548 |  | 76.72 | 0 | Bacteria | d__Bacteria;p__Bacteroidota;c__Bacteroidia;o__Bacteroidales;f__Bacteroidaceae;g__Phocaeicola;s__ |  |  |  |  |
| bin.372 | 88 | 24336.6 | 2141622 | 40316 | 38.9192 |  | 95.51 | 1.282 | Bacteria | d__Bacteria;p__Eremiobacterota;c__Xenobia;o__CADAWZ01;f__CADAWZ01;g__;s__ | 0.376827 | 0.727685 | 0.272938 | 0.732698 |
| bin.373 | 376 | 11739.4 | 4414013 | 25295 | 42.7334 |  | 97.68 | 0.963 | Bacteroidales | d__Bacteria;p__Bacteroidota;c__Bacteroidia;o__Bacteroidales;f__Bacteroidaceae;g__Phocaeicola;s__Phocaeicola dorei | 0.037925 | 0.073237 | 0.020481 | 0.054981 |
| bin.374 | 939 | 2345.94 | 2202833 | 2556 | 60.7912 |  | 69.06 | 2.983 | Clostridia | d__Bacteria;p__Firmicutes_A;c__Clostridia_A;o__Christensenellales;f__CAG-74;g__UMGS1600;s__ |  |  |  |  |
| bin.375 | 387 | 4068.17 | 1574381 | 6366 | 48.171 |  | 94.38 | 1.853 | Bacteria | d__Bacteria;p__Elusimicrobiota;c__Elusimicrobia;o__Elusimicrobiales;f__Elusimicrobiaceae;g__UBA1436;s__ | 0.63699 | 1.230082 | 0.532206 | 1.428698 |
| bin.376 | 270 | 4754.72 | 1283774 | 5746 | 40.3217 |  | 80.46 | 0 | Bacteria | d__Bacteria;p__Proteobacteria;c__Alphaproteobacteria;o__RF32;f__CAG-239;g__CAG-495;s__ |  |  |  |  |
| bin.377 | 487 | 4586.58 | 2233663 | 6079 | 33.1429 |  | 88.43 | 2.037 | Bacilli | d__Bacteria;p__Firmicutes;c__Bacilli;o__Staphylococcales;f__Staphylococcaceae;g__Staphylococcus;s__Staphylococcus equorum | 0.136211 | 0.263034 | 0.116094 | 0.311652 |
| bin.378 | 198 | 7343.69 | 1454051 | 11373 | 48.019 |  | 90.42 | 0 | Clostridiales | d__Bacteria;p__Firmicutes_A;c__Clostridia;o__Peptostreptococcales;f__Anaerovoracaceae;g__;s__ | 0.105805 | 0.204318 | 0.084082 | 0.225717 |
| bin.379 | 360 | 5420.52 | 1951386 | 6977 | 60.0192 |  | 91.98 | 1.36 | Clostridiales | d__Bacteria;p__Firmicutes_A;c__Clostridia;o__Oscillospirales;f__Ruminococcaceae;g__Ruthenibacterium;s__Ruthenibacterium sp900546885 |  |  |  |  |
| bin.38 | 333 | 2708.09 | 901793 | 2783 | 51.4692 |  | 50.21 | 0.671 | Clostridiales | d__Bacteria;p__Firmicutes_A;c__Clostridia_A;o__Christensenellales;f__UBA3700;g__CABKMX01;s__ |  |  |  |  |
| bin.380 | 477 | 2160.52 | 1030568 | 2294 | 64.1449 |  | 57.31 | 1.07 | Clostridiales | d__Bacteria;p__Firmicutes_A;c__Clostridia_A;o__Christensenellales;f__Borkfalkiaceae;g__Borkfalkia;s__ |  |  |  |  |
| bin.381 | 259 | 3333.42 | 863356 | 3986 | 52.3756 |  | 62.25 | 0.455 | Clostridia | d__Bacteria;p__Firmicutes_A;c__Clostridia_A;o__Christensenellales;f__;g__;s__ | 0.175651 | 0.339198 | 0.112188 | 0.301167 |
| bin.382 | 67 | 37652.4 | 2522711 | 50210 | 46.7202 |  | 97.02 | 0 | Bacteria | d__Bacteria;p__Bacteroidota;c__Bacteroidia;o__Bacteroidales;f__Paludibacteraceae;g__;s__ | 0.076414 | 0.147562 | 0.052154 | 0.140008 |
| bin.383 | 647 | 2829.58 | 1830740 | 2944 | 40.0371 |  | 63.82 | 0.943 | Bacteroidales | d__Bacteria;p__Bacteroidota;c__Bacteroidia;o__Bacteroidales;f__Bacteroidaceae;g__;s__ |  |  |  |  |
| bin.384 | 441 | 2618.23 | 1154638 | 2647 | 56.9884 |  | 50.73 | 1.724 | Bacteria | d__Bacteria;p__Firmicutes_A;c__Clostridia;o__Oscillospirales;f__Acutalibacteraceae;g__;s__ |  |  |  |  |
| bin.385 | 303 | 7121.15 | 2157707 | 10458 | 41.8272 |  | 91.08 | 1.342 | Clostridiales | d__Bacteria;p__Firmicutes_A;c__Clostridia;o__Lachnospirales;f__Lachnospiraceae;g__Anaerostipes;s__ |  |  |  |  |
| bin.386 | 174 | 13363.1 | 2325185 | 24916 | 56.3633 |  | 86.57 | 0 | Bacteria | d__Bacteria;p__Eremiobacterota;c__Xenobia;o__Xenobiales;f__Xenobiaceae;g__;s__ | 0.404268 | 0.780677 | 0.261351 | 0.701593 |
| bin.387 | 525 | 3780.97 | 1985008 | 5093 | 40.6656 |  | 88.1 | 2.447 | Firmicutes | d__Bacteria;p__Firmicutes_C;c__Negativicutes;o__Selenomonadales;f__Selenomonadaceae;g__Megamonas;s__Megamonas sp900554895 |  |  |  |  |
| bin.388 | 446 | 4148.89 | 1850406 | 5111 | 53.9258 |  | 87.72 | 2.125 | Clostridiales | d__Bacteria;p__Firmicutes_A;c__Clostridia;o__Lachnospirales;f__Lachnospiraceae;g__;s__ |  |  |  |  |
| bin.389 | 499 | 3245.55 | 1619531 | 3452 | 51.7492 |  | 73.88 | 1.476 | Clostridiales | d__Bacteria;p__Firmicutes_A;c__Clostridia;o__Oscillospirales;f__Ruminococcaceae;g__Negativibacillus;s__ |  |  |  |  |
| bin.39 | 404 | 5542.73 | 2239261 | 6635 | 48.6288 |  | 70.81 | 0.929 | Bacteroidales | d__Bacteria;p__Bacteroidota;c__Bacteroidia;o__Bacteroidales;f__Bacteroidaceae;g__Bacteroides;s__ |  |  |  |  |
| bin.390 | 832 | 1867.75 | 1553968 | 1936 | 47.5403 |  | 56.06 | 2.865 | Clostridia | d__Bacteria;p__Firmicutes_B;c__Dehalobacteriia;o__UBA4068;f__UBA4068;g__;s__ | 0.128652 | 0.248438 | 0.064648 | 0.173546 |
| bin.391 | 379 | 3155.48 | 1195928 | 3508 | 51.8431 |  | 59.31 | 2.586 | Bacteria | d__Bacteria;p__Firmicutes_A;c__Clostridia;o__Lachnospirales;f__Lachnospiraceae;g__;s__ |  |  |  |  |
| bin.392 | 396 | 3120.81 | 1235842 | 3285 | 50.3701 |  | 61.38 | 0.968 | Bacteroidetes | d__Bacteria;p__Bacteroidota;c__Bacteroidia;o__Bacteroidales;f__UBA932;g__RC9;s__ |  |  |  |  |
| bin.393 | 770 | 3118.84 | 2401503 | 4043 | 50.5682 |  | 63.94 | 1.88 | Bacteria | d__Bacteria;p__Firmicutes_A;c__Clostridia;o__Lachnospirales;f__Lachnospiraceae;g__Mediterraneibacter;s__Mediterraneibacter sp002314255 |  |  |  |  |
| bin.394 | 440 | 3448.04 | 1517138 | 3739 | 62.6529 |  | 51.2 | 2.586 | Bacteria | d__Bacteria;p__Firmicutes_A;c__Clostridia;o__Oscillospirales;f__Oscillospiraceae;g__Evtepia;s__Evtepia sp004554585 |  |  |  |  |
| bin.395 | 462 | 2350.16 | 1085775 | 2624 | 50.508 |  | 64.09 | 1.957 | Clostridiales | d__Bacteria;p__Firmicutes_A;c__Clostridia;o__Oscillospirales;f__Butyricicoccaceae;g__;s__ |  |  |  |  |
| bin.396 | 702 | 3526.51 | 2475608 | 3857 | 54.3519 |  | 65.6 | 0 | Bacteria | d__Bacteria;p__Firmicutes_A;c__Clostridia;o__Lachnospirales;f__Lachnospiraceae;g__UBA7182;s__ |  |  |  |  |
| bin.397 | 123 | 6847.41 | 842231 | 11268 | 42.9739 |  | 87.91 | 0.099 | Bacteria | d__Bacteria;p__Proteobacteria;c__Alphaproteobacteria;o__Rs-D84;f__Rs-D84;g__Rs-D84;s__ | 0.036494 | 0.070473 | 0.022912 | 0.061507 |
| bin.398 | 477 | 3780.58 | 1803338 | 4335 | 69.2466 |  | 82.8 | 3.023 | Actinomycetaceae | d__Bacteria;p__Actinobacteriota;c__Actinomycetia;o__Actinomycetales;f__Actinomycetaceae;g__Pauljensenia;s__ | 0.168873 | 0.326108 | 0.119278 | 0.3202 |
| bin.399 | 341 | 3803.92 | 1297136 | 4310 | 51.9061 |  | 74.55 | 1.902 | Clostridiales | d__Bacteria;p__Firmicutes_A;c__Clostridia_A;o__Christensenellales;f__DTU072;g__;s__ | 0.080557 | 0.155563 | 0.072029 | 0.19336 |
| bin.4 | 710 | 1904.21 | 1351987 | 2035 | 46.8703 |  | 63.92 | 1.403 | Clostridiales | d__Bacteria;p__Firmicutes_A;c__Clostridia;o__Lachnospirales;f__Anaerotignaceae;g__UMGS1670;s__ | 0.098699 | 0.190597 | 0.070687 | 0.189758 |
| bin.40 | 440 | 3377.27 | 1485999 | 3780 | 58.5455 |  | 69.36 | 1.677 | Clostridiales | d__Bacteria;p__Firmicutes_A;c__Clostridia;o__Oscillospirales;f__Oscillospiraceae;g__UBA1777;s__ |  |  |  |  |
| bin.400 | 135 | 21540.9 | 2908019 | 38705 | 43.8533 |  | 97.29 | 0 | Bacteroidales | d__Bacteria;p__Bacteroidota;c__Bacteroidia;o__Bacteroidales;f__Barnesiellaceae;g__Barnesiella;s__Barnesiella intestinihominis |  |  |  |  |
| bin.401 | 50 | 13470.3 | 673517 | 21168 | 44.1476 |  | 65.83 | 0 | Bacteria | d__Bacteria;p__Patescibacteria;c__Saccharimonadia;o__Saccharimonadales;f__Saccharimonadaceae;g__UBA2834;s__UBA2834 sp004561445 |  |  |  |  |
| bin.402 | 141 | 6126.84 | 863885 | 8514 | 42.0737 |  | 77.34 | 7.372 | Bacteria | d__Bacteria;p__Proteobacteria;c__Alphaproteobacteria;o__Rs-D84;f__Rs-D84;g__Rs-D84;s__ | 0.019928 | 0.038482 | 0.015202 | 0.04081 |
| bin.404 | 624 | 2391.44 | 1492256 | 2749 | 49.9733 |  | 77.3 | 4.192 | Clostridiales | d__Bacteria;p__Firmicutes_A;c__Clostridia;o__Lachnospirales;f__Lachnospiraceae;g__Ruminococcus_G;s__ | 0.051543 | 0.099533 | 0.030315 | 0.081381 |
| bin.405 | 657 | 2948.47 | 1937144 | 3167 | 63.1007 |  | 64.18 | 1.824 | Bacteria | d__Bacteria;p__Verrucomicrobiota;c__Kiritimatiellae;o__RFP12;f__UBA3636;g__;s__ | 0.283246 | 0.546973 | 0.255321 | 0.685406 |
| bin.406 | 264 | 3475.06 | 917415 | 3930 | 27.1677 |  | 71.7 | 0 | Bacteria | d__Bacteria;p__Firmicutes;c__Bacilli;o__RF39;f__UBA660;g__UBA11963;s__ |  |  |  |  |
| bin.407 | 460 | 2183.23 | 1004284 | 2335 | 50.577 |  | 58.8 | 1.51 | Clostridiales | d__Bacteria;p__Firmicutes_A;c__Clostridia;o__Monoglobales_A;f__UBA1381;g__UBA1381;s__ | 0.259546 | 0.501205 | 0.184938 | 0.496463 |
| bin.408 | 496 | 2632.99 | 1305963 | 2758 | 63.6716 |  | 56.7 | 0.099 | Clostridia | d__Bacteria;p__Firmicutes_A;c__Clostridia_A;o__Christensenellales;f__CAG-74;g__JAAYOH01;s__ | 0.061498 | 0.118758 | 0.05704 | 0.153123 |
| bin.409 | 536 | 4759.53 | 2551107 | 7332 | 55.5364 |  | 91.07 | 1.307 | Gammaproteobacteria | d__Bacteria;p__Proteobacteria;c__Gammaproteobacteria;o__Enterobacterales;f__Succinivibrionaceae;g__Anaerobiospirillum_A;s__ | 0.065105 | 0.125724 | 0.044011 | 0.118147 |
| bin.41 | 349 | 6828.85 | 2383268 | 8590 | 53.0125 |  | 90.08 | 3.991 | Bacteria | d__Bacteria;p__Bacteroidota;c__Bacteroidia;o__Bacteroidales;f__Paludibacteraceae;g__;s__ |  |  |  |  |
| bin.410 | 345 | 2832.15 | 977092 | 2881 | 38.934 |  | 50.97 | 0 | Bacteria | d__Bacteria;p__Cyanobacteria;c__Vampirovibrionia;o__Gastranaerophilales;f__Gastranaerophilaceae;g__;s__ |  |  |  |  |
| bin.411 | 445 | 3100.37 | 1379663 | 3366 | 40.919 |  | 74.86 | 0 | Gammaproteobacteria | d__Bacteria;p__Proteobacteria;c__Gammaproteobacteria;o__Enterobacterales;f__Succinivibrionaceae;g__Succinatimonas;s__Succinatimonas hippei |  |  |  |  |
| bin.412 | 453 | 3060.66 | 1386481 | 3801 | 52.9342 |  | 70.95 | 0.978 | Bacteroidetes | d__Bacteria;p__Bacteroidota;c__Bacteroidia;o__Bacteroidales;f__UBA932;g__RC9;s__ |  |  |  |  |
| bin.413 | 601 | 2154.18 | 1294660 | 2343 | 50.1944 |  | 57.67 | 1.509 | Bacteroidales | d__Bacteria;p__Bacteroidota;c__Bacteroidia;o__Bacteroidales;f__Bacteroidaceae;g__UBA6398;s__UBA6398 sp900550635 |  |  |  |  |
| bin.414 | 300 | 9122.57 | 2736771 | 19828 | 55.8714 |  | 96.53 | 3.076 | Clostridiales | d__Bacteria;p__Firmicutes_A;c__Clostridia;o__Oscillospirales;f__Oscillospiraceae;g__CAG-110;s__ | 0.151699 | 0.292945 |  |  |
| bin.415 | 230 | 5213.83 | 1199181 | 6750 | 49.7837 |  | 82.57 | 2.016 | Clostridia | d__Bacteria;p__Firmicutes_A;c__Clostridia_A;o__Christensenellales;f__CAG-314;g__CAG-314;s__CAG-314 sp000437915 | 0.192559 | 0.371847 |  |  |
| bin.416 | 244 | 5763.11 | 1406199 | 7243 | 57.8722 |  | 75.95 | 1.027 | Clostridiales | d__Bacteria;p__Firmicutes_A;c__Clostridia;o__Oscillospirales;f__CAG-272;g__UBA1740;s__ | 0.237832 | 0.459274 | 0.163826 | 0.439788 |
| bin.417 | 210 | 9211.69 | 1934454 | 13858 | 44.1832 |  | 96.22 | 0.094 | Bacteria | d__Bacteria;p__Firmicutes;c__Bacilli;o__Erysipelotrichales;f__Erysipelotrichaceae;g__Merdibacter;s__Merdibacter sp900759455 | 0.021889 | 0.04227 | 0.012049 | 0.032344 |
| bin.418 | 906 | 1544.91 | 1399691 | 1507 | 62.7065 |  | 50.8 | 9.556 | Clostridiales | d__Bacteria;p__Firmicutes_A;c__Clostridia;o__Oscillospirales;f__Ruminococcaceae;g__Gemmiger;s__ |  |  |  |  |
| bin.419 | 83 | 13504.4 | 1120867 | 18586 | 46.0197 |  | 93.4 | 1.098 | Bacteria | d__Bacteria;p__Proteobacteria;c__Alphaproteobacteria;o__RF32;f__UBA3637;g__UBA7488;s__ | 0.32295 | 0.623644 | 0.216257 | 0.58054 |
| bin.42 | 239 | 2615.58 | 625123 | 3023 | 44.0309 |  | 63.12 | 1.098 | Bacteria | d__Bacteria;p__Proteobacteria;c__Alphaproteobacteria;o__Rs-D84;f__Rs-D84;g__Rs-D84;s__ | 0.028809 | 0.055633 | 0.013615 | 0.03655 |
| bin.420 | 444 | 2564.63 | 1138695 | 2608 | 41.5506 |  | 59.68 | 1.403 | Clostridiales | d__Bacteria;p__Firmicutes_A;c__Clostridia;o__Lachnospirales;f__Anaerotignaceae;g__UMGS1670;s__UMGS1670 sp902406135 |  |  |  |  |
| bin.421 | 478 | 4034.6 | 1928539 | 5217 | 49.9402 |  | 93.92 | 2.245 | Clostridiales | d__Bacteria;p__Firmicutes_A;c__Clostridia;o__Peptostreptococcales;f__Anaerovoracaceae;g__UBA1191;s__UBA1191 sp900543485 | 0.023116 | 0.04464 | 0.02662 | 0.071461 |
| bin.422 | 301 | 7927.05 | 2386043 | 11493 | 54.7068 |  | 87.13 | 2.072 | Bacteroidales | d__Bacteria;p__Bacteroidota;c__Bacteroidia;o__Bacteroidales;f__Bacteroidaceae;g__Bacteroides;s__ |  |  |  |  |
| bin.423 | 1242 | 2256.74 | 2802873 | 2511 | 48.9295 |  | 72.25 | 1.176 | Gammaproteobacteria | d__Bacteria;p__Proteobacteria;c__Gammaproteobacteria;o__Enterobacterales;f__Succinivibrionaceae;g__Anaerobiospirillum;s__ | 0.084588 | 0.163347 | 0.054028 | 0.145036 |
| bin.424 | 296 | 3282.02 | 971479 | 3668 | 39.2597 |  | 75.3 | 1.111 | Bacteria | d__Bacteria;p__Firmicutes;c__Bacilli;o__Erysipelotrichales;f__Erysipelotrichaceae;g__;s__ | 0.10637 | 0.20541 | 0.06128 | 0.164504 |
| bin.425 | 448 | 1738.65 | 778914 | 1729 | 58.6958 |  | 56.68 | 0.103 | Bacteria | d__Bacteria;p__Spirochaetota;c__Spirochaetia;o__Sphaerochaetales;f__Sphaerochaetaceae;g__Spiro-01;s__ | 0.021385 | 0.041297 | 0.019657 | 0.052768 |
| bin.426 | 412 | 3470.54 | 1429861 | 5203 | 52.0215 |  | 58.58 | 0.783 | Bacteria | d__Bacteria;p__Bacteroidota;c__Bacteroidia;o__Bacteroidales;f__UBA932;g__CAG-831;s__ |  |  |  |  |
| bin.427 | 333 | 8890.23 | 2960446 | 14873 | 43.9631 |  | 96.14 | 0.578 | Bacteroidales | d__Bacteria;p__Bacteroidota;c__Bacteroidia;o__Bacteroidales;f__Bacteroidaceae;g__Paraprevotella;s__Paraprevotella sp900546665 | 0.040775 | 0.078739 | 0.025869 | 0.069444 |
| bin.428 | 644 | 10411.1 | 6704727 | 15605 | 60.5146 |  | 99.49 | 1.629 | Pseudomonas | d__Bacteria;p__Proteobacteria;c__Gammaproteobacteria;o__Pseudomonadales;f__Pseudomonadaceae;g__Pseudomonas_E;s__Pseudomonas_E lactis | 0.240798 | 0.465002 | 0.170499 | 0.457703 |
| bin.429 | 528 | 3376.82 | 1782960 | 4631 | 49.7382 |  | 87.93 | 0.396 | Bacteroidetes | d__Bacteria;p__Bacteroidota;c__Bacteroidia;o__Bacteroidales;f__UBA932;g__RC9;s__ |  |  |  |  |
| bin.43 | 404 | 4587.51 | 1853353 | 5868 | 63.5013 |  | 72.38 | 5.128 | Clostridiales | d__Bacteria;p__Firmicutes_A;c__Clostridia_A;o__Christensenellales;f__CAG-74;g__HGM11575;s__ |  |  |  |  |
| bin.430 | 197 | 4880.7 | 961497 | 6153 | 39.4036 |  | 86.81 | 1.648 | Bacteria | d__Bacteria;p__Proteobacteria;c__Alphaproteobacteria;o__UBA3830;f__UBA3830;g__;s__ | 0.541329 | 1.045352 | 0.433711 | 1.164292 |
| bin.431 | 246 | 4759.5 | 1170838 | 5569 | 48.0999 |  | 67.99 | 3.164 | Clostridiales | d__Bacteria;p__Firmicutes_A;c__Clostridia;o__Lachnospirales;f__Lachnospiraceae;g__RUG14107;s__ |  |  |  |  |
| bin.432 | 472 | 2421.74 | 1143061 | 2580 | 27.3496 |  | 55.3 | 2.348 | Clostridiales | d__Bacteria;p__Firmicutes_A;c__Clostridia;o__TANB77;f__CAG-508;g__UMGS1994;s__ |  |  | 0.096972 | 0.26032 |
| bin.433 | 185 | 2345.27 | 433875 | 2336 | 29.0691 |  | 53.64 | 0 | Bacteria | d__Bacteria;p__Firmicutes;c__Bacilli;o__RF39;f__UBA660;g__HGM10766;s__ |  |  |  |  |
| bin.434 | 209 | 6389.32 | 1335368 | 8086 | 41.0495 |  | 94.07 | 1.51 | Lactobacillales | d__Bacteria;p__Firmicutes;c__Bacilli;o__Lactobacillales;f__Streptococcaceae;g__Streptococcus;s__Streptococcus alactolyticus | 0.190801 | 0.368453 | 0.136581 | 0.36665 |
| bin.435 | 303 | 6038.93 | 1829796 | 7751 | 56.9693 |  | 85.58 | 1.704 | Bacteria | d__Bacteria;p__Spirochaetota;c__Spirochaetia;o__Sphaerochaetales;f__Sphaerochaetaceae;g__;s__ |  |  |  |  |
| bin.436 | 192 | 2843.66 | 545982 | 3084 | 27.7655 |  | 58.26 | 0 | Bacteria | d__Bacteria;p__Firmicutes;c__Bacilli;o__RFN20;f__CAG-826;g__UBA4855;s__UBA4855 sp900540365 | 0.132588 | 0.256039 | 0.110893 | 0.297691 |
| bin.437 | 179 | 17113.5 | 3063324 | 34757 | 58.3475 |  | 98.33 | 2.764 | Bacteroidetes | d__Bacteria;p__Bacteroidota;c__Bacteroidia;o__Bacteroidales;f__Rikenellaceae;g__Alistipes;s__Alistipes finegoldii |  |  |  |  |
| bin.438 | 367 | 6576.79 | 2413681 | 12638 | 40.0991 |  | 94.25 | 1.569 | Bacteroidales | d__Bacteria;p__Bacteroidota;c__Bacteroidia;o__Bacteroidales;f__Bacteroidaceae;g__Phocaeicola;s__ | 0.045838 | 0.088517 | 0.022245 | 0.059717 |
| bin.439 | 555 | 3512.28 | 1949314 | 5046 | 56.0075 |  | 87.84 | 1.846 | Bacteroidales | d__Bacteria;p__Bacteroidota;c__Bacteroidia;o__Bacteroidales;f__Bacteroidaceae;g__;s__ | 0.033358 | 0.064418 | 0.02815 | 0.075569 |
| bin.44 | 200 | 4582.24 | 916448 | 5840 | 49.9714 |  | 63.08 | 0 | Bacteria | d__Bacteria;p__Firmicutes_A;c__Clostridia;o__Lachnospirales;f__Lachnospiraceae;g__Merdimonas;s__ |  |  |  |  |
| bin.440 | 350 | 3873.35 | 1355671 | 4813 | 48.8945 |  | 79.08 | 0.903 | Clostridiales | d__Bacteria;p__Firmicutes_A;c__Clostridia;o__Lachnospirales;f__Lachnospiraceae;g__;s__ | 0.11092 | 0.214196 |  |  |
| bin.441 | 585 | 2604.99 | 1523919 | 3108 | 51.5861 |  | 86.55 | 4.366 | Bacteria | d__Bacteria;p__Firmicutes;c__Bacilli;o__RFN20;f__CAG-826;g__UBA733;s__ | 0.148454 | 0.286678 |  |  |
| bin.442 | 302 | 2609.21 | 787980 | 2638 | 31.837 |  | 55.2 | 1.037 | Epsilonproteobacteria | d__Bacteria;p__Campylobacterota;c__Campylobacteria;o__Campylobacterales;f__Campylobacteraceae;g__Campylobacter_D;s__ | 0.044699 | 0.086318 | 0.027753 | 0.074502 |
| bin.444 | 266 | 2772.12 | 737385 | 2846 | 29.7423 |  | 57.89 | 3.508 | Bacteria | d__Bacteria;p__Firmicutes;c__Bacilli;o__RFN20;f__CAG-826;g__UBA4855;s__ |  |  |  |  |
| bin.445 | 422 | 2502.5 | 1056056 | 2880 | 37.326 |  | 67.94 | 2.074 | Lactobacillus | d__Bacteria;p__Firmicutes;c__Bacilli;o__Lactobacillales;f__Lactobacillaceae;g__Lactobacillus;s__Lactobacillus crispatus |  |  |  |  |
| bin.446 | 284 | 5167.65 | 1467612 | 6291 | 48.1369 |  | 72.12 | 0 | Clostridiales | d__Bacteria;p__Firmicutes_A;c__Clostridia;o__Peptostreptococcales;f__Anaerovoracaceae;g__CAG-145;s__CAG-145 sp900542565 |  |  |  |  |
| bin.447 | 621 | 2976.91 | 1848659 | 3886 | 45.5404 |  | 72.32 | 2.593 | Bacteroidales | d__Bacteria;p__Bacteroidota;c__Bacteroidia;o__Bacteroidales;f__Bacteroidaceae;g__Paraprevotella;s__ | 0.028306 | 0.054661 | 0.008205 | 0.022026 |
| bin.448 | 581 | 1955.24 | 1135993 | 2040 | 47.5974 |  | 51.53 | 4.39 | Bacteria | d__Bacteria;p__Bacteroidota;c__Bacteroidia;o__Bacteroidales;f__Marinifilaceae;g__Odoribacter;s__ | 0.072127 | 0.139284 | 0.059123 | 0.158715 |
| bin.449 | 592 | 2515.42 | 1489127 | 2915 | 51.0304 |  | 79.91 | 4.051 | Lactobacillales | d__Bacteria;p__Firmicutes;c__Bacilli;o__Lactobacillales;f__Lactobacillaceae;g__Limosilactobacillus;s__ | 0.029569 | 0.0571 |  |  |
| bin.45 | 267 | 3994.32 | 1066484 | 5649 | 54.3266 |  | 57.86 | 1.19 | Bacteroidetes | d__Bacteria;p__Bacteroidota;c__Bacteroidia;o__Bacteroidales;f__UBA932;g__CAG-831;s__ |  |  |  |  |
| bin.450 | 226 | 7474.68 | 1689277 | 11652 | 47.0043 |  | 97.31 | 0.223 | Clostridiales | d__Bacteria;p__Firmicutes_A;c__Clostridia;o__Oscillospirales;f__Acutalibacteraceae;g__CAG-180;s__ | 0.123931 | 0.239321 | 0.069421 | 0.18636 |
| bin.451 | 381 | 1687.1 | 642784 | 1711 | 29.4555 |  | 51.52 | 4.775 | Bacteria | d__Bacteria;p__Firmicutes;c__Bacilli;o__RFN20;f__CAG-826;g__UBA4855;s__ |  |  |  |  |
| bin.452 | 549 | 2613.56 | 1434844 | 2701 | 65.1399 |  | 51.39 | 3.225 | Actinobacteria | d__Bacteria;p__Actinobacteriota;c__Coriobacteriia;o__Coriobacteriales;f__Eggerthellaceae;g__;s__ |  |  |  |  |
| bin.453 | 273 | 11490.9 | 3137027 | 17651 | 48.3006 |  | 97.44 | 0.69 | Clostridiales | d__Bacteria;p__Firmicutes_A;c__Clostridia;o__Lachnospirales;f__Lachnospiraceae;g__UMGS1370;s__ | 0.063327 | 0.122291 | 0.045028 | 0.120878 |
| bin.454 | 33 | 34292.3 | 1131646 | 70506 | 33.5434 |  | 95.5 | 0.561 | Bacteria | d__Bacteria;p__Firmicutes;c__Bacilli;o__RFN20;f__CAG-826;g__;s__ | 0.260889 | 0.503799 | 0.241939 | 0.649481 |
| bin.455 | 344 | 6878.78 | 2366302 | 10344 | 58.0687 |  | 81.16 | 1.141 | Bacteroidales | d__Bacteria;p__Bacteroidota;c__Bacteroidia;o__Bacteroidales;f__Bacteroidaceae;g__Prevotella;s__ | 0.055636 | 0.107437 | 0.03861 | 0.103648 |
| bin.457 | 497 | 4167.57 | 2071280 | 4930 | 70.6749 |  | 82.27 | 0.853 | Actinomycetales | d__Bacteria;p__Actinobacteriota;c__Actinomycetia;o__Actinomycetales;f__Brevibacteriaceae;g__Brevibacterium;s__ | 0.226506 | 0.437402 | 0.135728 | 0.364359 |
| bin.458 | 430 | 3614.82 | 1554373 | 4674 | 36.1151 |  | 76.87 | 3.651 | Bacteria | d__Bacteria;p__Cyanobacteria;c__Vampirovibrionia;o__Gastranaerophilales;f__Gastranaerophilaceae;g__QAMI01;s__ | 0.029618 | 0.057195 | 0.012687 | 0.034057 |
| bin.459 | 444 | 2193.37 | 973857 | 2376 | 55.4607 |  | 67.71 | 0.022 | Clostridiales | d__Bacteria;p__Firmicutes_A;c__Clostridia;o__Peptostreptococcales;f__Anaerovoracaceae;g__Mogibacterium;s__ |  |  | 0.141729 | 0.38047 |
| bin.46 | 541 | 2797.64 | 1513523 | 3583 | 56.7533 |  | 52.39 | 1.754 | Bacteria | d__Bacteria;p__Firmicutes_A;c__Clostridia;o__Lachnospirales;f__Lachnospiraceae;g__Lachnoclostridium_B;s__Lachnoclostridium_B sp002160985 |  |  |  |  |
| bin.460 | 341 | 3231.6 | 1101975 | 3406 | 62.9357 |  | 57.59 | 2.516 | Clostridiales | d__Bacteria;p__Firmicutes_A;c__Clostridia;o__Oscillospirales;f__Ruminococcaceae;g__UMGS966;s__ |  |  |  |  |
| bin.461 | 642 | 3070.89 | 1971513 | 3945 | 63.7863 |  | 80 | 1.949 | Bacteroidetes | d__Bacteria;p__Bacteroidota;c__Bacteroidia;o__Bacteroidales;f__Rikenellaceae;g__Alistipes;s__Alistipes sp900290115 |  |  |  |  |
| bin.462 | 232 | 7332.92 | 1701238 | 9371 | 55.7417 |  | 93.69 | 0.961 | Bacteroidetes | d__Bacteria;p__Bacteroidota;c__Bacteroidia;o__Bacteroidales;f__Rikenellaceae;g__Alistipes;s__Alistipes sp900550925 |  |  |  |  |
| bin.463 | 267 | 2664.9 | 711527 | 2725 | 53.3069 |  | 60.34 | 0.264 | Clostridia | d__Bacteria;p__Firmicutes_A;c__Clostridia_A;o__Christensenellales;f__CAG-314;g__CAG-1435;s__ | 0.032854 | 0.063444 |  |  |
| bin.464 | 206 | 7926.55 | 1632870 | 12118 | 61.1211 |  | 96.1 | 0.897 | Bacteroidetes | d__Bacteria;p__Bacteroidota;c__Bacteroidia;o__Bacteroidales;f__Rikenellaceae;g__Tidjanibacter;s__ | 0.027378 | 0.05287 | 0.019883 | 0.053376 |
| bin.465 | 2056 | 2735.35 | 5623885 | 3345 | 53.9434 |  | 54.73 | 9.482 | Bacteria | d__Bacteria;p__Bacteroidota;c__Bacteroidia;o__Bacteroidales;f__Bacteroidaceae;g__Bacteroides;s__ |  |  |  |  |
| bin.466 | 421 | 2649.23 | 1115324 | 2703 | 56.8161 |  | 51.72 | 0.838 | Clostridiales | d__Bacteria;p__Firmicutes_A;c__Clostridia;o__Oscillospirales;f__Butyricicoccaceae;g__AM07-15;s__ |  |  |  |  |
| bin.467 | 33 | 30873.3 | 1018818 | 54219 | 37.7054 |  | 97.46 | 0 | Bacteria | d__Bacteria;p__Chlamydiota;c__Chlamydiia;o__Chlamydiales;f__Chlamydiaceae;g__Chlamydophila;s__Chlamydophila gallinacea | 0.705397 | 1.362183 | 0.533874 | 1.433176 |
| bin.468 | 374 | 8907.5 | 3331404 | 12593 | 40.2408 |  | 97.17 | 0.716 | Bacteria | d__Bacteria;p__Bacteroidota;c__Bacteroidia;o__Bacteroidales;f__Marinifilaceae;g__Odoribacter;s__Odoribacter laneus | 0.059628 | 0.115148 | 0.03856 | 0.103512 |
| bin.469 | 411 | 4936.26 | 2028803 | 6072 | 48.2162 |  | 88.94 | 1.489 | Clostridiales | d__Bacteria;p__Firmicutes_A;c__Clostridia;o__Peptostreptococcales;f__Anaerovoracaceae;g__BX12;s__BX12 sp014333425 | 0.058179 | 0.112348 | 0.054295 | 0.145755 |
| bin.47 | 267 | 7455.05 | 1990499 | 10666 | 46.1285 |  | 85.26 | 2.272 | Bacteria | d__Bacteria;p__Spirochaetota;c__Spirochaetia;o__Sphaerochaetales;f__Sphaerochaetaceae;g__;s__ |  |  |  |  |
| bin.470 | 452 | 3809.76 | 1722013 | 4397 | 42.3267 |  | 64.02 | 0.867 | Bacteroidales | d__Bacteria;p__Bacteroidota;c__Bacteroidia;o__Bacteroidales;f__Bacteroidaceae;g__Paraprevotella;s__ |  |  |  |  |
| bin.471 | 443 | 3371.41 | 1493533 | 3850 | 55.89 |  | 88.06 | 1.859 | Bacteria | d__Bacteria;p__Spirochaetota;c__Spirochaetia;o__Sphaerochaetales;f__Sphaerochaetaceae;g__;s__ | 0.064889 | 0.125306 | 0.019992 | 0.053669 |
| bin.472 | 356 | 3821.69 | 1360520 | 5170 | 49.7001 |  | 90.11 | 1.629 | Bacteroidetes | d__Bacteria;p__Bacteroidota;c__Bacteroidia;o__Bacteroidales;f__Rikenellaceae;g__Tidjanibacter;s__ | 0.035365 | 0.068292 | 0.026924 | 0.072277 |
| bin.473 | 467 | 3122.44 | 1458179 | 3321 | 51.8042 |  | 56.92 | 0.238 | Bacteroidetes | d__Bacteria;p__Bacteroidota;c__Bacteroidia;o__Bacteroidales;f__UBA932;g__RC9;s__ |  |  |  |  |
| bin.474 | 443 | 2973.12 | 1317092 | 3077 | 62.7422 |  | 50.78 | 1.724 | Bacteria | d__Bacteria;p__Actinobacteriota;c__Coriobacteriia;o__Coriobacteriales;f__Coriobacteriaceae;g__Enorma;s__Enorma sp900538305 |  |  |  |  |
| bin.475 | 322 | 7110.25 | 2289499 | 10382 | 56.8411 |  | 94.34 | 2.839 | Clostridiales | d__Bacteria;p__Firmicutes_A;c__Clostridia;o__Oscillospirales;f__Oscillospiraceae;g__CAG-110;s__CAG-110 sp900546915 | 0.039462 | 0.076204 | 0.01974 | 0.052991 |
| bin.477 | 551 | 5809.63 | 3201104 | 7539 | 48.8766 |  | 58.22 | 2.037 | Bacteria | d__Bacteria;p__Firmicutes_A;c__Clostridia;o__Lachnospirales;f__Lachnospiraceae;g__Mediterraneibacter;s__ |  |  |  |  |
| bin.478 | 725 | 3263.46 | 2366011 | 4288 | 55.0078 |  | 63.35 | 6.034 | Bacteria | d__Bacteria;p__Bacteroidota;c__Bacteroidia;o__Bacteroidales;f__Barnesiellaceae;g__Barnesiella;s__Barnesiella sp002159975 |  |  |  |  |
| bin.479 | 387 | 3465.06 | 1340980 | 4553 | 52.4706 |  | 91.02 | 1.195 | Lactobacillales | d__Bacteria;p__Firmicutes;c__Bacilli;o__Lactobacillales;f__Lactobacillaceae;g__Limosilactobacillus;s__ |  |  |  |  |
| bin.48 | 416 | 2785.76 | 1158878 | 3543 | 49.525 |  | 59.97 | 3.958 | Proteobacteria | d__Bacteria;p__Proteobacteria;c__Gammaproteobacteria;o__Burkholderiales;f__Burkholderiaceae;g__CAG-521;s__ |  |  |  |  |
| bin.480 | 315 | 6224.14 | 1960604 | 8078 | 45.0574 |  | 96.85 | 1.677 | Clostridiales | d__Bacteria;p__Firmicutes_A;c__Clostridia;o__Oscillospirales;f__Acutalibacteraceae;g__Eubacterium_R;s__Eubacterium_R sp000431535 | 0.148867 | 0.287476 |  |  |
| bin.481 | 368 | 3231.07 | 1189035 | 3635 | 62.5833 |  | 64.58 | 1.318 | Proteobacteria | d__Bacteria;p__Proteobacteria;c__Gammaproteobacteria;o__Burkholderiales;f__Burkholderiaceae;g__Sutterella;s__Sutterella sp900543805 | 0.158203 | 0.305504 | 0.277828 | 0.745827 |
| bin.482 | 299 | 4268.49 | 1276279 | 4944 | 50.8079 |  | 78.36 | 1.23 | Clostridiales | d__Bacteria;p__Firmicutes_A;c__Clostridia_A;o__Christensenellales;f__CAG-552;g__UMGS1795;s__UMGS1795 sp900761165 | 0.330191 | 0.637628 | 0.230475 | 0.618707 |
| bin.483 | 744 | 2338.82 | 1740085 | 2643 | 50.9686 |  | 74.7 | 1.803 | Bacteroidales | d__Bacteria;p__Bacteroidota;c__Bacteroidia;o__Bacteroidales;f__Bacteroidaceae;g__Phocaeicola;s__ | 0.0495 | 0.09559 | 0.047277 | 0.126914 |
| bin.484 | 784 | 2634.78 | 2065665 | 3163 | 58.6942 |  | 55.76 | 4.032 | Euryarchaeota | d__Archaea;p__Thermoplasmatota;c__Thermoplasmata;o__Methanomassiliicoccales;f__Methanomethylophilaceae;g__UBA71;s__ |  |  |  |  |
| bin.485 | 558 | 2694.46 | 1503511 | 3234 | 62.262 |  | 74.27 | 2.089 | Bacteria | d__Bacteria;p__Verrucomicrobiota;c__Verrucomicrobiae;o__Opitutales;f__UBA953;g__W0P29-029;s__ | 0.538865 | 1.040595 | 0.474594 | 1.27404 |
| bin.486 | 405 | 2519.67 | 1020466 | 2569 | 57.0617 |  | 50.1 | 3.443 | Clostridiales | d__Bacteria;p__Firmicutes_A;c__Clostridia;o__Oscillospirales;f__Ruminococcaceae;g__Gemmiger;s__ |  |  | 0.097075 | 0.260598 |
| bin.487 | 455 | 2844.84 | 1294401 | 2914 | 45.0529 |  | 62.03 | 2.215 | Firmicutes | d__Bacteria;p__Firmicutes_C;c__Negativicutes;o__Selenomonadales;f__Selenomonadaceae;g__Megamonas;s__Megamonas hypermegale_A |  |  |  |  |
| bin.488 | 353 | 2525.8 | 891608 | 2808 | 29.4852 |  | 58.49 | 0.894 | Clostridiales | d__Bacteria;p__Firmicutes_A;c__Clostridia;o__TANB77;f__CAG-508;g__CAG-269;s__ | 0.126959 | 0.245168 | 0.084709 | 0.2274 |
| bin.489 | 521 | 4129.42 | 2151428 | 6052 | 53.6135 |  | 91.62 | 2.32 | Clostridiales | d__Bacteria;p__Firmicutes_A;c__Clostridia;o__Lachnospirales;f__Lachnospiraceae;g__OF09-33XD;s__ | 0.077184 | 0.14905 | 0.067285 | 0.180627 |
| bin.49 | 331 | 3955.9 | 1309403 | 5078 | 45.8827 |  | 87.23 | 0 | Clostridia | d__Bacteria;p__Firmicutes_A;c__Clostridia_A;o__Christensenellales;f__CAG-314;g__CAG-1435;s__CAG-1435 sp000433775 | 0.038484 | 0.074316 | 0.121674 | 0.326633 |
| bin.490 | 266 | 2888.08 | 768230 | 2916 | 29.6046 |  | 69.47 | 0 | Bacteria | d__Bacteria;p__Firmicutes;c__Bacilli;o__RFN20;f__CAG-826;g__UBA4855;s__ | 0.143259 | 0.276646 | 0.128044 | 0.343732 |
| bin.491 | 602 | 1841.72 | 1108713 | 1890 | 53.3227 |  | 62.98 | 3.166 | Bacteroidales | d__Bacteria;p__Bacteroidota;c__Bacteroidia;o__Bacteroidales;f__Bacteroidaceae;g__;s__ | 0.04787 | 0.09244 | 0.050358 | 0.135185 |
| bin.492 | 501 | 2997.69 | 1501844 | 3105 | 51.0835 |  | 64.52 | 0.244 | Clostridiales | d__Bacteria;p__Firmicutes_A;c__Clostridia;o__Oscillospirales;f__Acutalibacteraceae;g__;s__ | 0.145924 | 0.281791 | 0.082935 | 0.222638 |
| bin.493 | 322 | 5238.61 | 1686834 | 9079 | 52.6194 |  | 90.03 | 1.174 | Clostridiales | d__Bacteria;p__Firmicutes_A;c__Clostridia_A;o__Christensenellales;f__UBA3700;g__CABKMX01;s__ | 0.106722 | 0.20609 | 0.070802 | 0.190067 |
| bin.494 | 462 | 3233.37 | 1493817 | 3641 | 61.4021 |  | 51.01 | 0 | Bacteria | d__Bacteria;p__Firmicutes_A;c__Clostridia;o__Oscillospirales;f__Oscillospiraceae;g__UBA9475;s__ |  |  |  |  |
| bin.495 | 511 | 3572.5 | 1825549 | 4021 | 58.109 |  | 61.28 | 0 | Bacteria | d__Bacteria;p__Bacteroidota;c__Bacteroidia;o__Bacteroidales;f__Rikenellaceae;g__Alistipes;s__Alistipes sp900546065 |  |  |  |  |
| bin.496 | 550 | 3368.86 | 1852871 | 4246 | 56.6246 |  | 76.3 | 3.074 | Clostridiales | d__Bacteria;p__Firmicutes_A;c__Clostridia_A;o__Christensenellales;f__Borkfalkiaceae;g__Borkfalkia;s__ | 0.119164 | 0.230116 |  |  |
| bin.497 | 403 | 5508.71 | 2220012 | 7962 | 44.1892 |  | 90.99 | 3.02 | Clostridiales | d__Bacteria;p__Firmicutes_A;c__Clostridia;o__Lachnospirales;f__Lachnospiraceae;g__Anaerostipes;s__ |  |  |  |  |
| bin.498 | 295 | 2605.38 | 768587 | 2803 | 48.4465 |  | 57.16 | 2.15 | Bacteria | d__Bacteria;p__Proteobacteria;c__Alphaproteobacteria;o__RF32;f__CAG-239;g__CAG-495;s__ |  |  |  |  |
| bin.499 | 625 | 3209.75 | 2006092 | 3529 | 59.7901 |  | 65.81 | 5.948 | Bacteria | d__Bacteria;p__Verrucomicrobiota;c__Lentisphaeria;o__Victivallales;f__Victivallaceae;g__Victivallis;s__ |  |  |  |  |
| bin.5 | 514 | 3444.54 | 1770495 | 3935 | 63.7956 |  | 53.84 | 1.785 | Bacteria | d__Bacteria;p__Firmicutes_A;c__Clostridia;o__Oscillospirales;f__Ruminococcaceae;g__Anaerofilum;s__Anaerofilum sp002160015 |  |  |  |  |
| bin.50 | 270 | 8381.75 | 2263072 | 12448 | 61.5065 |  | 96.55 | 3.766 | Bacteroidetes | d__Bacteria;p__Bacteroidota;c__Bacteroidia;o__Bacteroidales;f__Rikenellaceae;g__Alistipes;s__Alistipes sp900021155 |  |  |  |  |
| bin.500 | 182 | 15756.3 | 2867643 | 23950 | 59.3812 |  | 93.9 | 0.841 | Bacteroidetes | d__Bacteria;p__Bacteroidota;c__Bacteroidia;o__Bacteroidales;f__Rikenellaceae;g__Alistipes;s__Alistipes onderdonkii |  |  |  |  |
| bin.501 | 159 | 14400.8 | 2289724 | 32997 | 50.32 |  | 98.42 | 0.377 | Bacteroidales | d__Bacteria;p__Bacteroidota;c__Bacteroidia;o__Bacteroidales;f__UBA11471;g__;s__ | 0.091612 | 0.176911 | 0.080753 | 0.216781 |
| bin.502 | 450 | 3285.65 | 1478541 | 3438 | 54.6562 |  | 79.22 | 2.444 | Selenomonadales | d__Bacteria;p__Firmicutes_C;c__Negativicutes;o__Veillonellales;f__Megasphaeraceae;g__Megasphaera;s__Megasphaera stantonii | 0.223316 | 0.431242 | 0.170978 | 0.458989 |
| bin.503 | 632 | 2763.11 | 1746288 | 3186 | 61.2295 |  | 82.05 | 1.006 | Bacteroidales | d__Bacteria;p__Bacteroidota;c__Bacteroidia;o__Bacteroidales;f__Bacteroidaceae;g__CAG-617;s__ | 0.053171 | 0.102678 | 0.03472 | 0.093204 |
| bin.504 | 593 | 2780.07 | 1648582 | 3482 | 45.9526 |  | 74.6 | 2.83 | Bacteroidales | d__Bacteria;p__Bacteroidota;c__Bacteroidia;o__Bacteroidales;f__Muribaculaceae;g__CAG-279;s__ | 0.026402 | 0.050984 | 0.011928 | 0.032022 |
| bin.505 | 598 | 1665.82 | 996160 | 1642 | 28.4199 |  | 50.87 | 8.771 | Bacteria | d__Bacteria;p__Firmicutes_A;c__Clostridia;o__TANB77;f__CAG-508;g__CAG-273;s__ |  |  |  |  |
| bin.506 | 449 | 3043.29 | 1366438 | 3212 | 37.2173 |  | 67.57 | 1.709 | Bacteria | d__Bacteria;p__Cyanobacteria;c__Vampirovibrionia;o__Gastranaerophilales;f__Gastranaerophilaceae;g__;s__ | 0.150301 | 0.290244 |  |  |
| bin.507 | 373 | 2273.75 | 848107 | 2525 | 30.8608 |  | 68.44 | 2.787 | Bacteria | d__Bacteria;p__Firmicutes;c__Bacilli;o__RF39;f__UBA660;g__UMGS2016;s__UMGS2016 sp900557185 |  |  |  |  |
| bin.508 | 265 | 2566.25 | 680057 | 2902 | 44.1248 |  | 62.54 | 3.396 | Bacteria | d__Bacteria;p__Proteobacteria;c__Alphaproteobacteria;o__Rs-D84;f__Rs-D84;g__Rs-D84;s__ | 0.028974 | 0.055951 | 0.013697 | 0.036769 |
| bin.509 | 544 | 3769.22 | 2050454 | 5127 | 53.0509 |  | 86.36 | 2.442 | Lachnospiraceae | d__Bacteria;p__Firmicutes_A;c__Clostridia;o__Lachnospirales;f__Lachnospiraceae;g__Eubacterium_I;s__ | 0.118283 | 0.228414 |  |  |
| bin.51 | 541 | 4293.95 | 2323027 | 5193 | 53.2141 |  | 68.96 | 0.862 | Bacteria | d__Bacteria;p__Bacteroidota;c__Bacteroidia;o__Bacteroidales;f__Bacteroidaceae;g__CAG-462;s__CAG-462 sp003489705 |  |  |  |  |
| bin.510 | 322 | 3676.8 | 1183929 | 4135 | 49.8038 |  | 72.91 | 1.442 | Firmicutes | d__Bacteria;p__Firmicutes_A;c__Clostridia_A;o__Christensenellales;f__Borkfalkiaceae;g__UBA11940;s__ |  |  |  |  |
| bin.511 | 858 | 3049.78 | 2616715 | 3822 | 58.9422 |  | 88.03 | 1.183 | Deltaproteobacteria | d__Bacteria;p__Desulfobacterota;c__Desulfovibrionia;o__Desulfovibrionales;f__Desulfovibrionaceae;g__Desulfovibrio;s__ | 0.018173 | 0.035094 |  |  |
| bin.512 | 737 | 2428.53 | 1789824 | 2775 | 63.9836 |  | 76.82 | 3.621 | Bacteroidetes | d__Bacteria;p__Bacteroidota;c__Bacteroidia;o__Bacteroidales;f__Rikenellaceae;g__Alistipes;s__Alistipes sp900544265 |  |  |  |  |
| bin.513 | 276 | 4212.56 | 1162667 | 5719 | 40.1133 |  | 83.11 | 1.173 | Bacteria | d__Bacteria;p__Proteobacteria;c__Alphaproteobacteria;o__RF32;f__CAG-239;g__CAAFZY01;s__ |  |  |  |  |
| bin.514 | 513 | 4162.88 | 2135555 | 5690 | 46.8503 |  | 94.77 | 0 | Deltaproteobacteria | d__Bacteria;p__Desulfobacterota;c__Desulfovibrionia;o__Desulfovibrionales;f__Desulfovibrionaceae;g__Mailhella;s__ | 0.159823 | 0.308632 | 0.10929 | 0.293388 |
| bin.515 | 258 | 2404.5 | 620360 | 2356 | 36.3027 |  | 54.9 | 0 | Bacteria | d__Bacteria;p__Proteobacteria;c__Alphaproteobacteria;o__RF32;f__CAG-239;g__CAG-495;s__ |  |  |  |  |
| bin.516 | 343 | 4182.58 | 1434625 | 4924 | 55.7791 |  | 73.23 | 0 | Proteobacteria | d__Bacteria;p__Proteobacteria;c__Gammaproteobacteria;o__Burkholderiales;f__Burkholderiaceae;g__Duodenibacillus;s__ |  |  |  |  |
| bin.517 | 235 | 6469.92 | 1520432 | 12272 | 30.31 |  | 86.85 | 0.783 | Campylobacter | d__Bacteria;p__Campylobacterota;c__Campylobacteria;o__Campylobacterales;f__Campylobacteraceae;g__Campylobacter_D;s__Campylobacter_D jejuni | 0.021943 | 0.042374 |  |  |
| bin.518 | 345 | 3275.9 | 1130184 | 3738 | 29.2001 |  | 65.26 | 2.87 | Bacteria | d__Bacteria;p__Firmicutes;c__Bacilli;o__RF39;f__UBA660;g__CAG-460;s__ |  |  |  |  |
| bin.519 | 317 | 2673.12 | 847378 | 2793 | 53.073 |  | 51 | 0 | Bacteria | d__Bacteria;p__Proteobacteria;c__Alphaproteobacteria;o__RF32;f__CAG-239;g__51-20;s__51-20 sp001917175 |  |  |  |  |
| bin.52 | 504 | 4562.65 | 2299575 | 5472 | 43.5805 |  | 77.86 | 2.168 | Bacteroidales | d__Bacteria;p__Bacteroidota;c__Bacteroidia;o__Bacteroidales;f__Bacteroidaceae;g__43-108;s__43-108 sp001915545 | 0.125677 | 0.242694 | 0.113859 | 0.305654 |
| bin.520 | 624 | 2005.14 | 1251205 | 2217 | 52.6858 |  | 54.99 | 0 | Bacteria | d__Bacteria;p__Firmicutes_A;c__Clostridia;o__Lachnospirales;f__Lachnospiraceae;g__Lachnoclostridium_B;s__Lachnoclostridium_B phocaeensis |  |  |  |  |
| bin.521 | 542 | 5419.77 | 2937517 | 7048 | 53.7419 |  | 52.58 | 4.31 | Bacteria | d__Bacteria;p__Bacteroidota;c__Bacteroidia;o__Bacteroidales;f__Bacteroidaceae;g__Bacteroides;s__ |  |  |  |  |
| bin.522 | 588 | 2889.58 | 1699072 | 3739 | 52.4182 |  | 75.76 | 3.455 | Bacteroidetes | d__Bacteria;p__Bacteroidota;c__Bacteroidia;o__Bacteroidales;f__UBA932;g__RC9;s__ |  |  |  |  |
| bin.524 | 275 | 2948.13 | 810735 | 2965 | 36.2292 |  | 55.17 | 0 | Bacteria | d__Bacteria;p__Firmicutes;c__Bacilli;o__Lactobacillales;f__Lactobacillaceae;g__Lactobacillus;s__Lactobacillus gallinarum |  |  |  |  |
| bin.525 | 758 | 4251.19 | 3222402 | 4988 | 60.4174 |  | 87.77 | 0.821 | Deltaproteobacteria | d__Bacteria;p__Desulfobacterota;c__Desulfovibrionia;o__Desulfovibrionales;f__Desulfovibrionaceae;g__Bilophila;s__Bilophila wadsworthia | 0.12007 | 0.231865 | 0.057133 | 0.153373 |
| bin.526 | 540 | 3039.43 | 1641292 | 3688 | 45.1108 |  | 76.86 | 1.392 | Clostridiales | d__Bacteria;p__Firmicutes_A;c__Clostridia;o__Lachnospirales;f__Lachnospiraceae;g__;s__ | 0.068961 | 0.13317 |  |  |
| bin.527 | 473 | 3098.31 | 1465499 | 4062 | 48.5659 |  | 86.34 | 0.502 | Bacteroidetes | d__Bacteria;p__Bacteroidota;c__Bacteroidia;o__Bacteroidales;f__UBA932;g__UBA3382;s__ | 0.035454 | 0.068464 | 0.018473 | 0.04959 |
| bin.528 | 504 | 3411.29 | 1719291 | 4696 | 54.0644 |  | 61.59 | 1.018 | Bacteroidales | d__Bacteria;p__Bacteroidota;c__Bacteroidia;o__Bacteroidales;f__Bacteroidaceae;g__Prevotella;s__ |  |  |  |  |
| bin.529 | 219 | 9024.61 | 1976389 | 12844 | 44.7011 |  | 86.07 | 2.721 | Clostridia | d__Bacteria;p__Firmicutes_B;c__Dehalobacteriia;o__UBA7702;f__UBA7702;g__;s__ | 0.057388 | 0.110821 | 0.030753 | 0.082555 |
| bin.53 | 627 | 5453.59 | 3419403 | 6691 | 43.5248 |  | 83.81 | 0.645 | Bacteria | d__Bacteria;p__Bacteroidota;c__Bacteroidia;o__Bacteroidales;f__Marinifilaceae;g__Odoribacter;s__Odoribacter splanchnicus |  |  |  |  |
| bin.530 | 527 | 2943.48 | 1551215 | 3057 | 63.954 |  | 64.86 | 2.873 | Clostridiales | d__Bacteria;p__Firmicutes_A;c__Clostridia;o__Oscillospirales;f__Oscillospiraceae;g__NK3B98;s__ | 0.216136 | 0.417378 | 0.161089 | 0.432442 |
| bin.531 | 311 | 5303.9 | 1649514 | 7096 | 35.2724 |  | 83.83 | 1.661 | Bacteria | d__Bacteria;p__Cyanobacteria;c__Vampirovibrionia;o__Gastranaerophilales;f__RUG14156;g__;s__ | 0.108961 | 0.210414 | 0.084007 | 0.225517 |
| bin.532 | 1266 | 1974.62 | 2499869 | 1999 | 56.695 |  | 56.33 | 4.509 | Clostridiales | d__Bacteria;p__Firmicutes_A;c__Clostridia;o__Oscillospirales;f__Oscillospiraceae;g__;s__ | 0.066486 | 0.12839 | 0.048178 | 0.129333 |
| bin.534 | 434 | 3780.74 | 1640842 | 4264 | 51.3196 |  | 75.79 | 0.377 | Bacteroidales | d__Bacteria;p__Bacteroidota;c__Bacteroidia;o__Bacteroidales;f__Barnesiellaceae;g__Barnesiella;s__Barnesiella sp900542255 |  |  |  |  |
| bin.535 | 623 | 4135.39 | 2576348 | 5663 | 49.5774 |  | 86.8 | 1.708 | Clostridiales | d__Bacteria;p__Firmicutes_A;c__Clostridia;o__Lachnospirales;f__Lachnospiraceae;g__;s__ | 0.048251 | 0.093178 |  |  |
| bin.536 | 586 | 2072.76 | 1214640 | 2237 | 69.4129 |  | 59.14 | 0.675 | Bacteria | d__Bacteria;p__Verrucomicrobiota;c__Kiritimatiellae;o__RFP12;f__UBA1067;g__W1P29-020;s__ |  |  |  |  |
| bin.537 | 443 | 4381.05 | 1940805 | 5380 | 41.297 |  | 90.82 | 2.706 | Clostridiales | d__Bacteria;p__Firmicutes_A;c__Clostridia;o__Lachnospirales;f__Lachnospiraceae;g__992a;s__ | 0.135919 | 0.262472 | 0.084307 | 0.226322 |
| bin.538 | 290 | 5746.22 | 1666404 | 8445 | 52.8079 |  | 73.58 | 0.952 | Bacteroidetes | d__Bacteria;p__Bacteroidota;c__Bacteroidia;o__Bacteroidales;f__UBA932;g__RC9;s__ |  |  |  |  |
| bin.539 | 496 | 1912.28 | 948489 | 1997 | 50.3898 |  | 61.61 | 1.88 | Lactobacillales | d__Bacteria;p__Firmicutes;c__Bacilli;o__Lactobacillales;f__Lactobacillaceae;g__Ligilactobacillus;s__ | 0.096132 | 0.185639 | 0.064771 | 0.173877 |
| bin.54 | 329 | 4746.71 | 1561668 | 5868 | 58.0047 |  | 73.8 | 1.111 | Bacteroidetes | d__Bacteria;p__Bacteroidota;c__Bacteroidia;o__Bacteroidales;f__UBA932;g__RC9;s__ |  |  |  |  |
| bin.540 | 688 | 2653.99 | 1825944 | 3166 | 32.4943 |  | 83.23 | 3.931 | Bacteria | d__Bacteria;p__Deferribacterota;c__Deferribacteres;o__Deferribacterales;f__Mucispirillaceae;g__Mucispirillum;s__ |  |  |  |  |
| bin.541 | 463 | 5316.07 | 2461339 | 6566 | 53.8158 |  | 81.85 | 1.16 | Clostridiales | d__Bacteria;p__Firmicutes_A;c__Clostridia;o__Lachnospirales;f__Lachnospiraceae;g__Enterocloster;s__ |  |  |  |  |
| bin.542 | 779 | 3055.37 | 2380134 | 3237 | 65.5361 |  | 65.99 | 0.38 | Actinobacteria | d__Bacteria;p__Actinobacteriota;c__Coriobacteriia;o__Coriobacteriales;f__Eggerthellaceae;g__Rubneribacter;s__Rubneribacter badeniensis | 0.0938 | 0.181136 | 0.049594 | 0.133134 |
| bin.543 | 363 | 4028.92 | 1462497 | 4657 | 56.0992 |  | 91.52 | 4.864 | Clostridia | d__Bacteria;p__Firmicutes_A;c__Clostridia_A;o__Christensenellales;f__Borkfalkiaceae;g__UMGS775;s__ | 0.146434 | 0.282776 | 0.127249 | 0.341597 |
| bin.544 | 548 | 1911.41 | 1047453 | 1889 | 51.7614 |  | 51.53 | 1.015 | Prevotella | d__Bacteria;p__Bacteroidota;c__Bacteroidia;o__Bacteroidales;f__Bacteroidaceae;g__Prevotella;s__Prevotella sp000435635 | 0.078776 | 0.152123 | 0.054191 | 0.145474 |
| bin.545 | 578 | 2600.41 | 1503039 | 2616 | 62.8495 |  | 59.98 | 0.595 | Bacteria | d__Bacteria;p__Firmicutes_A;c__Clostridia;o__Oscillospirales;f__Ruminococcaceae;g__Gemmiger_A;s__Gemmiger_A sp002160955 |  |  |  |  |
| bin.546 | 533 | 3170.68 | 1689974 | 3425 | 43.4214 |  | 66.46 | 0.279 | Clostridiales | d__Bacteria;p__Firmicutes_A;c__Clostridia;o__Oscillospirales;f__CAG-382;g__UMGS882;s__UMGS882 sp003343885 | 0.173965 | 0.335941 | 0.12887 | 0.345949 |
| bin.547 | 420 | 4812.64 | 2021309 | 5957 | 32.9103 |  | 91.03 | 1.415 | Bacteria | d__Bacteria;p__Firmicutes;c__Bacilli;o__Erysipelotrichales;f__Erysipelotrichaceae;g__Clostridium_AQ;s__ | 0.099575 | 0.192288 | 0.043949 | 0.117981 |
| bin.548 | 312 | 7598.1 | 2370607 | 11040 | 61.8569 |  | 92.55 | 3.427 | Clostridia | d__Bacteria;p__Firmicutes_A;c__Clostridia_A;o__Christensenellales;f__CAG-74;g__Firm-11;s__ | 0.213476 | 0.412241 |  |  |
| bin.549 | 381 | 3729.74 | 1421031 | 4115 | 62.215 |  | 68.88 | 0.824 | Clostridiales | d__Bacteria;p__Firmicutes_A;c__Clostridia;o__Oscillospirales;f__Oscillospiraceae;g__;s__ |  |  |  |  |
| bin.55 | 274 | 3283.89 | 899785 | 3913 | 27.7357 |  | 62.26 | 1.685 | Bacteria | d__Bacteria;p__Firmicutes;c__Bacilli;o__RF39;f__UBA660;g__CAG-822;s__ |  |  |  |  |
| bin.550 | 175 | 7970.1 | 1394768 | 11592 | 41.8203 |  | 92.38 | 0.314 | Bacteria | d__Bacteria;p__Firmicutes;c__Bacilli;o__Erysipelotrichales;f__Erysipelotrichaceae;g__Faecalicoccus;s__ |  |  |  |  |
| bin.551 | 228 | 9145.87 | 2085258 | 12180 | 58.703 |  | 81.03 | 6.034 | Bacteria | d__Bacteria;p__Firmicutes_A;c__Clostridia;o__Oscillospirales;f__Oscillospiraceae;g__CAG-110;s__ |  |  |  |  |
| bin.552 | 367 | 3066.66 | 1125466 | 3880 | 27.2421 |  | 81.67 | 1.685 | Bacteria | d__Bacteria;p__Firmicutes;c__Bacilli;o__RFN20;f__CAG-826;g__;s__ |  |  |  |  |
| bin.553 | 688 | 1899.12 | 1306592 | 1988 | 59.1435 |  | 55.72 | 0 | Bacteria | d__Bacteria;p__Bacteroidota;c__Bacteroidia;o__Bacteroidales;f__Paludibacteraceae;g__QVMH01;s__ |  |  |  |  |
| bin.554 | 267 | 9326.73 | 2490236 | 13723 | 53.0046 |  | 65.86 | 1.239 | Bacteroidales | d__Bacteria;p__Bacteroidota;c__Bacteroidia;o__Bacteroidales;f__Bacteroidaceae;g__Prevotellamassilia;s__Prevotellamassilia sp900752815 | 0.044014 | 0.084995 | 0.029564 | 0.079363 |
| bin.555 | 410 | 2888.99 | 1184486 | 3070 | 44.2462 |  | 56.9 | 0.952 | Bacteria | d__Bacteria;p__Firmicutes;c__Bacilli;o__Erysipelotrichales;f__Erysipelotrichaceae;g__Bulleidia;s__ | 0.245013 | 0.473142 | 0.223217 | 0.599223 |
| bin.556 | 762 | 2120.58 | 1615881 | 2303 | 49.2494 |  | 53.21 | 1.652 | Lachnospiraceae | d__Bacteria;p__Firmicutes_A;c__Clostridia;o__Lachnospirales;f__Lachnospiraceae;g__Acetatifactor;s__ |  |  |  |  |
| bin.557 | 320 | 4048.96 | 1295668 | 4844 | 48.6909 |  | 85.23 | 2.517 | Clostridiales | d__Bacteria;p__Firmicutes_A;c__Clostridia_A;o__Christensenellales;f__CAG-917;g__CAG-1138;s__ | 0.144218 | 0.278496 | 0.107396 | 0.288303 |
| bin.558 | 610 | 2596.14 | 1583645 | 3090 | 48.4218 |  | 82.52 | 3.054 | Bacteroidales | d__Bacteria;p__Bacteroidota;c__Bacteroidia;o__Bacteroidales;f__Muribaculaceae;g__CAG-279;s__CAG-279 sp900541555 | 0.04774 | 0.092191 | 0.025203 | 0.067656 |
| bin.559 | 477 | 4510.35 | 2151438 | 5570 | 54.2919 |  | 52.35 | 0 | Bacteria | d__Bacteria;p__Bacteroidota;c__Bacteroidia;o__Bacteroidales;f__Bacteroidaceae;g__CAG-462;s__CAG-462 sp900291465 |  |  |  |  |
| bin.56 | 235 | 6904.11 | 1622466 | 10556 | 33.4325 |  | 78.66 | 1.123 | Bacteria | d__Bacteria;p__Cyanobacteria;c__Vampirovibrionia;o__Gastranaerophilales;f__RUG14156;g__;s__ |  |  |  |  |
| bin.560 | 431 | 1785.26 | 769448 | 1843 | 36.2568 |  | 52.81 | 1.347 | Bacteria | d__Bacteria;p__Firmicutes;c__Bacilli;o__Erysipelotrichales;f__Erysipelotrichaceae;g__Faecalitalea;s__Faecalitalea cylindroides | 0.170738 | 0.329711 | 0.113289 | 0.304122 |
| bin.561 | 670 | 3291.88 | 2205559 | 4257 | 49.8547 |  | 89.16 | 3.858 | Bacteroidales | d__Bacteria;p__Bacteroidota;c__Bacteroidia;o__Bacteroidales;f__Barnesiellaceae;g__Barnesiella;s__ |  |  |  |  |
| bin.562 | 528 | 3832.85 | 2023743 | 5355 | 43.4073 |  | 82.87 | 1.003 | Bacteroidales | d__Bacteria;p__Bacteroidota;c__Bacteroidia;o__Bacteroidales;f__Bacteroidaceae;g__Paraprevotella;s__ | 0.027671 | 0.053435 | 0.013925 | 0.037381 |
| bin.563 | 300 | 6683.32 | 2004997 | 12161 | 45.2225 |  | 89.91 | 0 | Clostridia | d__Bacteria;p__Firmicutes_A;c__Clostridia_A;o__Christensenellales;f__CAG-314;g__UMGS929;s__UMGS929 sp900546875 | 0.18842 | 0.363855 | 0.144258 | 0.387258 |
| bin.564 | 333 | 4303.55 | 1433083 | 5257 | 52.9191 |  | 60.28 | 1.754 | Bacteria | d__Bacteria;p__Firmicutes_A;c__Clostridia;o__Lachnospirales;f__Lachnospiraceae;g__OF09-33XD;s__ |  |  |  |  |
| bin.565 | 347 | 3012.73 | 1045419 | 3317 | 45.715 |  | 73.78 | 2.471 | Clostridia | d__Bacteria;p__Firmicutes_A;c__Clostridia_A;o__Christensenellales;f__DTU072;g__CAG-1782;s__ | 0.079892 | 0.154278 | 0.078118 | 0.209707 |
| bin.566 | 433 | 4194.11 | 1816050 | 6139 | 57.1684 |  | 88.53 | 1.957 | Bacteroidetes | d__Bacteria;p__Bacteroidota;c__Bacteroidia;o__Bacteroidales;f__UBA932;g__RC9;s__RC9 sp900543205 |  |  |  |  |
| bin.567 | 509 | 2712.86 | 1380848 | 2767 | 63.4691 |  | 51.37 | 1.612 | Clostridia | d__Bacteria;p__Firmicutes_A;c__Clostridia_A;o__Christensenellales;f__UBA1750;g__;s__ |  |  |  |  |
| bin.568 | 64 | 30716.1 | 1965828 | 85165 | 55.0456 |  | 99.3 | 0 | Spirochaetaceae | d__Bacteria;p__Spirochaetota;c__Spirochaetia;o__Treponematales;f__Treponemataceae;g__;s__ | 0.135886 | 0.262408 | 0.097348 | 0.261331 |
| bin.569 | 561 | 2662.07 | 1493420 | 3099 | 43.0158 |  | 54.4 | 0.832 | Clostridia | d__Bacteria;p__Firmicutes_A;c__Clostridia_A;o__Christensenellales;f__DTU072;g__CAG-1782;s__ | 0.115779 | 0.223579 | 0.082899 | 0.22254 |
| bin.57 | 406 | 4798.57 | 1948219 | 6121 | 28.2881 |  | 84.88 | 3.483 | Bacteria | d__Bacteria;p__Fusobacteriota;c__Fusobacteriia;o__Fusobacteriales;f__Fusobacteriaceae;g__Fusobacterium_A;s__ |  |  |  |  |
| bin.570 | 383 | 3932.7 | 1506225 | 4613 | 34.2135 |  | 80.16 | 2.089 | Bacteria | d__Bacteria;p__Cyanobacteria;c__Vampirovibrionia;o__Gastranaerophilales;f__RUG14156;g__;s__ | 0.130319 | 0.251657 | 0.087521 | 0.234949 |
| bin.571 | 543 | 4704.43 | 2554506 | 5776 | 39.0254 |  | 86.42 | 2.692 | Bacteroidales | d__Bacteria;p__Bacteroidota;c__Bacteroidia;o__Bacteroidales;f__Tannerellaceae;g__Parabacteroides;s__ |  |  |  |  |
| bin.572 | 224 | 8706.09 | 1950165 | 12839 | 47.2486 |  | 83.8 | 0.643 | Lachnospiraceae | d__Bacteria;p__Firmicutes_A;c__Clostridia;o__Lachnospirales;f__Lachnospiraceae;g__Mediterraneibacter;s__ |  |  |  |  |
| bin.573 | 656 | 2359.01 | 1547508 | 2625 | 48.574 |  | 68.29 | 2.007 | Lachnospiraceae | d__Bacteria;p__Firmicutes_A;c__Clostridia;o__Lachnospirales;f__Lachnospiraceae;g__Mediterraneibacter;s__ |  |  |  |  |
| bin.574 | 399 | 3894 | 1553707 | 4526 | 61.2418 |  | 75.49 | 2.013 | Clostridiales | d__Bacteria;p__Firmicutes_A;c__Clostridia_A;o__Christensenellales;f__;g__;s__ | 0.157038 | 0.303253 | 0.109851 | 0.294895 |
| bin.575 | 494 | 3034.34 | 1498964 | 3347 | 53.2081 |  | 62 | 1.724 | Bacteria | d__Bacteria;p__Bacteroidota;c__Bacteroidia;o__Bacteroidales;f__Rikenellaceae;g__Alistipes_A;s__ |  |  |  |  |
| bin.576 | 526 | 2394.11 | 1259300 | 2755 | 52.2328 |  | 67.01 | 2.125 | Clostridiales | d__Bacteria;p__Firmicutes_A;c__Clostridia_A;o__Christensenellales;f__Borkfalkiaceae;g__;s__ |  |  |  |  |
| bin.577 | 321 | 2548.97 | 818219 | 2529 | 66.9648 |  | 57.59 | 1.478 | Actinobacteria | d__Bacteria;p__Actinobacteriota;c__Coriobacteriia;o__Coriobacteriales;f__Eggerthellaceae;g__;s__ | 0.096845 | 0.187016 | 0.079666 | 0.213861 |
| bin.578 | 284 | 3213.47 | 912625 | 3522 | 59.089 |  | 55.45 | 2.727 | Bacteria | d__Bacteria;p__Firmicutes_A;c__Clostridia_A;o__Christensenellales;f__CAG-917;g__UMGS1688;s__ |  |  |  |  |
| bin.579 | 421 | 4767.29 | 2007028 | 7017 | 51.5133 |  | 87.81 | 1.666 | Bacteroidetes | d__Bacteria;p__Bacteroidota;c__Bacteroidia;o__Bacteroidales;f__UBA932;g__RC9;s__ |  |  |  |  |
| bin.58 | 294 | 3652.24 | 1073760 | 3985 | 58.9229 |  | 56.22 | 0 | Bacteria | d__Bacteria;p__Firmicutes_A;c__Clostridia;o__Oscillospirales;f__Ruminococcaceae;g__Pygmaiobacter;s__ |  |  |  |  |
| bin.580 | 397 | 3149.43 | 1250323 | 3363 | 37.4281 |  | 58.68 | 7.142 | Lactobacillales | d__Bacteria;p__Firmicutes;c__Bacilli;o__Lactobacillales;f__Aerococcaceae;g__Facklamia_A;s__Facklamia_A tabacinasalis | 0.175365 | 0.338645 | 0.137196 | 0.368301 |
| bin.581 | 425 | 4555.21 | 1935963 | 5641 | 48.9613 |  | 79.48 | 1.137 | Bacteroidetes | d__Bacteria;p__Bacteroidota;c__Bacteroidia;o__Bacteroidales;f__Rikenellaceae;g__Alistipes_A;s__ |  |  |  |  |
| bin.582 | 493 | 6720.51 | 3313213 | 9744 | 35.812 |  | 97.46 | 1.898 | Clostridiales | d__Bacteria;p__Firmicutes_A;c__Clostridia;o__Lachnospirales;f__Lachnospiraceae;g__CHKCI001;s__ | 0.098224 | 0.18968 | 0.064038 | 0.171909 |
| bin.583 | 226 | 2587.39 | 584750 | 2680 | 27.8963 |  | 51.23 | 1.818 | Bacteria | d__Bacteria;p__Firmicutes_A;c__Clostridia;o__TANB77;f__CAG-508;g__CAG-269;s__ |  |  |  |  |
| bin.584 | 513 | 3438.58 | 1763992 | 4397 | 58.9077 |  | 89.32 | 1.588 | Clostridiales | d__Bacteria;p__Firmicutes_A;c__Clostridia;o__Oscillospirales;f__Oscillospiraceae;g__CAG-110;s__ | 0.059063 | 0.114056 | 0.019594 | 0.0526 |
| bin.585 | 335 | 3437.1 | 1151428 | 4585 | 34.95 |  | 89.13 | 1.221 | Lactobacillales | d__Bacteria;p__Firmicutes;c__Bacilli;o__Lactobacillales;f__Lactobacillaceae;g__;s__ | 0.114743 | 0.221578 | 0.09822 | 0.263671 |
| bin.586 | 342 | 7612.04 | 2603318 | 10785 | 46.7823 |  | 94.08 | 3.485 | Clostridiales | d__Bacteria;p__Firmicutes_A;c__Clostridia;o__Lachnospirales;f__Lachnospiraceae;g__Blautia_A;s__ | 0.07055 | 0.136239 | 0.069908 | 0.187667 |
| bin.587 | 193 | 4169.9 | 804790 | 4842 | 35.8404 |  | 71.49 | 1.123 | Bacteria | d__Bacteria;p__Firmicutes_A;c__Clostridia_A;o__Christensenellales;f__UBA1242;g__UBA11517;s__ |  |  |  |  |
| bin.588 | 513 | 3857.27 | 1978780 | 4229 | 56.4859 |  | 66.28 | 2.027 | Bacteria | d__Bacteria;p__Verrucomicrobiota;c__Lentisphaeria;o__Victivallales;f__Victivallaceae;g__UBA1776;s__ | 0.147473 | 0.284784 |  |  |
| bin.589 | 481 | 4482.58 | 2156123 | 5769 | 38.7347 |  | 87.54 | 0.588 | Gammaproteobacteria | d__Bacteria;p__Proteobacteria;c__Gammaproteobacteria;o__Enterobacterales;f__Succinivibrionaceae;g__Anaerobiospirillum_A;s__ |  |  |  |  |
| bin.59 | 381 | 3305.36 | 1259343 | 3651 | 31.9043 |  | 53.99 | 0.427 | Bacteria | d__Bacteria;p__Cyanobacteria;c__Vampirovibrionia;o__Gastranaerophilales;f__RUG14156;g__;s__ |  |  |  |  |
| bin.590 | 453 | 4961.22 | 2247432 | 6443 | 56.3348 |  | 90.45 | 0 | Bacteria | d__Bacteria;p__Verrucomicrobiota;c__Verrucomicrobiae;o__Verrucomicrobiales;f__Akkermansiaceae;g__Akkermansia;s__Akkermansia muciniphila | 0.12328 | 0.238064 | 0.068514 | 0.183925 |
| bin.591 | 143 | 5152.76 | 736845 | 6939 | 49.7486 |  | 57.83 | 3.418 | Bacteria | d__Bacteria;p__Patescibacteria;c__Saccharimonadia;o__Saccharimonadales;f__Saccharimonadaceae;g__UBA1103;s__ | 0.667688 | 1.289363 | 0.584641 | 1.56946 |
| bin.6 | 687 | 2037.62 | 1399844 | 2174 | 32.0156 |  | 78.48 | 2.689 | Firmicutes | d__Bacteria;p__Firmicutes_C;c__Negativicutes;o__Selenomonadales;f__Selenomonadaceae;g__Megamonas;s__Megamonas funiformis | 0.159311 | 0.307642 | 0.153068 | 0.410908 |
| bin.60 | 535 | 2009.26 | 1074955 | 2072 | 34.0634 |  | 56.42 | 1.503 | Campylobacterales | d__Bacteria;p__Campylobacterota;c__Campylobacteria;o__Campylobacterales;f__Helicobacteraceae;g__Helicobacter_D;s__Helicobacter_D pullorum | 0.13837 | 0.267205 | 0.089219 | 0.239507 |
| bin.61 | 444 | 3231.25 | 1434677 | 3426 | 60.4873 |  | 72.24 | 0.806 | Actinobacteria | d__Bacteria;p__Actinobacteriota;c__Coriobacteriia;o__Coriobacteriales;f__Coriobacteriaceae;g__An2-A;s__ |  |  |  |  |
| bin.62 | 248 | 3498.51 | 867631 | 4209 | 36.7576 |  | 59.56 | 1.016 | Clostridiales | d__Bacteria;p__Firmicutes_A;c__Clostridia_A;o__Christensenellales;f__UBA1242;g__UBA11517;s__ | 0.415778 | 0.802904 | 0.350263 | 0.940276 |
| bin.63 | 459 | 3776.18 | 1733265 | 4340 | 65.6696 |  | 78.32 | 0.806 | Clostridia | d__Bacteria;p__Firmicutes_A;c__Clostridia_A;o__Christensenellales;f__CAG-74;g__HGM11575;s__ |  |  |  |  |
| bin.64 | 538 | 1976.86 | 1063550 | 2083 | 45.1187 |  | 63.57 | 1.612 | Actinobacteria | d__Bacteria;p__Actinobacteriota;c__Coriobacteriia;o__Coriobacteriales;f__Eggerthellaceae;g__CAG-1427;s__CAG-1427 sp900548955 |  |  |  |  |
| bin.65 | 874 | 3531.19 | 3086261 | 4640 | 49.2187 |  | 92.34 | 1.582 | Clostridiales | d__Bacteria;p__Firmicutes_A;c__Clostridia;o__Lachnospirales;f__Lachnospiraceae;g__Blautia_A;s__ |  |  |  |  |
| bin.66 | 455 | 4510.53 | 2052291 | 5514 | 45.9779 |  | 80.06 | 0.684 | Bacteria | d__Bacteria;p__Bacteroidota;c__Bacteroidia;o__Bacteroidales;f__Marinifilaceae;g__Butyricimonas;s__ | 0.012992 | 0.025089 | 0.014897 | 0.03999 |
| bin.67 | 447 | 4492.45 | 2008124 | 5395 | 48.5644 |  | 80.79 | 0.641 | Bacteroidales | d__Bacteria;p__Bacteroidota;c__Bacteroidia;o__Bacteroidales;f__Muribaculaceae;g__;s__ | 0.020046 | 0.038711 | 0.009033 | 0.02425 |
| bin.68 | 658 | 2939.81 | 1934394 | 3672 | 42.0809 |  | 77.19 | 4.032 | Bacteria | d__Bacteria;p__Bacteroidota;c__Bacteroidia;o__Bacteroidales;f__Paludibacteraceae;g__;s__ | 0.084267 | 0.162727 | 0.045401 | 0.121877 |
| bin.69 | 753 | 1925.59 | 1449968 | 1983 | 51.2985 |  | 56.5 | 1.759 | Bacteroidales | d__Bacteria;p__Bacteroidota;c__Bacteroidia;o__Bacteroidales;f__Bacteroidaceae;g__Prevotella;s__Prevotella lascolaii | 0.053224 | 0.102779 | 0.033845 | 0.090856 |
| bin.7 | 548 | 3221.43 | 1765346 | 4574 | 51.744 |  | 79.58 | 2.396 | Bacteroidetes | d__Bacteria;p__Bacteroidota;c__Bacteroidia;o__Bacteroidales;f__UBA932;g__RC9;s__ |  |  |  |  |
| bin.70 | 549 | 4560.39 | 2503655 | 5531 | 59.1898 |  | 93.27 | 3.251 | Clostridia | d__Bacteria;p__Firmicutes_A;c__Clostridia_A;o__Christensenellales;f__CAG-74;g__JAAYOH01;s__ | 0.070603 | 0.136341 | 0.066588 | 0.178755 |
| bin.71 | 752 | 2604 | 1958206 | 3182 | 49.8334 |  | 85.04 | 2.979 | Clostridiales | d__Bacteria;p__Firmicutes_A;c__Clostridia;o__Monoglobales_A;f__UBA1381;g__;s__ |  |  |  |  |
| bin.72 | 859 | 2784.28 | 2391698 | 3253 | 50.8695 |  | 53.58 | 6.896 | Bacteria | d__Bacteria;p__Firmicutes_A;c__Clostridia;o__Lachnospirales;f__Lachnospiraceae;g__Mediterraneibacter;s__ |  |  |  |  |
| bin.73 | 604 | 2067.7 | 1248891 | 2225 | 60.4813 |  | 73.1 | 1.203 | Bacteria | d__Bacteria;p__Spirochaetota;c__Spirochaetia;o__Sphaerochaetales;f__Sphaerochaetaceae;g__;s__ | 0.034871 | 0.067339 | 0.028534 | 0.0766 |
| bin.74 | 491 | 3504.14 | 1720535 | 3813 | 50.8055 |  | 52.89 | 1.724 | Bacteria | d__Bacteria;p__Bacteroidota;c__Bacteroidia;o__Bacteroidales;f__UBA932;g__RC9;s__ |  |  |  |  |
| bin.75 | 736 | 2957.49 | 2176711 | 3705 | 40.6076 |  | 91.47 | 3.638 | Spirochaetaceae | d__Bacteria;p__Spirochaetota;c__Spirochaetia;o__Treponematales;f__Treponemataceae;g__;s__ | 0.147451 | 0.28474 | 0.103214 | 0.277077 |
| bin.76 | 209 | 7118.12 | 1487687 | 9574 | 49.6232 |  | 90.3 | 2.445 | Lactobacillales | d__Bacteria;p__Firmicutes;c__Bacilli;o__Lactobacillales;f__Lactobacillaceae;g__Limosilactobacillus;s__ |  |  |  |  |
| bin.77 | 376 | 4223.04 | 1587864 | 5188 | 60.4722 |  | 79.4 | 1.738 | Clostridiales | d__Bacteria;p__Firmicutes_A;c__Clostridia;o__Oscillospirales;f__Oscillospiraceae;g__;s__ |  |  |  |  |
| bin.78 | 917 | 1957.18 | 1794732 | 2086 | 51.1488 |  | 58.63 | 1.597 | Clostridiales | d__Bacteria;p__Firmicutes_A;c__Clostridia;o__Oscillospirales;f__Ruminococcaceae;g__UBA1448;s__ | 0.191843 | 0.370466 | 0.130264 | 0.349691 |
| bin.79 | 381 | 3352.58 | 1277334 | 3706 | 52.2341 |  | 71.54 | 1.209 | Clostridia | d__Bacteria;p__Firmicutes_A;c__Clostridia_A;o__Christensenellales;f__Borkfalkiaceae;g__UBA11940;s__UBA11940 sp900549005 | 0.019344 | 0.037355 | 0.058908 | 0.158137 |
| bin.8 | 309 | 7493.43 | 2315470 | 10660 | 47.1107 |  | 89.6 | 1.572 | Bacteroidales | d__Bacteria;p__Bacteroidota;c__Bacteroidia;o__Bacteroidales;f__Muribaculaceae;g__;s__ | 0.036117 | 0.069746 | 0.024582 | 0.065991 |
| bin.80 | 363 | 3695.93 | 1341622 | 4232 | 60.2766 |  | 55.77 | 1.881 | Clostridia | d__Bacteria;p__Firmicutes_A;c__Clostridia_A;o__Christensenellales;f__CAG-74;g__SFFH01;s__ |  |  |  |  |
| bin.81 | 357 | 3052.01 | 1089569 | 3229 | 46.8388 |  | 58.64 | 1.149 | Bacteria | d__Bacteria;p__Spirochaetota;c__Spirochaetia;o__Sphaerochaetales;f__Sphaerochaetaceae;g__;s__ |  |  |  |  |
| bin.82 | 353 | 3708.76 | 1309194 | 4230 | 50.6343 |  | 50.56 | 3.448 | Bacteria | d__Bacteria;p__Bacteroidota;c__Bacteroidia;o__Bacteroidales;f__UBA932;g__RC9;s__ |  |  |  |  |
| bin.83 | 996 | 2620.41 | 2609933 | 3090 | 49.8456 |  | 59.95 | 2.586 | Bacteria | d__Bacteria;p__Firmicutes_A;c__Clostridia;o__Lachnospirales;f__Lachnospiraceae;g__Mediterraneibacter;s__ |  |  |  |  |
| bin.84 | 250 | 4494.94 | 1123734 | 5465 | 58.4142 |  | 79.62 | 0.12 | Firmicutes | d__Bacteria;p__Firmicutes_A;c__Clostridia_A;o__Christensenellales;f__CAG-917;g__UMGS1688;s__ | 0.067869 | 0.13106 |  |  |
| bin.85 | 378 | 4728.06 | 1787208 | 5659 | 58.1646 |  | 61.52 | 0 | Bacteria | d__Bacteria;p__Firmicutes_A;c__Clostridia;o__Oscillospirales;f__Oscillospiraceae;g__CAG-110;s__ |  |  |  |  |
| bin.86 | 395 | 2666.87 | 1053415 | 2794 | 39.1696 |  | 50.05 | 1.123 | Bacteria | d__Bacteria;p__Cyanobacteria;c__Vampirovibrionia;o__Gastranaerophilales;f__RUG14156;g__;s__ |  |  |  |  |
| bin.87 | 201 | 9993.92 | 2008778 | 12475 | 50.627 |  | 89.1 | 1.666 | Bacteroidetes | d__Bacteria;p__Bacteroidota;c__Bacteroidia;o__Bacteroidales;f__UBA932;g__RC9;s__ |  |  |  |  |
| bin.88 | 158 | 5694.06 | 899662 | 7553 | 31.7032 |  | 83.7 | 1.235 | Bacteria | d__Bacteria;p__Firmicutes;c__Bacilli;o__RFN20;f__CAG-288;g__UBA7642;s__ | 0.482352 | 0.931464 | 0.418701 | 1.123997 |
| bin.89 | 696 | 2332.09 | 1623138 | 2674 | 47.6917 |  | 50.63 | 0 | Bacteria | d__Bacteria;p__Firmicutes_A;c__Clostridia;o__Lachnospirales;f__Lachnospiraceae;g__Anaerobutyricum;s__Anaerobutyricum sp900016875 |  |  |  |  |
| bin.9 | 419 | 4069.27 | 1705024 | 4696 | 60.9706 |  | 66.9 | 0.167 | Clostridiales | d__Bacteria;p__Firmicutes_A;c__Clostridia;o__Oscillospirales;f__Acutalibacteraceae;g__UMGS856;s__ | 0.154184 | 0.297742 | 0.095661 | 0.2568 |
| bin.90 | 217 | 8230.45 | 1786008 | 11474 | 51.362 |  | 87.56 | 1.931 | Bacteroidetes | d__Bacteria;p__Bacteroidota;c__Bacteroidia;o__Bacteroidales;f__UBA932;g__RC9;s__ |  |  | 0.037374 | 0.10033 |
| bin.91 | 476 | 3101.44 | 1476285 | 3402 | 61.4711 |  | 53.4 | 1.901 | Clostridiales | d__Bacteria;p__Firmicutes_A;c__Clostridia;o__Oscillospirales;f__Acutalibacteraceae;g__Hydrogeniiclostridium;s__ |  |  |  |  |
| bin.92 | 45 | 35493.2 | 1597194 | 50538 | 63.9992 |  | 94.16 | 0.069 | Actinobacteria | d__Bacteria;p__Actinobacteriota;c__Actinomycetia;o__Actinomycetales;f__Bifidobacteriaceae;g__Aeriscardovia;s__ | 0.329278 | 0.635864 | 0.265948 | 0.713932 |
| bin.93 | 938 | 2590.53 | 2429919 | 3005 | 28.6378 |  | 89.01 | 0.807 | Spirochaetia | d__Bacteria;p__Spirochaetota;c__Brachyspirae;o__Brachyspirales;f__Brachyspiraceae;g__Brachyspira;s__Brachyspira innocens |  |  |  |  |
| bin.94 | 554 | 3039.16 | 1683694 | 3703 | 48.0973 |  | 85.26 | 3.207 | Bacteroidales | d__Bacteria;p__Bacteroidota;c__Bacteroidia;o__Bacteroidales;f__Bacteroidaceae;g__;s__ | 0.078937 | 0.152434 | 0.049158 | 0.131963 |
| bin.95 | 613 | 2712.48 | 1662752 | 3261 | 40.2457 |  | 83.62 | 0.582 | Spirochaetaceae | d__Bacteria;p__Spirochaetota;c__Spirochaetia;o__Treponematales;f__Treponemataceae;g__Treponema_F;s__ | 0.140911 | 0.272111 | 0.107242 | 0.287889 |
| bin.96 | 694 | 1934.21 | 1342341 | 2009 | 42.7436 |  | 60.45 | 1.799 | Clostridiales | d__Bacteria;p__Firmicutes_A;c__Clostridia;o__Oscillospirales;f__Ruminococcaceae;g__Ruminiclostridium_E;s__ | 0.212785 | 0.410905 | 0.118823 | 0.318979 |
| bin.97 | 664 | 4836.45 | 3211405 | 6682 | 47.9239 |  | 83.67 | 2.338 | Bacteroidales | d__Bacteria;p__Bacteroidota;c__Bacteroidia;o__Bacteroidales;f__Bacteroidaceae;g__Bacteroides;s__Bacteroides uniformis |  |  |  |  |
| bin.98 | 538 | 3098.44 | 1666962 | 3881 | 41.7273 |  | 80.29 | 2.391 | Clostridia | d__Bacteria;p__Firmicutes_B;c__Dehalobacteriia;o__UBA7702;f__UBA7702;g__;s__ | 0.08365 | 0.161536 | 0.044743 | 0.120112 |
| bin.99 | 233 | 2391.95 | 557324 | 2408 | 30.472 |  | 61.49 | 2.969 | Bacteria | d__Bacteria;p__Firmicutes;c__Bacilli;o__RF39;f__UBA660;g__CAG-594;s__ | 0.261159 | 0.50432 |  |  |

**References**

[1] L.A. Hug, B.J. Baker, K. Anantharaman, C.T. Brown, A.J. Probst, C.J. Castelle, C.N. Butterfield, A.W. Hernsdorf, Y. Amano, K. Ise, Y. Suzuki, N. Dudek, D.A. Relman, K.M. Finstad, R. Amundson, B.C. Thomas, J.F. Banfield, A new view of the tree of life, Nat. Microbiol. 2016 15 1 (2016) 1–6. https://doi.org/10.1038/nmicrobiol.2016.48.
